# *Diaphorina citri* Genome Possesses a Complete Melatonin Biosynthesis Pathway Differentially Expressed under the Influence of the Phytopathogenic Bacterium, *Candidatus* Liberibacter asiaticus

**DOI:** 10.3390/insects12040317

**Published:** 2021-04-01

**Authors:** Yasser Nehela, Nabil Killiny

**Affiliations:** 1 Citrus Research and Education Center, Department of Plant Pathology, University of Florida, 700 Experiment Station Rd., Lake Alfred, FL 33850, USA; yasser.nehela@ufl.edu; 2 Department of Agricultural Botany, Faculty of Agriculture, Tanta University, Tanta 31511, Egypt

**Keywords:** *Diaphorina citri*, *Candidatus* Liberibacter asiaticus, melatonin, citrus, tryptophan hydroxylase (TPH), aromatic L-amino acid decarboxylase (AADC), acetylserotonin methyltransferase (ASMT), serotonin N-acetyltransferase (SNAT), bioinformatics

## Abstract

**Simple Summary:**

The indole-like compound, melatonin, is a tryptophan-derivative that is secreted by the pineal gland. Melatonin is ubiquitously distributed in both prokaryotes and eukaryotes including animals and plants. In animals, melatonin plays pleiotropic regulatory roles in several biological and physiological functions including sleep, circadian rhythm, oxidative stress, immune response, aging, apoptosis, and autophagy. Moreover, it might have anti-inflammatory, anti-tumor, and anti-cancer activities. Although most, if not all, of these genes were cloned and characterized previously in numerous animal species, none of them have been identified from the Asian citrus psyllid, *Diaphorina citri*, in the vector of Huanglongbing yet. In the current study, we performed a genome-wide analysis and introduces a shortlist of six putative melatonin biosynthesis-related genes included two putative tryptophan 5-hydroxylase (*DcT5H*-1 and *DcT5H*-2), a putative aromatic amino acid decarboxylase (*DcAADC*), two putative arylalkylamine N-acetyltransferase (*DcAANAT*-1 and *DcAANAT*-2), and putative N-acetylserotonin O-methyltransferase (*DcASMT*), which could indicate sites of functional or structural constraint. All these genes were differentially expressed under the influence of the phytopathogenic bacterium, *Candidatus* Liberibacter asiaticus, and after melatonin supplementation. Our findings could be a further step for optimization and cloning of melatonin biosynthesis genes of *Diaphorina citri*.

**Abstract:**

Melatonin is synthesized from the amino acid _L_-tryptophan via the shikimic acid pathway and ubiquitously distributed in both prokaryotes and eukaryotes. Although most of melatonin biosynthesis genes were characterized in several plants and animal species including the insect model, *Drosophila melanogaster*, none of these enzymes have been identified from the Asian citrus psyllid, *Diaphorina citri*. We used comprehensive in silico analysis and gene expression techniques to identify the melatonin biosynthesis-related genes of *D. citri* and to evaluate the expression patterns of these genes within the adults of *D. citri* with gradient infection rates (0, 28, 34, 50, 58, and 70%) of the phytopathogenic bacterium *Candidatus* Liberibacter asiaticus and after the treatment with exogenous melatonin. We showed that the *D. citri* genome possesses six putative melatonin biosynthesis-related genes including two putative tryptophan 5-hydroxylase (*DcT5H*-1 and *DcT5H*-2), a putative aromatic amino acid decarboxylase (*DcAADC*), two putative arylalkylamine N-acetyltransferase (*DcAANAT*-1 and *DcAANAT*-2), and putative N-acetylserotonin O-methyltransferase (*DcASMT*). The infection with *Ca.* L. asiaticus decreased the transcript levels of all predicted genes in the adults of *D. citri*. Moreover, melatonin supplementation induced their expression levels in both healthy and *Ca.* L. asiaticus-infected psyllids. These findings confirm the association of these genes with the melatonin biosynthesis pathway.

## 1. Introduction

Huanglongbing (HLB; also known as citrus greening disease) is one of the most serious threatening diseases in citrus growing regions worldwide [1,2,3,4]. In the Americas, HLB was firstly confirmed in both South and North America (São Paulo, Brazil and Florida, USA, respectively) in 2004 and 2005, respectively [1,2,3]. Subsequently, it has been reported in Louisiana (2008), South Carolina and Georgia (2009), and Texas and California (2012) [3]. Additionally, it has been recorded in Caribbean countries included Belize, Cuba, Mexico, and Jamaica [3]. Recently, HLB turns out to be the most serious challenge for the citrus industry in Florida particularly, and the USA in general [5]. Diminishing the disease spread rate via reduction of vector populations has become one of the top priorities for the citrus industry, in California particularly, and in all citrus-growing areas generally [6,7,8].

HLB is associated with a fastidious, Gram-negative, phloem-limited, and non-culturable *α*-proteobacterium *Candidatus* Liberibacter spp. [1,2,3]. Based on the characteristic 16S rDNA sequence and geographical distribution, three Liberibacter species were proposed to be associated with HLB including *Ca.* L. africanus in Africa, *Ca.* L. americanus in Brazil, and *Ca.* L. asiaticus in Asia, Africa, and the Americas [1,2,3], with *Ca.* L. asiaticus being the most dominant and destructive species worldwide [1,2]. Although *Ca.* Liberibacter spp. can be transmitted by graft inoculation, they are mainly transmitted by citrus psyllid vectors [9]. The African psyllid citrus, *Trioza erytreae* Del Guercio (Hemiptera: Triozidae) transmits the *Ca.* L. africanus, while the Asian citrus psyllid, *Diaphorina citri* Kuwayama (Hemiptera: Liviidae), transmits both *Ca.* L. americanus and *Ca.* L. asiaticus [1].

The nature of *Ca.* L. asiaticus–*D. citri* interactions extend from mutually beneficial to harmful [10]. For instance, infection with *Ca.* L. asiaticus benefited *D. citri* by enhancing its reproductive fitness [10]. On the other hand, *Ca.* L. asiaticus infection negatively affected the vector’s susceptibility to insecticides [11], increased *D. citri* propensity for dispersal [12], exploited the energy metabolism and *D. citri* defense responses [13,14], and shortened the lifespan and weakened the survival of the infected insects [10,15]. Interestingly, longevity (lifespan and survival) is strongly linked with numerous physiological issues, particularly the endogenous levels of melatonin. Our previous study showed that the reduced longevity of the infected psyllids was associated with lower endogenous melatonin content [15].

The indole-like compound, melatonin (*N*-acetyl-5-methoxytryptamine), is a tryptophan-derivative that is secreted by the pineal gland and ubiquitously distributed in various phylogenetically distant taxa including both prokaryotes and eukaryotes [15,16,17,18]. In animals, melatonin plays pleiotropic regulatory roles in several biological and physiological functions in vertebrates, invertebrates, and unicellular organisms. For example, it regulates sleep induction and circadian rhythm [19], oxidative stress [20], immune response and anti-inflammatory activities [21], cell death and aging [22], and apoptosis and autophagy [23,24]. In addition, it might have potential anti-cancer [25,26] and anti-tumor [27] roles.

Melatonin is synthesized exclusively from the amino acid _L_-tryptophan via the shikimic acid pathway [28]. Launching with _L_-tryptophan, melatonin is synthesized via four well-confirmed enzymatic steps in all living organisms [28,29]. In animals, _L_-tryptophan is initially hydroxylated to 5-hydroxytryptophan using tryptophan hydroxylase (TPH; EC 1.14.16.4), then it is decarboxylated to form the key intermediate, serotonin (also known as 5-hydroxytryptamine), using aromatic amino acid decarboxylase (AADC; EC 4.1.1.28) [28]. Subsequently, serotonin is converted to melatonin via two consecutive enzymatic steps [29] using serotonin *N*-acetyltransferase (SNAT; also known as arylalkylamine N-acetyltransferase (AANAT); EC 2.3.1.87) and acetylserotonin *O*-methyltransferase (ASMT; formerly known as hydroxyindole-*O*-methyltransferase (HIOMT); EC 2.1.1.4) [28,30,31,32] via two potential pathways that are shared by both plants and animals. 

In the first route, the penultima enzyme, SNAT, converts serotonin to *N*-acetylserotonin, while ASMT, the last enzyme, catalyzes *N*-acetylserotonin to form the final product, melatonin [28,30,32]. Alternatively, serotonin could be methylated to 5-methoxytryptamine using ASMT, then N-acetylated to produce melatonin using SNAT [31,33]. As mentioned above, both alternative routes for the biosynthesis of melatonin from serotonin are possibly occurring in both plants and animals and even microorganisms [28,29]. Nevertheless, distinct homologs of SNAT and ASMT have been reported between plants and animals, indicating their diverse origins during evolution [28,34]. 

Interestingly, most, if not all, of these genes were cloned and well-characterized previously in several plant species including *Arabidopsis thaliana* [31,35], *Oryza sativa* [32,36], *Vitis vinifera* [37,38], and the gymnosperm *Pinus taeda* [39]. Moreover, several melatonin biosynthesis-related genes have been cloned and characterized from numerous animal species including human [40,41], rats [42], guinea pig [43], chick [44], zebrafish [45], Atlantic croaker [46], and sheep [47]. Likewise, melatonin biosynthesis genes have been cloned and identified from the insect model, *Drosophila melanogaster*, including *TPH* (also known as tryptophan 5-hydroxylase (*T5H*)) [48,49], *AADC* (also known as dopa decarboxylase (*DDC*)) [50,51,52], and *AANAT* [53,54,55]. However, to the best of our knowledge, none of these enzymes have been previously identified from the Asian citrus psyllid, *D. citri.*

We believe that unguided functional genomic experiments are laborious and resource-intensive. The computational approaches can perform predictions with a variety of purposes which help to speed up the process in a more cost-efficient, and faster but more accurate manner. Our objectives for this study were to computationally identify the melatonin biosynthesis-related genes (*TPH*/*T5H*, *AADC*, *SNAT*/*AANAT*, and *ASMT*) in *D. citri*. Therefore, we employed comprehensive in silico and bioinformatics methods to (I) dig for melatonin biosynthesis-related genes homologies, (II) functionally analyze and predict active domains and important sites of these proteins, (III) model their three-dimensional structures, and (IV) investigate the ligand-receptor binding profile when possible. Furthermore, we evaluated the expression patterns of the predicted genes within the adults of *D. citri* after challenging with gradient infection rates of the phytopathogenic bacterium *Ca.* L. asiaticus and after the treatment with exogenous melatonin. Our findings of this study are a further step for optimization and cloning of melatonin biosynthesis genes of *D. citri*. 

## 2. Materials and Methods

### 2.1. In Silico Analysis

#### 2.1.1. Protein–Protein BLAST (BLASTp) Analysis

The Basic Local Alignment Search tool (BLAST), particularly the Protein–Protein BLAST (BLASTp) algorithm, was used to dig and identify sequences of *D. citri* that resemble the query amino acid sequence of known melatonin biosynthesis-related enzymes of model insects. Briefly, the protein sequence of tryptophan hydroxylase (*DmTPH*, also known as *DmT5H*; GenBank Accession no. NP_612080.1) [48,49], dopa decarboxylase, isoform B (DmDDC, also known as *DmAADC*; GenBank Accession no. NP_724164.1) [50,51,52], arylalkylamine N-acetyltransferase 1, isoform A (*DmAANAT*1, also known as *DmSNAT*; GenBank Accession no. NP_523839.2) [53,54,55] from the fruit fly (*Drosophila melanogaster*), and N-acetylserotonin O-methyltransferase-like protein, isoform X1 (*ClASMT*; GenBank Accession no. XP_014251646.1) from bed bug (*Cimex lectularius*) were matched with their homologous genes from *D. citri* using the Protein–Protein BLAST (BLASTP 2.8.0+) [56,57], based on recent available data on the two major databases included the “*Diaphorina citri* OGS v2.0 CDS” and “*Diaphorina citri* OGS v2.0 proteins” BLAST datasets available on the Citrus Greening Solutions website (https://citrusgreening.org/organism/Diaphorina_citri/genome, 12 February 2021) [58] and the most recent available data in GenBank, the national center for biotechnology information website (NCBI, http://www.ncbi.nlm.nih.gov/gene/, 12 February 2021), using the compositionally adjusted substitution matrices [57]. Moreover, the Nucleotide–Nucleotide BLAST (BLASTn) algorithm was used to retrieve the nucleotide sequence for the top-matched sequences producing significant alignments with known melatonin-biosynthetic genes. Subsequently, a shortlist of top-matches was generated (Table 1) based on the phylogenetic trees, identity more than 50% (except for *DcAANAT*s), and excluding all the hypothetical and low-quality proteins that have these characteristics.

#### 2.1.2. Evolutionary Analysis by Maximum Likelihood Method

The evolutionary history of all matched sequences for each gene was inferred using the maximum likelihood method and JTT matrix-based model [60]. Initial tree(s) for the heuristic search were obtained automatically by applying Neighbor-Join and BioNJ algorithms to a matrix of pairwise distances estimated using the JTT model, and then selecting the topology with a superior log-likelihood value. Evolutionary analyses were conducted in MEGA X [61]. 

#### 2.1.3. Multiple Sequence Alignment Analysis

Amino acid sequences from *D. citri* that produced significant alignments with known melatonin biosynthesis-related genes were simultaneously aligned using the Constraint-Based Alignment tool (COBALT; https://www.ncbi.nlm.nih.gov/tools/cobalt/re_cobalt.cgi, 12 February 2021) for multiple protein sequences [62]. Moreover, the top-matched sequences (amino acid and nucleotide sequences) of *D. citri* producing significant alignments with known melatonin-biosynthetic genes were used to generate the multiple sequence alignment by ClustalW (http://www.genome.jp/tools-bin/clustalw, 12 February 2021) [63], and the version 3.21 of BOXSHADE (https://embnet.vital-it.ch/software/BOX_form.html, 12 February 2021) was used to visualize conserved regions in the alignment. 

#### 2.1.4. Conserved Domains and Theoretical pI/Mw 

The protein sequences were interactively classified into families and identified functionally important domains and conserved sites using The InterPro tool (https://www.ebi.ac.uk/interpro/, 12 February 2021) [64]. Moreover, the theoretical isoelectric point (pI) and molecular weight (MW) were computed using the Compute pI/Mw tool (http://web.expasy.org/compute_pi, 12 February 2021) [65]. 

#### 2.1.5. Three-Dimensional (3D) Structure Modeling and RNA Secondary Structure

The SWISS-MODEL server (https://swissmodel.expasy.org/, 12 February 2021) [66], and the Protein Homology/Analogy Recognition Engine (Phyre2 Protein Fold Recognition Server, web portal-version 2.0; http://www.sbg.bio.ic.ac.uk/~phyre2/html/page.cgi?id=index, 12 February 2021) [67] were used for protein structure homology-modeling, structure-based function annotation, generating a three-dimensional (3D) structure, and prediction of membrane topology of the predicted sequences. The UCSF-Chimera package (version 1.15) (https://www.cgl.ucsf.edu/chimera/, 12 February 2021) [68] was used for interactive visualization of the predicted macromolecule (PDB format). Moreover, the RNAfold web server (http://rna.tbi.univie.ac.at/cgi-bin/RNAWebSuite/RNAfold.cgi, 12 February 2021) [69] was used to predict RNA secondary structures using DNA sequences. To directly compare the folding stability of the predicted RNA secondary structures of various sizes, the minimum free energy (MFE) was obtained and the normalized MFE was calculated by dividing the MFE by the number of nucleotides (bp).

### 2.2. Rearing of Healthy and Ca. L. asiaticus-Infected D. citri Colonies

Healthy colonies of *D. citri* were continuously reared in a secured growth room (27 ± 2 °C, 60 ± 5% relative humidity, and 16:8 h L/D photocycle) at Citrus Research and Education Center (CREC), University of Florida (28°10′ N, 81°71′ E), Lake Alfred, FL, USA. Insects were maintained on HLB-free alemow (*Citrus macrophylla*) trees. Random samples of *D. citri* adults and alemow leaves were collected monthly and tested for the presence of *Ca.* L. asiaticus using polymerase chain reaction (PCR) assay as described by Tatineni et al. [70] to ensure that colonies remained HLB-free. 

To obtain the *Ca.* L. asiaticus-infected colonies, *D. citri* from the healthy colonies were reared on HLB-symptomatic and PCR-positive *Ca.* L. asiaticus-infected alemow trees and maintained in a separate secured growth room, under the same conditions as described above, to avoid cross-contamination. To obtain gradient infection rates of *D. citri*, the infection rates were tested monthly and prior to each experiment using two different methods: (I) Testing the presence of *Ca*. L. asiaticus visually in 50 individual psyllids using binocular laboratory compound microscope; and (II) the infection rates with *Ca*. L. asiaticus were further confirmed by PCR as described by Tatineni et al. [70]. Based on these examinations, *D. citri* colonies were categorized into five infection rates (24, 34, 50, 58, and 70%), in addition to the healthy colony (0%), which were all tested in this study. The infection rates were consistent without significant differences between them throughout the whole study. Briefly, newly emerged adults (~2-days old) from each infection rate were collected using an aspirator without any sex-based discrimination.

### 2.3. Treatment with Exogenous Melatonin

Newly emerged *D. citri* adults (healthy versus *Ca.* L. asiaticus-infected (50% infection rate)) were caged in the feeding system described in our previous study [15] and were fed on 100 μL of 20% sucrose suspension as an artificial diet (mock control versus 500 μg mL^−1^ of melatonin in 20% sucrose solution). The artificial diet was placed between double-layered parafilm stretched over an acrylic feeding chamber that contained 50 adults (10 replicates/treatment). Insects were maintained at the same conditions as described above, and 72 h post-treatment (hpt), psyllids were collected using an aspirator for gene expression analysis.

### 2.4. Gene Expression Analysis Using Quantitative Real-Time PCR (RT-PCR)

Total RNA was extracted from five individual insects per replicate (10 replicates/treatment) using TriZol^®^ reagent (Ambion^®^, Life Technologies, New York, NY, USA), and the gene expression analysis was carried out as described in our previous study [15]. Quantification of transcript levels was used as a measure of the gene expression. Samples were analyzed in triplicate for each biological replicate. Primers for 6 melatonin biosynthesis-related genes (Table S1) were used to measure the gene expression. The relative expression of the consensus sequence among PCR products was determined according to the 2^−ΔΔ^*^C^*_T_ method [71]. For all gene expression experiments, data were normalized using two reference genes, α-Tubulin and actin, which previously showed high stability for transcript normalization in *D. citri* under biotic stress [14].

### 2.5. Statistical Analysis

All experiments were designed in a completely randomized design using 10 biological replicates per treatment. The analysis of variance technique (ANOVA) was used for statistical comparison between more than two treatments, followed by post-hoc pairwise comparisons using the Tukey–Kramer honestly significant difference test (Tukey HSD; *p* < 0.05). Additionally, a two-tailed *t*-test was used for statistical comparison between only two treatments (healthy versus infected) or (mock-treated versus melatonin-treated), and statistical significance was established as *p* < 0.05. Moreover, simple linear regression (SLR) analysis was performed to model the relationship between *Ca.* L. asiaticus infection rates (as an independent variable) and gene expression (as a dependent variable). The fitted regression line is expressed as a significant equation, as determined by the F test (*p* < 0.05). Both coefficients of determination (R^2^) and adjusted coefficient of determination (R^2^_adj_) were also obtained. Further, due to the observed nonlinear phenomena, data were fitted with a second-degree polynomial regression model (quadratic model) to understand the curvilinear relationship between *Ca.* L. asiaticus infection rates (as an independent variable) and gene expression (as a dependent variable). Polynomial regression models, the 95% confident curves for the estimated regression, quadratic equation, R^2^, R^2^_adj_, and *p*-value based on the F test (*p* < 0.05) were also obtained. JMP Statistical Software (SAS Institute, Cary, NC, USA) was used for all statistical analyses listed above.

## 3. Results

### 3.1. D. citri Genome Possesses a Putative Melatonin Biosynthetic Pathway

The predicted melatonin biosynthesis pathway in *D. citri* was dissected using a comparative in silico analysis. Putative candidate genes involved in the melatonin biosynthesis pathway were presented as the top-matched sequences producing significant alignments of melatonin-biosynthetic genes from model insects and were selected based on sequence similarity, the phylogenetic relationships with the query sequences, and based on the sequence identity between query sequences and predicted ones, after excluding all the hypothetical and low-quality proteins that have these characteristics (Table 1).

#### 3.1.1. *D. citri* Genome Encodes for two Putative Tryptophan 5-hydroxylase (DcT5H)

**Figure 1:**
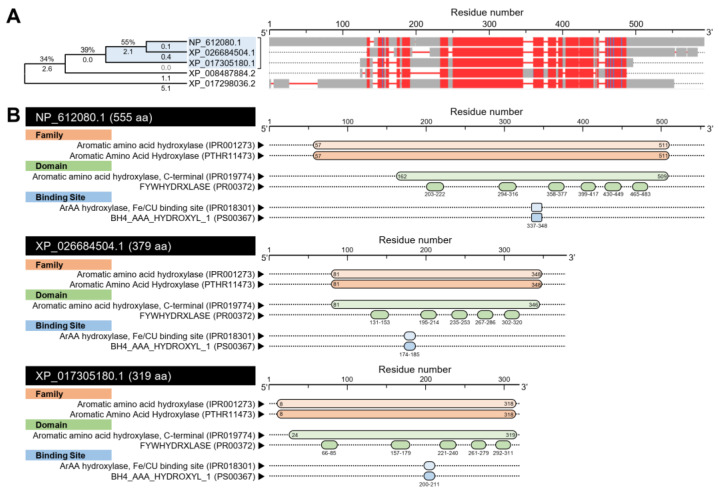
**In silico analysis of tryptophan 5-hydroxylase (*DcT5H*) of *Diaphorina citri*.** (**A**) Evolutionary analysis using maximum likelihood method and its associated multiple protein sequences alignments using Constraint-Based Alignment tool (COBALT) analysis. The AA sequences were identified using Protein–Protein BLAST (BLASTp) using tryptophan hydroxylase (*DmT5H*; GenBank Accession no. NP_612080.1) from *Drosophila melanogaster*, as a query sequence, against *Diaphorina citri* genome available in GenBank, the national center for biotechnology information website (NCBI, http://www.ncbi.nlm.nih.gov/gene/, 12 February 2021). The tree with the highest log likelihood (−5340.89) is shown. The tree is drawn to scale, with branch lengths measured in the number of substitutions per site (next to the branches). The proportion of sites where at least one unambiguous base is present in at least 1 sequence for each descendent clade is shown next to each internal node in the tree. Evolutionary analyses and the joint tree were conducted in MEGA-X software. In the COBALT analysis, residues were colored using a column-based method according to their relative entropy threshold. Aligned columns with no gaps are colored blue and red, where the red color indicates highly conserved columns and blue indicates less conserved ones. (**B**) The protein functional and conserved domains analysis of *DmT5H* (NP_612080.1), *DcT5H*-1 (XP_026684504.1), and *DcT5H*-2 (XP_017305180.1) using the InterPro Scan tool (https://www.ebi.ac.uk/interpro/, 12 February 2021). FYWHYDRXLASE: Biopterin-dependent aromatic amino acid hydroxylase signature; ArAA_hydroxylase_Fe/CU: Aromatic amino acid hydroxylase, iron/copper-binding site; and BH4_AAA_HYDROXYL_1: Non-heme iron and tetrahydrobiopterin (BH4)-dependent enzymes.

Using the Protein–Protein BLAST (BLASTp) tool, our findings showed that the *D. citri* genome possesses about six predicted amino acid sequences (based on NCBI database, Table S2) and nine sequences (based on *Diaphorina citri* OGS v2.0 proteins dataset, Table S3) that produce significant similarities to tryptophan hydroxylase (*DmT5H*, GenBank Accession no. NP_612080.1) from fruit fly (*D. melanogaster*). Although the multiple protein sequence alignment using COBALT analysis showed that all predicted sequences have relatively high homology with *DmT5H* protein, the phylogenetic analysis showed that only two proteins from *D. citri* were phylogenetically closer to the query sequence (approximately 55%) (Figure 1A). Those two proteins included putative tryptophan 5-hydroxylase 1-like (henceforth *DcT5H*-1) encoded by the *D. citri* locus LOC113470334 (GenBank Accession no. XP_026684504.1) and protein henna-like (henceforth *DcT5H*-2) by the *D. citri* locus LOC103524631 (GenBank Accession no. XP_017305180.1) (Figure 1A).

The NCBI protein sequences of *DcT5H*-1 and *DcT5H*-2 were aligned with the sequence of the top-matched proteins from the *D. citri* database (DcitrP076520.1.1 and DcitrP012845.1.1, respectively). The AA alignment showed high similarity and conserved sequences in both proteins (Figures S1 and S2). Furthermore, the nucleotide sequence of *DcT5H*-1 (GenBank Accession no. XM_026828703.1) and *DcT5H*-2 (GenBank Accession no. XM_017449691.2) had high similarity and conserved sequences when aligned with the mRNA sequences from the *D. citri* database (DcitrC076520.1.1 and DcitrC012845.1.1, respectively) (Figures S3 and S4). Collectively, these findings suggest sequences retrieved from the NCBI database were highly similar and homology with those of the *D. citri* database. Therefore, we focused on these proteins for further in silico analysis.

The bioinformatic analysis of amino acid sequences using the InterPro Scan tool to interactively predict the conserved domains suggests a high topological similarity between *DcT5H*s (*DcT5H*-1 and *DcT5H*-2) and *DmT5H* (Figure 1B). Briefly, all sequences have the tetrahydrobiopterin (BH4)-dependent aromatic amino acid (ArAA) hydroxylase family (IPR001273 and PTHR11473), and aromatic amino acid hydroxylase, C-terminal domain (IPR019774), FYWHYDRXLASE domain (PR00372), ArAA hydroxylase, Fe/CU binding site (IPR018301), and BH4_AAA_HYDROXYL_1 (PS00367) (Figure 1B).

**Figure 2:**
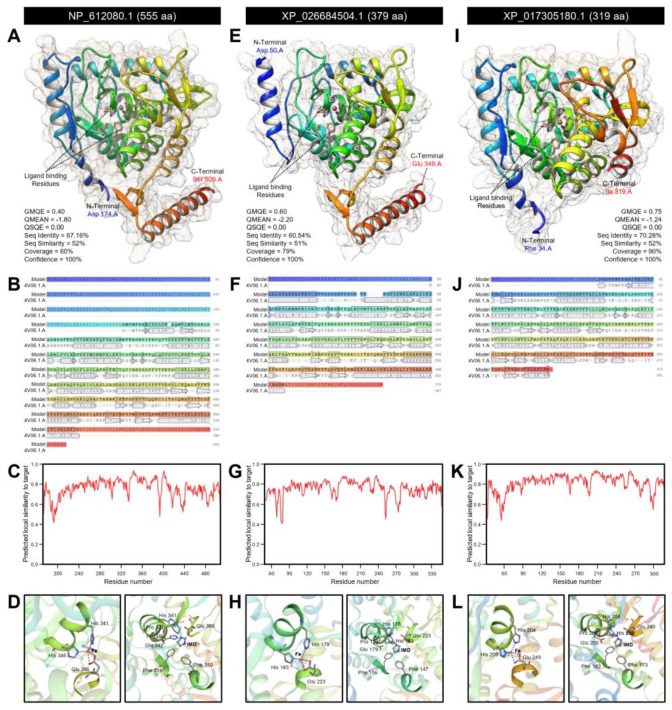
**The crystallographic three-dimensional (3D) modeling of tryptophan 5-hydroxylase (*DcT5H*) of *Diaphorina citri*.** (**A**,**E**,**I**) The predicted three-dimensional (3D) structure model and its associated mesh surface of *DmT5H* (NP_612080.1), *DcT5H*-1 (XP_026684504.1), and *DcT5H*-2 (XP_017305180.1), respectively. The tertiary structures were predicted with 100.0% confidence by the single highest scoring template of the crystal structure of human tryptophan 5-hydroxylase 2 (also known as tryptophan hydroxylase 2; TPH2), catalytic domain (Protein Data Bank (PDB ID): 4v06.1.A), and refined to 2.63 Å resolution. Protein chains are colored according to the rainbow color spectrum, from blue (N-terminus) to red (C-terminus). (**B**,**F**,**J**) Model–template alignment of *DmT5H*, *DcT5H*-1, and *DcT5H*-2, respectively. AA sequences of each model were aligned with the template (4v06.1.A). Secondary structures are represented by rectangles (*α*-helices) and arrows (*β*-sheets). Matched sequences are indicated in black. (**C**,**G**,**K**) Local quality estimate of the predicted models of *DmT5H*, *DcT5H*-1, and *DcT5H*-2, respectively. (**D**,**H**,**L**) Close-up of the ligand-binding site of *DmT5H*, *DcT5H*-1, and *DcT5H*-2, respectively. The selected poses were oriented to show the entry point for iron (Fe) and imidazole (IMD). Some surrounding AA near to the ligand are shown with their residue number. All bioinformatics analyses were carried out based on recent available data on the “*Diaphorina citri* OGS v2.0 proteins” dataset available on Citrus Greening Solutions website (https://citrusgreening.org/organism/Diaphorina_citri/genome, 12 February 2021) and the most recent available data in GenBank, the national center for biotechnology information website (NCBI, http://www.ncbi.nlm.nih.gov/gene/, 12 February 2021). The 3D structure was created using the SWISS-MODEL server (https://swissmodel.expasy.org/, 12 February 2021) and visualized with the UCSF-Chimera package (version 1.15) (https://www.cgl.ucsf.edu/chimera/, 12 February 2021). GMQE: Global model quality estimation and QSQE: Quaternary structure quality estimate.

The crystallographic three-dimensional (3D) structure of *DmT5H*, *DcT5H*-1, and *DcT5H*-2 was predicted using the crystal structure of human tryptophan 5-hydroxylase 2 (also known as tryptophan hydroxylase 2; TPH2), catalytic domain (Protein Data Bank (PDB ID): 4v06.1.A), and refined to 2.63 Å resolution with excellent statistics (Figure 2). 

Briefly, approximately 60% (residues Asp 174 to Ser 509) of *DmT5H* have been modeled with the template protein (seq identity = 67.16%, seq similarity = 52%, and confidence = 100%) with accepted global model quality estimation (GMQE = 0.40) and good absolute quality (QMEAN Z-score = −1.80) (Figure 2A). The predicted model of *DmT5H* is a monomer composed of 14 α-helices (three of them are short) and 14 β-sheets (Figure 2A,B) with considerable predicted local similarity to target (Figure 2C). Further, it has two ligand-binding sites for iron (Fe; residues His 341, His 346, and Glu 386) and imidazole (IMD; residues Phe 310, Phe 319, Pro 337, His 341, Glu 342, His 346, and Glu 386) (Figure 2D).

Similarly, about 79% (residues Asp 50 to Glu 349) of *DcT5H*-1 have been modeled with the target protein (seq identity = 60.54%, seq similarity = 51%, and confidence = 100%) with high GMQE and QMEAN (0.60 and −2.20, respectively) (Figure 2E). The predicted model of *DcT5H*-1 contains 13 α-helix ribbons and 11 stranded β-wings (Figure 2E,F) with considerable predicted local similarity to the target (Figure 2G). Comparable to *DmT5H*, *DcT5H*-1 has two ligand-binding sites for iron (Fe; residues His 178, His 183, and Glu 223) and imidazole (IMD; seven residues included Phe 147, Phe 156, Pro 174, His 178, Glu 179, His 183, and Glu 223) (Figure 2H). Likewise, about 90% (residues Phe 34 to Ile 319) of *DcT5H*-2 have been modeled with the target protein (seq identity = 70.28%, seq similarity = 52%, and confidence = 100%) with high GMQE (0.75) and QMEAN (−1.24) (Figure 2I). The predicted model of *DcT5H*-2 contains 14 α-helices (three were short) and 11 stranded β-sheets (Figure 2I,J) with considerable predicted local similarity to target (Figure 2K). Similar to *DmT5H* and *DcT5H*-1, *DcT5H*-2 had two ligand-binding sites for iron (Fe; residues His 204, His 209, and Glu 249) and imidazole (IMD; seven residues included Phe 173, Phe 182, Pro 200, His 204, Glu 205, His 209, and Glu 249) (Figure 2L).

**Figure 3:**
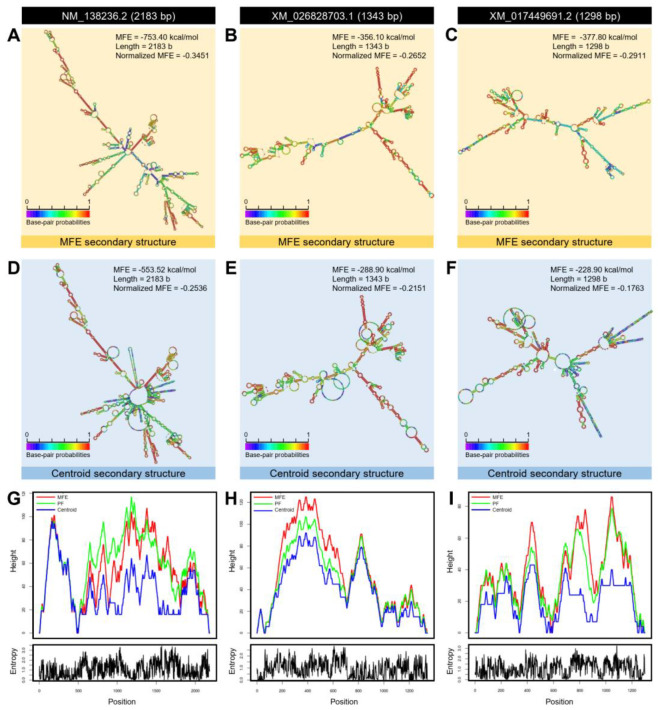
**mRNA hairpins of tryptophan 5-hydroxylase (*DcT5H*) of *Diaphorina citri***. (**A**–**C**) Predicted minimum free energy (MFE) secondary structure of *DmT5H* (NM_138236.2), *DcT5H*-1 (XM_026828703.1), and *DcT5H*-2 (XM_017449691.2), respectively. (**D**–**F**) Predicted centroid secondary structure of *DmT5H*, *DcT5H*-1, and *DcT5H*-2, respectively. Colors represent strengths with base-pairing probabilities. (**G**–**I**) The mountain plot representations of the MFE structure, the centroid structure, the thermodynamic ensemble of mRNA structures, and the positional entropy of *DmT5H*, *DcT5H*-1, and *DcT5H*-2, respectively. mRNA secondary structures were predicted using RNAfold web server (http://rna.tbi.univie.ac.at/cgi-bin/RNAWebSuite/RNAfold.cgi, 12 February 2021) using the nucleotide sequences.

Moreover, using the nucleotide sequence, we predicted the mRNA hairpins of *DmT5H* (NM_138236.2; 2183 bp), *DcT5H*-1 (XM_026828703.1; 1343 bp), and *DcT5H*-2 (XM_017449691.2; 1298 bp) with strengths of base pairing probabilities of minimum free energy (MFE; Figure 3A–C, respectively) and centroid secondary structures (Figure 3D–F, respectively). The results obtained with RNAfold analysis demonstrated that the mRNA hairpins of *DmT5H*, *DcT5H*-1, and *DcT5H*-2 could generate a stable MFE secondary structure (MFE = −753.40, −356.10, and −377.80 Kcal/mol, respectively) and centroid secondary structure (MFE = −553.52, −288.90, and −228.90 Kcal/mol, respectively). Furthermore, normalized MFE has been used for a direct comparison of the folding stability of predicted mRNA hairpins of various sizes. The MFE secondary structure of *DcT5H*-1 was more stable (normalized MFE = −0.2652; Figure 3B) than *DcT5H*-2 (normalized MFE = −0.2911; Figure 3C) and *DmT5H* (normalized MFE = −0.3451; Figure 3A). Whereas the centroid secondary structure of *DcT5H*-2 was more stable (normalized MFE = −0.1763; Figure 3F) than *DcT5H*-1 (normalized MFE = −0.2151; Figure 3E) and *DmT5H* (normalized MFE = −0.2536; Figure 3D). 

Additionally, the mountain plot representation of the MFE structure, the centroid structure, the thermodynamic ensemble of RNA structures, and the positional entropy of *DmT5H* (Figure 3G), *DcT5H*-1 (Figure 3H), and *DcT5H*-2 (Figure 3I) suggested that all predicted secondary RNA structures were thermodynamically stable with the superiority of *DcT5H*-1. Further, no significant disparity was observed among the predicted structures which can be considered as proof of the stability of the secondary structures.

#### 3.1.2. *D. citri* Genome Encodes for a Putative Aromatic Amino Acid Decarboxylase (DcAADC)

**Figure 4:**
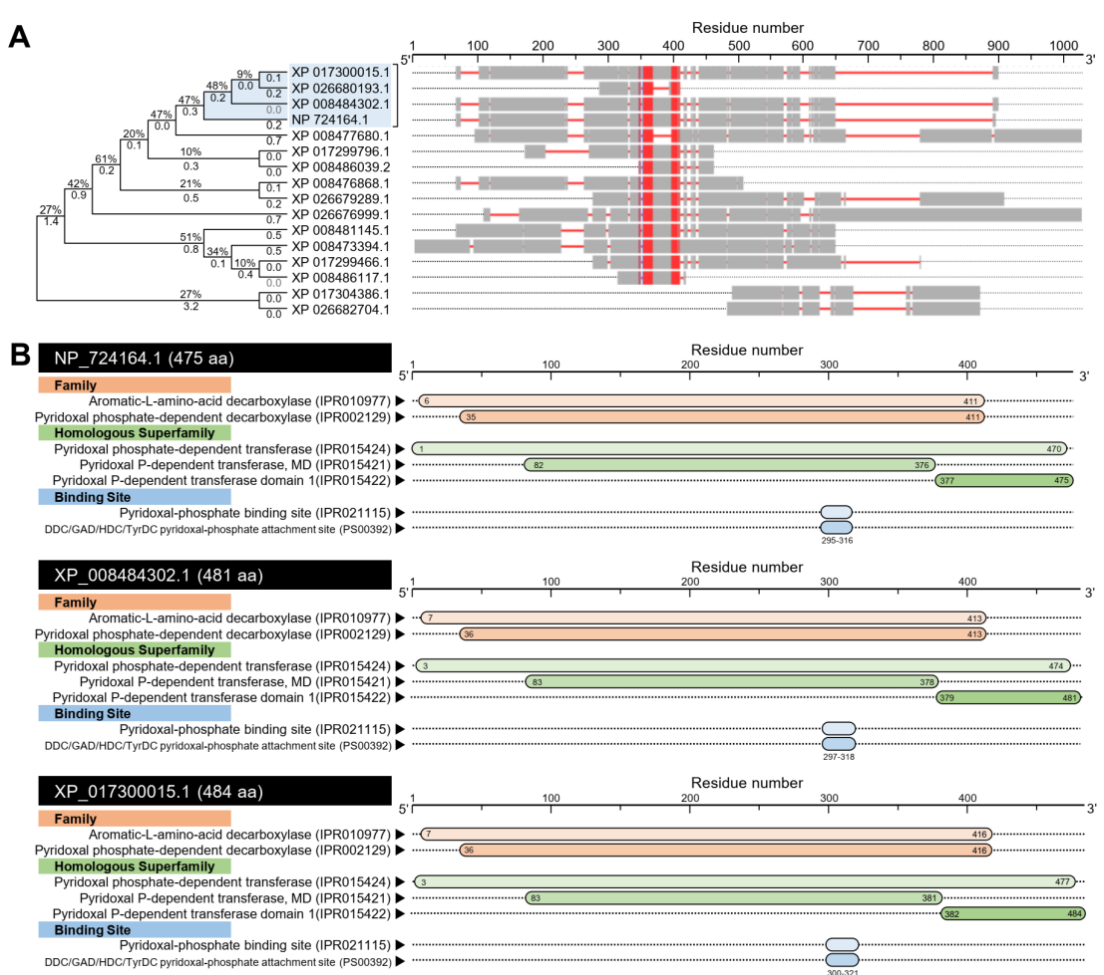
**In silico analysis of aromatic L-amino acid decarboxylase (*DcAADC*) of *Diaphorina citri*.** (**A**) Evolutionary analysis using maximum likelihood method and its associated multiple protein sequences alignments using Constraint-Based Alignment tool (COBALT) analysis. The AA sequences were identified using the Protein–Protein BLAST (BLASTp) using dopa decarboxylase, isoform B (GenBank Accession no. NP_724164.1) from *Drosophila melanogaster*, as a query sequence, against *Diaphorina citri* genome available in GenBank, the national center for biotechnology information website (NCBI, http://www.ncbi.nlm.nih.gov/gene/, 12 February 2021). The tree with the highest log likelihood (−11,911.42) is shown. The tree is drawn to scale, with branch lengths measured in the number of substitutions per site (next to the branches). The proportion of sites where at least one unambiguous base is present in at least one sequence for each descendent clade is shown next to each internal node in the tree. Evolutionary analyses and the joint tree were conducted in MEGA-X software. In the COBALT analysis, residues were colored using a column-based method according to their relative entropy threshold. Aligned columns with no gaps are colored blue and red, where the red color indicates highly conserved columns and blue indicates less conserved ones. (**B**) The protein functional and conserved domains analysis of *DmAADC* (NP_724164.1), *DcAADC*-1(XP_008484302.1), and *DcAADC*-2 (XP_017300015.1) using InterPro Scan tool (https://www.ebi.ac.uk/interpro/, 12 February 2021). Pyridoxal P-dependent transferase, MD: Pyridoxal phosphate-dependent transferase, major domain; DDC: Aromatic L-amino acid decarboxylase; GAD: Glutamate decarboxylase; HDC: Histidine decarboxylase; and TyrDC: Tyrosine decarboxylase.

In silico analysis using the BLASTp tool showed that the *D. citri* genome possesses about 15 sequences (based on NCBI database, Table S4) and 16 sequences (based on *Diaphorina citri* OGS v2.0 proteins dataset, Table S5) that produce significant similarities to dopa decarboxylase, isoform B (DmDDC, also known as aromatic L-amino acid decarboxylase (*DmAADC*); GenBank Accession no. NP_724164.1) from *D. melanogaster*. The multiple protein sequence alignment using COBALT analysis using the NCBI sequences showed that all predicted sequences have relatively high homology with DmDDC protein (Figure 4A). The phylogenetic analysis showed that only three proteins from *D. citri* were phylogenetically closer to the query sequence (Figure 4A). These proteins included aromatic L-amino acid decarboxylase, isoform X1 (henceforth *DcAADC*-1) encoded by the *D. citri* locus LOC103520978 (481aa; GenBank Accession no. XP_008484302.1), aromatic L-amino acid decarboxylase (henceforth *DcAADC*-2) encoded by the *D. citri* locus LOC103510318 (484; GenBank Accession no. XP_017300015.1), and aromatic L-amino acid decarboxylase-like encoded by the *D. citri* locus LOC103510317 (93 aa; GenBank Accession no. XP_026680193.1) (Figure 4A). However, the latest sequence was excluded from our further analysis because it was very short compared with the query sequence, and it had low query cover (14%; Table S4).

Interestingly, both protein sequences (*DcAADC*-1 and *DcAADC*-2) were highly similar to each other and showed conserved sequences to the top-matched protein from the *D. citri* database (DcitrP031955.1.1; 481 aa) (Figure S5). Likewise, the nucleotide sequence of *DcAADC*-1 (1939 bp, GenBank Accession no. XM_008486080.3) and *DcAADC*-2 (1737 bp, GenBank Accession no. XM_017444526.2) had high similarity and conserved sequences when aligned with the mRNA sequences from the *D. citri* database (1446 bp, DcitrC031955.1.1) (Figure S6). Together, these findings suggest both sequences retrieved from the NCBI database presented the same protein sequence from the *D. citri* database. 

The prediction of the conserved domains using the InterPro Scan tool suggests a high topological similarity among *DmAADC*, *DcAADC*-1, and *DcAADC*-2 (Figure 4B). All sequences had two families included aromatic L-amino acid decarboxylase (IPR010977) and pyridoxal phosphate-dependent decarboxylase (IPR002129); three homologous pyridoxal phosphate-dependent transferase superfamilies (IPR015424, IPR015421, and IPR015422); and two binding sites including the pyridoxal-phosphate binding site (IPR021115) and DDC/GAD/HDC/TyrDC pyridoxal-phosphate attachment site (PS00392) (Figure 4B).

**Figure 5:**
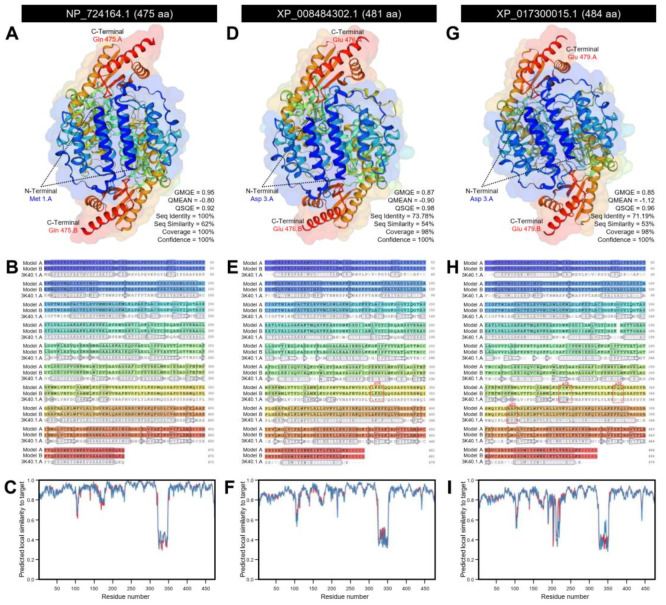
**The crystallographic three-dimensional (3D) modeling of aromatic L-amino acid decarboxylase (*DcAADC*) of *Diaphorina citri*.** (**A**,**D**,**G**) The predicted three-dimensional (3D) structure model and its associated surface of *DmAADC* (NP_724164.1), *DcAADC*-1(XP_008484302.1), and *DcAADC*-2 (XP_017300015.1), respectively. The tertiary structures were predicted with 100.0% confidence by the single highest scoring template of the crystal structure of *Drosophila* 3,4-dihydroxyphenylalanine decarboxylase (also known as aromatic L-amino acid decarboxylase; PDB ID 3k40.1.A) and refined to 1.75 Å resolution. Protein chains are colored according to the rainbow color spectrum, from blue (N-terminus) to red (C-terminus). (**B**,**E**,**H**) Model–template alignment of *DmAADC*, *DcAADC*-1, and *DcAADC*-2, respectively. AA sequences of each model were aligned with the template (3k40.1.A). Secondary structures are represented by rectangles (*α*-helices) and arrows (*β*-sheets). Matched sequences are indicated in black. (**C**,**F**,**I**) Local quality estimate of the predicted models of *DmAADC*, *DcAADC*-1, and *DcAADC*-2, respectively. All bioinformatics analyses were carried out based on recent available data on the “*Diaphorina citri* OGS v2.0 proteins” dataset available on Citrus Greening Solutions website (https://citrusgreening.org/organism/Diaphorina_citri/genome, 12 February 2021) and the most recent available data in GenBank, the national center for biotechnology information website (NCBI, http://www.ncbi.nlm.nih.gov/gene/, 12 February 2021). The 3D structure was created using the SWISS-MODEL server (https://swissmodel.expasy.org/, 12 February 2021) and visualized with the UCSF-Chimera package (version 1.15) (https://www.cgl.ucsf.edu/chimera/, 12 February 2021). GMQE: Global model quality estimation and QSQE: Quaternary structure quality estimate.

The crystallographic 3D structure of *DmAADC*, *DcAADC*-1, and *DcAADC*-2 was predicted using the crystal structure of *Drosophila* 3,4-dihydroxyphenylalanine decarboxylase (also known as aromatic L-amino acid decarboxylase; PDB ID 3k40.1.A) and refined to 1.75 Å resolution with excellent statistics (Figure 5). *DmAADC* was predicted as a homodimer composed of two identical units. Each unit represented a 100% coverage (residues Met 1 to Gln 475) with the template protein with remarkable statistics (GMQE = 0.95, QMEAN = −0.80, QSQE = 0.92, seq identity = 100%, seq similarity = 62%, and confidence = 100%) (Figure 5A). Further, each unit was composed of 23 α-helices, 11 long β-sheets, and six short β-sheets (Figure 5A,B) with high predicted local similarity to target (Figure 5C). 

Likewise, *DcAADC*-1 and *DcAADC*-2 were also predicted as homodimers that combined two units (model A and model B) with slight differences between both units in each model (Figure 5). Briefly, the predicted model of *DcAADC*-1 covered 98% with the target protein (residues Asp 3 to Glu 476) with notable statistics (GMQE = 0.87, QMEAN = −0.90, QSQE = 0.98, seq identity = 73.78%, seq similarity = 54%, and confidence = 100%) and were composed of 21 α-helix ribbons for model A or 22 α-helices for model B and 17 stranded β-sheets for each model (six of them were short) (Figure 5D,E, respectively) with considerable predicted local similarity to target (Figure 5F). The extra α-helix of model B (α18) was represented by four residues including Leu 334, Lys 335, His 336, and Asp 337 (Figure 5E).

Similarly, in each unit of *DcAADC*-2, approximately 98% of the protein sequence (residues Asp 3 to Glu 479) have been modeled with the target protein with significant statistics (GMQE = 0.85, QMEAN = −1.12, QSQE = 0.96, seq identity = 71.19%, seq similarity = 53%, and confidence = 100%) (Figure 5G). Model A of *DcAADC*-2 had 23 α-helix ribbons, whereas model B had only 20 α-helices, and both models had 15 β-sheets each (four of them were short) (Figure 5G,H) with high predicted local similarity to target (Figure 5I). Model A of *DcAADC*-2 had three extra α-helix ribbons (α18, α19, and α20, respectively). The ribbon α18 was composed of four residues (Pro 323, Ser 324, Trp 325, and Val 326), α19 composed of three residues (Asp 340, Gln 341, and Gln 342), and α20 composed of three residues also (Arg 357, Arg 358, and Phe 359) (Figure 5H).

**Figure 6:**
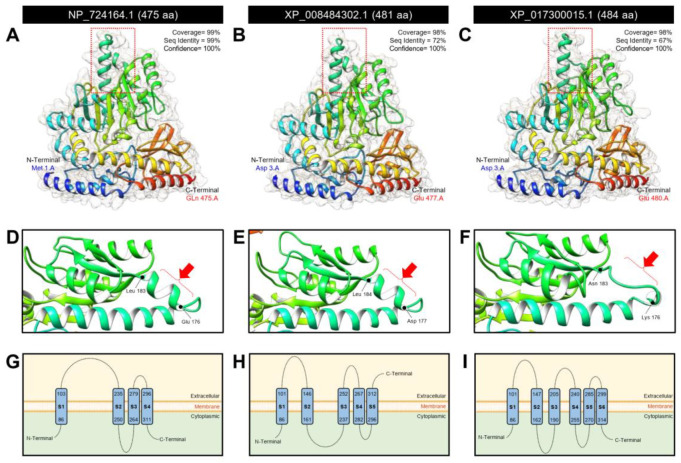
**The predicted topology of aromatic L-amino acid decarboxylase (*DcAADC*) of *Diaphorina citri*.** (**A**–**C**) The predicted three-dimensional (3D) monomer structures and their associated mesh surface of *DmAADC* (NP_724164.1), *DcAADC*-1(XP_008484302.1), and *DcAADC*-2 (XP_017300015.1), respectively. (**D**–**F**) Close-up of the area that outlined with red-dotted lines in panels A, B, and C, respectively. The selected poses were oriented to show one *α*-helix ribbon that was present in *DmAADC*, *DcAADC*-1, but it was absent in *DcAADC*-2 (pointed by red arrows). Some surrounding AA are shown with their residue number. (**G**–**I**) Schematic representation of Phyre2-based predicted topology of *DmAADC*, *DcAADC*-1, and *DcAADC*-2, respectively. The extracellular and cytoplasmic sides of the membrane are labeled, and the beginning and end of each transmembrane helix illustrated with a number indicating the residue index. Numbers inside the transmembrane (TM) domains (blue rectangle) denote amino acid residues. The predicted topology of TMs have been predicted using the protein homology/analogy recognition engine (Phyre2 web portal-version 2.0) (http://www.sbg.bio.ic.ac.uk/~phyre2/html/page.cgi?id=index, 12 February 2021).

Furthermore, the structure and/or function of the three protein sequences (*DmAADC*, *DcAADC*-1, and *DcAADC*-2) were deeply analyzed using the Phyre2 protein fold recognition server (Figure 6). The crystallographic 3D structures of the three proteins were predicted using the same protein template (PDB ID 3k40.1.A). All 3D structures were predicted as monomers and they were almost identical (Figure 6A–C) except for only one α-helix ribbon that was present in *DmAADC*, *DcAADC*-1 (Figure 6D,E, respectively), but it was absent in *DcAADC*-2 (Figure 6F). Additionally, Phyre2-based predicted topology suggested that the three predicted proteins (*DmAADC*, *DcAADC*-1, and *DcAADC*-2) might act as transporters. The predicted topology of *DmAADC* showed that it contains four transmembrane domains (S1–S4) (three connecting loops, and internal N- and C-termini (Figure 6G)) *DcAADC*-1 contains five transmembrane domains (S1–S5) (four connecting loops, internal N-terminal, and external C- terminal (Figure 6H)), and *DcAADC*-2 contains six transmembrane domains (S1–S6) (five connecting loops, and internal N- and C-terminal extremities (Figure 6I)).

**Figure 7:**
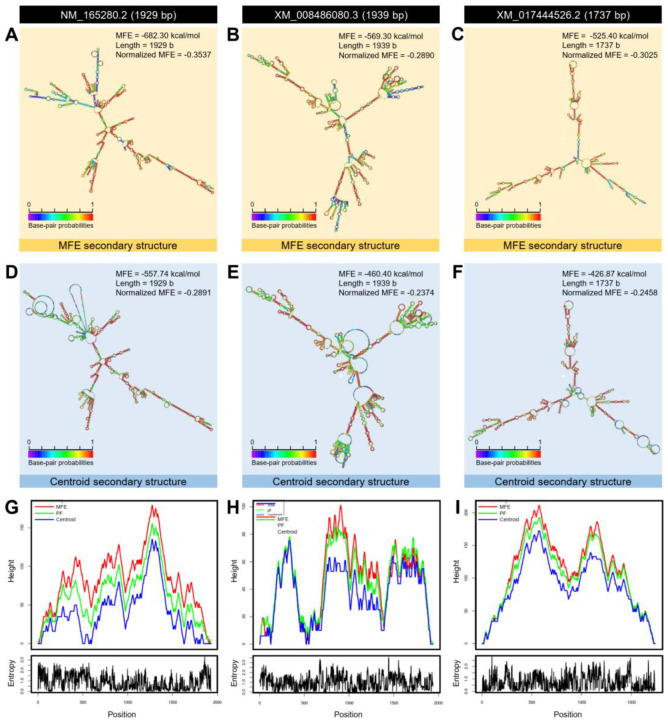
**mRNA hairpins of aromatic L-amino acid decarboxylase (*DcAADC*) of *Diaphorina citri*.** (**A**–**C**) Predicted minimum free energy (MFE) secondary structure of *DmAADC* (NM_165280.2), *DcAADC*-1 (XM_008486080.3), and *DcAADC*-2 (XM_017444526.2), respectively. (**D**–**F**) Predicted centroid secondary structure of *DmAADC*, *DcAADC*-1, and *DcAADC*-2, respectively. Colors represent strengths with base-pairing probabilities. (**G**–**I**) The mountain plot representations of the MFE structure, the centroid structure, the thermodynamic ensemble of mRNA structures, and the positional entropy of *DmAADC*, *DcAADC*-1, and *DcAADC*-2, respectively. mRNA secondary structures were predicted using RNAfold web server (http://rna.tbi.univie.ac.at/cgi-bin/RNAWebSuite/RNAfold.cgi, 12 February 2021) using the nucleotide sequences.

Additionally, the mRNA hairpins of *DmAADC* (NM_165280.2; 1929 bp), *DcAADC*-1 (XM_008486080.3; 1939 bp), and *DcAADC*-2 (XM_017444526.2; 1737 bp) were predicted using the nucleotide sequence with strengths of base pairing probabilities of minimum free energy (MFE; Figure 7A–C, respectively) and centroid secondary structures (Figure 7D–F, respectively). The RNAfold analysis demonstrated that the mRNA hairpins of *DmAADC*, *DcAADC*-1, and *DcAADC*-2 could produce stable MFE secondary structures (MFE = −682.30, −569.30, and −525.40 Kcal/mol, respectively) and also stable centroid secondary structures (MFE = −557.74, −460.40, and −426.87 Kcal/mol, respectively). Due to the various sequences size, normalized MFE was calculated. The mRNA hairpin of *DcAADC*-1 was more stable than other sequences at its MFE (normalized MFE = −0.2890; Figure 7B) and centroid (normalized MFE = −0.2374; Figure 7E) secondary structures. Moreover, the mountain plot representation of the MFE structure, the centroid structure, the thermodynamic ensemble of RNA structures, and the positional entropy of *DmAADC* (Figure 7G), *DcAADC*-1 (Figure 7H), and *DcAADC*-2 (Figure 7I) suggested that all predicted secondary RNA structures were thermodynamically stable. Further, no significant disparities were observed among all predicted mRNA hairpins which can be considered as proof of the stability of the secondary structures.

#### 3.1.3. *D. citri* Genome Possesses Two Putative Arylalkylamine N-acetyltransferase Genes (DcAANAT)

**Figure 8:**
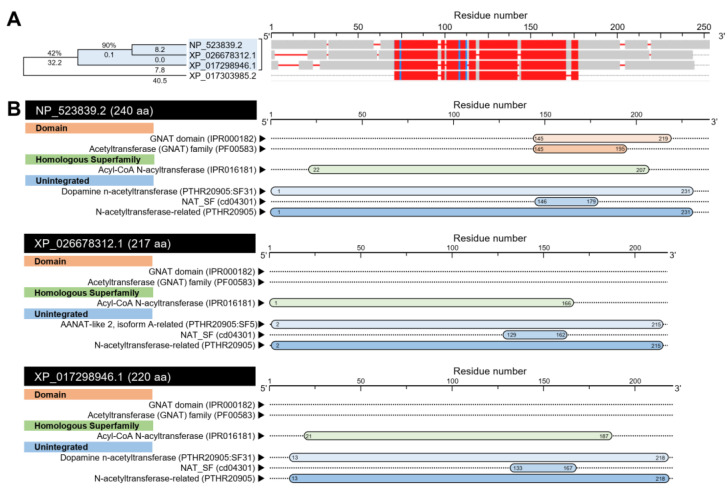
**In silico analysis of arylalkylamine N-acetyltransferase (*DcAANAT*) of *Diaphorina citri*.** (**A**) Evolutionary analysis using maximum likelihood method and its associated multiple protein sequences alignments using Constraint-Based Alignment tool (COBALT) analysis. The AA sequences were identified using the Protein–Protein BLAST (BLASTp) using arylalkylamine N-acetyltransferase 1, isoform A (*DmAANAT*; GenBank Accession no. NP_523839.2) from fruit fly, *Drosophila melanogaster*, as a query sequence, against the *Diaphorina citri* genome available in GenBank, the national center for biotechnology information website (NCBI, http://www.ncbi.nlm.nih.gov/gene/, 12 February 2021). The tree with the highest log likelihood (−2252.02) is shown. The tree is drawn to scale, with branch lengths measured in the number of substitutions per site (next to the branches). The proportion of sites where at least one unambiguous base is present in at least 1 sequence for each descendent clade is shown next to each internal node in the tree. Evolutionary analyses and the joint tree were conducted in MEGA-X software. In the COBALT analysis, residues were colored using a column-based method according to their relative entropy threshold. Aligned columns with no gaps are colored blue and red, where the red color indicates highly conserved columns and blue indicates less conserved ones. (**B**) The protein functional and conserved domains analysis of *DmAANAT* (NP_612080.1), *DcAANAT*-1 (XP_026678312.1), and *DcAANAT*-2 (XP_017298946.1) using InterPro Scan tool (https://www.ebi.ac.uk/interpro/, 12 February 2021). GNAT: GCN5-related N-acetyltransferases and NAT-SF: N-acyltransferase superfamily.

BLASTp analysis showed that *D. citri* genome encodes three AA sequences (based on NCBI database, Table S6) and only two protein sequences (based on *Diaphorina citri* OGS v2.0 proteins dataset, Table S7) with significant similarity to arylalkylamine N-acetyltransferase 1, isoform A (*DmAANAT*1, also known as DmSNAT; GenBank Accession no. NP_523839.2, 240 aa) from the fruit fly (*D. melanogaster*). The COBALT-based multiple protein sequences alignment showed that the predicted proteins had relatively high homology with *DmAANAT*1 protein; however, Dopamine N-acetyltransferase-like, isoform X1 (henceforth *DcAANAT*-1) encoded by the *D. citri* locus LOC103507708 (GenBank Accession no. XP_026678312.1; 217 aa) and dopamine N-acetyltransferase-like (henceforth *DcAANAT*-2) encoded by the *D. citri* locus LOC103507696 (GenBank Accession no. XP_017298946.1; 220 aa) were phylogenetically closer to *DmAANAT*1 (Figure 8A).

The NCBI protein sequences of *DcAANAT*-1 and *DcAANAT*-2 were separately aligned with the sequence of the top-matched proteins from the *D. citri* database (DcitrP025630.1.1; 217 aa and DcitrP085745.1.1; 240 aa, respectively). The AA alignment showed high similarity and conserved sequence in *DcAANAT*-1 (Figure S7) but not *DcAANAT*-2 (Figure S8). Furthermore, the nucleotide sequence of *DcAANAT*-1 (GenBank Accession no. XM_026822511.1; 1221 bp) had high similarity and conserved sequences when aligned with the mRNA sequence of DcitrC025630.1.1 (654 bp) from the *D. citri* database (Figure S9). Low similarity and conserved sequences were observed in *DcAANAT*-2 (GenBank Accession no. XM_017443457.2; 1911 bp) when aligned with the mRNA sequence of DcitrP085745.1.1; 240 aa from the *D. citri* database (Figure S10).

InterPro-based analysis of conserved domains suggests a comparable topological similarity between *DmAANAT*1, *DcAANAT*-1, and *DcAANAT*-2 (Figure 8B). All sequences had an acyl-CoA N-acyltransferase (IPR016181) homologous superfamily, and three unintegrated domains including dopamine N-acetyltransferase (PTHR20905:SF31), NAT_SF (cd04301), and N-acetyltransferase-related (PTHR20905) (Figure 8B). However, a Gcn5-related N-acetyltransferases (GNAT) domain (IPR000182) was predicted only in *DmAANAT*1 from *D. melanogaster*, but not in any *DcAANAT* genes from *D. citri* (Figure 8B).

**Figure 9:**
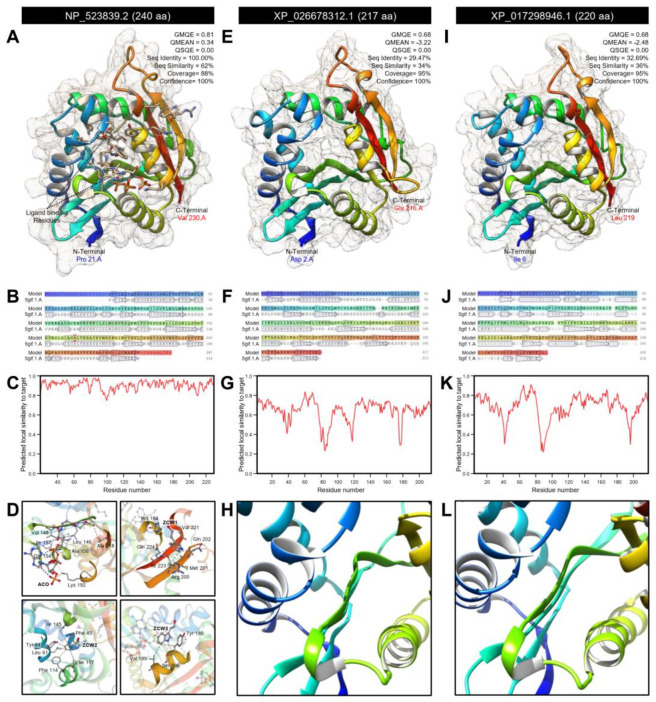
The crystallographic three-dimensional (3D) modeling of arylalkylamine N-acetyltransferase (*DcAANAT*) of *Diaphorina citri*. (**A**,**E**,**I**) The predicted three-dimensional (3D) structure model and its associated mesh surface of *DmAANAT* (NP_612080.1), *DcAANAT*-1 (XP_026678312.1), and *DcAANAT*-2 (XP_017298946.1), respectively. The tertiary structures were predicted with 100.0% confidence by the single highest scoring template of the crystal structure of *D. melanogaster* dopamine N-acetyltransferase in complex with CoA and tryptophol (PDB ID: 6k80.1.A) and refined to 1.28 Å resolution. Protein chains are colored according to the rainbow color spectrum, from blue (N-terminus) to red (C-terminus). (**B**,**F**,**J**) Model–template alignment of *DmAANAT*, *DcAANAT*-1, and *DcAANAT*-2, respectively. AA sequences of each model were aligned with the template (6k80.1.A). Secondary structures are represented by rectangles (*α*-helices) and arrows (*β*-sheets). Matched sequences are indicated in black. (**C**,**G**,**K**) Local quality estimate of the predicted models of *DmAANAT*, *DcAANAT*-1, and *DcAANAT*-2, respectively. (**D**,**H**,**L**) Close-up of the ligand-binding site of *DmAANAT*, *DcAANAT*-1, and *DcAANAT*-2, respectively. The selected poses were oriented to show the entry point for acetyl coenzyme A (ACO) and 2-(1H-indol-3-yl)ethanol (ZCW). Some surrounding AA near to the ligand are shown with their residue number. Gray dotted-lines indicate hydrophobic interactions, blue dotted-lines indicate hydrogen bonds, yellow dotted-lines indicate salt bridges, and green dotted-lines indicate pi-Stacking. All bioinformatics analyses were carried out based on recent available data on the “*Diaphorina citri* OGS v2.0 proteins” dataset available on Citrus Greening Solutions website (https://citrusgreening.org/organism/Diaphorina_citri/genome, 12 February 2021) and the most recent available data in GenBank, the national center for biotechnology information website (NCBI, http://www.ncbi.nlm.nih.gov/gene/, 12 February 2021). The 3D structure was created using the SWISS-MODEL server (https://swissmodel.expasy.org/, 12 February 2021) and visualized using the UCSF-Chimera package (version 1.15) (https://www.cgl.ucsf.edu/chimera/, 12 February 2021). GMQE: Global model quality estimation and QSQE: Quaternary structure quality estimate.

The protein tertiary structures of *DmAANAT*1, *DcAANAT*-1, and *DcAANAT*-2 were predicted using the crystal structure of *D. melanogaster* dopamine N-acetyltransferase in complex with CoA and tryptophol (PDB ID: 6k80.1.A) and refined to 1.28 Å resolution with accepted statistics (Figure 9). 

*DmAANAT*1 has been predicated as a monomer that covered approximately 88% of the template protein (GMQE = 0.81, QMEAN = 0.34, QSQE = 0.00, seq identity = 100.00%, seq similarity = 62%, and confidence = 100%) (Figure 9A). The predicted model was composed of six α-helices and ten β-sheets (Figure 9A,B) with a high local quality estimate (Figure 9C). Further, it had four ligand binding sites for one acetyl coenzyme A (ACO) and three 2-(1H-indol-3-yl)ethanol (ZCW) (Figure 9D). Briefly, 24 residues (Phe 43, Asp 46, Glu 47, Pro 48, Gly 143, Lys 144, Ile 145, Leu 146, Ser 147, Val 148, Arg 153, Gly 154, Leu 155, Gly 156, Ile 157, Ala 158, Val 179, Leu 180, Ser 182, Ser 186, Val 189, and Lys 192) within 4Å interacted with ACO to form 13 interactions including three hydrophobic interactions (at residues Leu 146, Val 148, and Val 179), eight hydrogen bonds (at residues Leu 146, Leu 146, Val 148, Gly154, Gly154, Gly156, Ile 157, and Ala 158), and two salt bridges with residue Lys 192 (Figure 9D).

Moreover, *DmAANAT*1 had three ZCW binding sites. ZCW 1 interacts with eight residues within 4Å including His 184, Arg 200, Met 201, Aln 202, Val 221, Gly 222, Ile 223, and Aln 224 to form four hydrophobic interactions at residues Arg 200, Aln 202, Val 221, and Aln 224 (Figure 9D). ZCW 2 interacts with 12 residues (Phe 43, Glu 47, Asn 50, Leu 61, Tyr 64, Phe 114, Ile 117, Met 121, Lys 144, Ile 145, Leu 180, and Ser 182) within 4Å to form five hydrophobic interactions (at residues Leu 61, Tyr 64, Phe 114, Ile 117, and Ile 145) and two pi-stacking with Phe 43 (Figure 9D). ZCW 2 interacts with only three residues (Tyr 185, Ser 186, and Val 189) within the same resolution (4Å) to form two hydrophobic interactions at Tyr 185 and Val 189 residues (Figure 9D).

Correspondingly, roughly 95% of the *DcAANAT*-1 sequence (residues Asp 2 to Glu 216) have been modeled with the target protein (GMQE = 0.68, QMEAN = −3.22, QSQE = 0.00, seq identity = 29.47%, seq similarity = 34%, and confidence = 100%) (Figure 9E). The predicted model of *DcAANAT*-1 contains eight α-helix ribbons and eight stranded β-wings (Figure 9E,F) with a high local quality estimate compared with the target template (Figure 9G). However, no ligand binding sites were observed (Figure 9H). Likewise, about 95% of *DcAANAT*-2 sequence (residues Ile 6 to Leu 219) were modeled with the target protein (GMQE = 0.68, QMEAN = −2.48, QSQE = 0.00, seq identity = 32.69%, seq similarity = 36%, and confidence = 100%) (Figure 9I). The predicted model of *DcAANAT*-2 contained seven α-helices and eight stranded β-sheets (Figure 9I,J) with substantial predicted local similarity to target (Figure 9K). Like *DcAANAT*-1, no ligand binding sites were observed in the model of *DcAANAT*-2 (Figure 9L).

**Figure 10:**
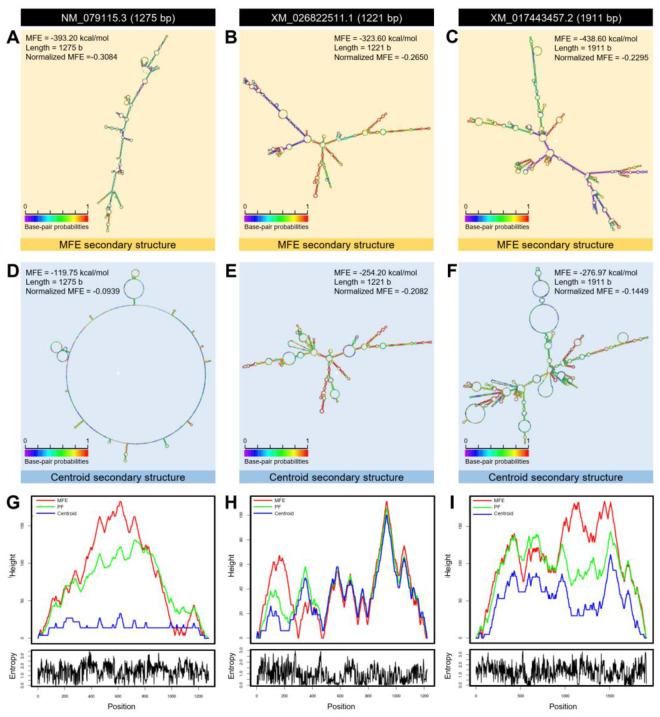
**mRNA hairpins of arylalkylamine N-acetyltransferase (*DcAANAT*) of *Diaphorina citri*.** (**A**–**C**) Predicted minimum free energy (MFE) secondary structure of *DmAANAT1* (NM_079115.3), *DcAANAT*-1 (XM_026822511.1), and *DcAANAT*-2 (XM_017443457.2), respectively. (**D**–**F**) Predicted centroid secondary structure of *DmAANAT*, *DcAANAT*-1, and *DcAANAT*-2, respectively. Colors represent strengths with base-pairing probabilities. (**G**–**I**) The mountain plot representations of the MFE structure, the centroid structure, the thermodynamic ensemble of mRNA structures, and the positional entropy of *DmAANAT*, *DcAANAT*-1, and *DcAANAT*-2, respectively. mRNA secondary structures were predicted using the RNAfold web server (http://rna.tbi.univie.ac.at/cgi-bin/RNAWebSuite/RNAfold.cgi, 12 February 2021) using the nucleotide sequences.

Furthermore, the mRNA hairpins of *DmAANAT*1 (NM_079115.3; 1275 bp), *DcAANAT*-1 (XM_026822511.1; 1221 bp) and *DcAANAT*-2 (XM_017443457.2; 1911 bp) were predicted with strengths of base pairing probabilities of MFE (Figure 10A–C, respectively) and centroid (Figure 10D–F, respectively) secondary structures. RNAfold analysis proved that although the mRNA hairpins of *DmAANAT*1, *DcAANAT*-1 and *DcAANAT*-2 could generate stable MFE secondary structures (MFE = −393.2, −323.6, and −438.6 Kcal/mol, respectively) and centroid secondary structures (MFE = −119.75, −254.2, and −276.97 Kcal/mol, respectively), the MFE and centroid secondary structures of *DcAANAT*-2 were more stable (normalized MFE = −0.2295 and −0.1449, respectively). Further, the thermodynamic ensemble and the positional entropy of *DmAANAT*1 (Figure 10G), *DcAANAT*-1 (Figure 10H), and *DcAANAT*-2 (Figure 10I) suggested that predicted mRNA hairpins were thermodynamically stable.

#### 3.1.4. *D. citri* Genome Encodes for a Putative N-acetylserotonin O-methyltransferase (DcASMT)

**Figure 11:**
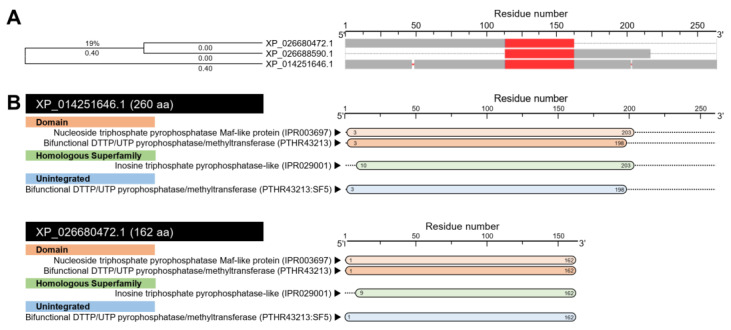
**In silico analysis of *N*-acetylserotonin O-methyltransferase (*DcASMT*) of *Diaphorina citri*.** (**A**) Evolutionary analysis using maximum likelihood method and its associated multiple protein sequences alignments using Constraint-Based Alignment tool (COBALT) analysis. The AA sequences were identified using the Protein–Protein BLAST (BLASTp) using *N*-acetylserotonin O-methyltransferase-like protein, isoform X1 (*ClASMT*; GenBank Accession no. XP_014251646.1) from bed bug (*Cimex lectularius*) as a query sequence, against *Diaphorina citri* genome available in GenBank, the national center for biotechnology information website (NCBI, http://www.ncbi.nlm.nih.gov/gene/, 12 February 2021). The tree is drawn to scale, with branch lengths measured in the number of substitutions per site (next to the branches). The proportion of sites where at least one unambiguous base is present in at least 1 sequence for each descendent clade is shown next to each internal node in the tree. Evolutionary analyses and the joint tree were conducted in MEGA-X software. In the COBALT analysis, residues were colored using a column-based method according to their relative entropy threshold. Aligned columns with no gaps are colored blue and red, where the red color indicates highly conserved columns and blue indicates less conserved ones. (**B**) The protein functional and conserved domains analysis of *ClASMT* (XP_014251646.1) and *DcASMT* (XP_026680472.1) using the InterPro Scan tool (https://www.ebi.ac.uk/interpro/, 12 February 2021).

Digging the *D. citri* genome using the BLASTp tool retrieved two sequences from the NCBI database (Table S8) and only one sequence from the *Diaphorina citri* OGS v2.0 proteins dataset (Table S9) that produced significant similarities to N-acetylserotonin O-methyltransferase-like protein, isoform X1 (*ClASMT*; 260 aa; GenBank Accession no. XP_014251646.1) from bed bug (*Cimex lectularius*). The multiple protein sequence alignment using COBALT analysis showed that all predicted sequences have relatively high homology with *ClASMT* protein (Figure 11A). One of these sequences (103 aa; GenBank Accession no. XP_026688590.1) was excluded because it was a partial sequence. However, septum formation protein Maf-like encoded by the *D. citri* locus LOC113468045 (162 aa; GenBank Accession no. XP_026680472.1) was used for further analysis. Interestingly, this AA sequence was highly similar and showed conserved sequences to N-acetylserotonin O-methyltransferase-like protein from the *D. citri* database (henceforth *DcASMT*; 274 aa; DcitrP032285.1.1) (Figure S11). Likewise, the nucleotide sequence of *DcASMT* (746 bp, GenBank Accession no. XM_026824671.1) had high similarity and conserved sequences when aligned with the mRNA sequences from the *D. citri* database (825 bp, DcitrC032285.1.1) (Figure S12). Together, these findings suggest that the *DcASMT* sequence retrieved from the NCBI database presented the same protein sequence from the *D. citri* database.

The prediction of the conserved domains using the InterPro Scan tool suggests a high topological similarity among *ClASMT* and *DcASMT* (Figure 11B). Both sequences had two domains included nucleoside triphosphate pyrophosphatase Maf-like protein (IPR003697) and bifunctional DTTP/UTP pyrophosphatase/methyltransferase (PTHR43213); a homologous inosine triphosphate pyrophosphatase-like superfamily (IPR029001); and an unintegrated bifunctional DTTP/UTP pyrophosphatase/methyltransferase (PTHR43213:SF5) domain (Figure 11B). 

**Figure 12:**
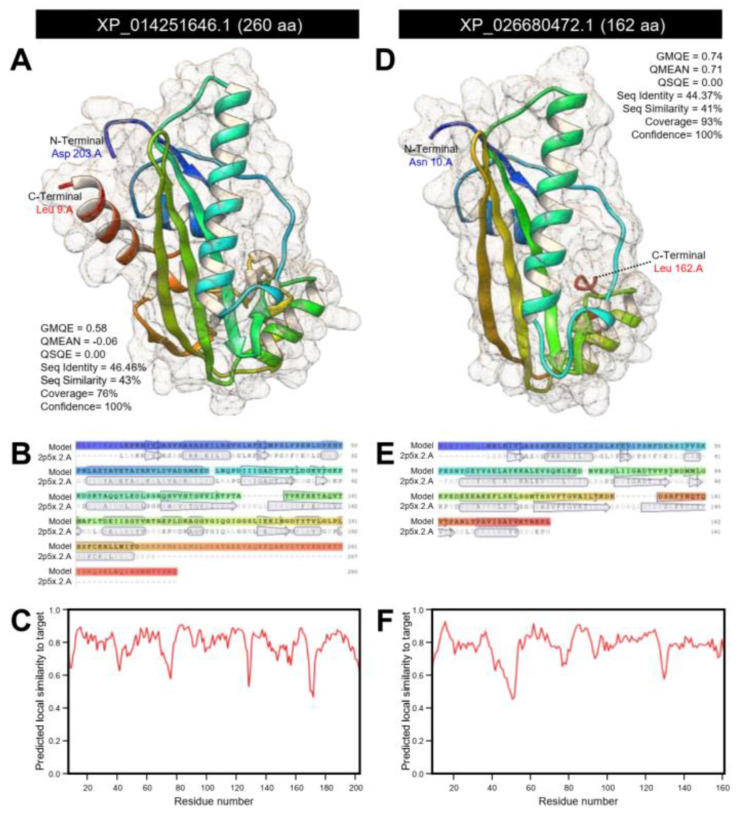
The crystallographic three-dimensional (3D) modeling of *N*-acetylserotonin O-methyltransferase (*DcASMT*) of *Diaphorina citri*. (**A**,**D**) The predicted three-dimensional (3D) structure model and its associated mesh surface of *ClASMT* (XP_014251646.1) and *DcASMT* (XP_026680472.1), respectively. The tertiary structures were predicted with 100.0% confidence by the single highest scoring template of the crystal structure of the Maf domain of human N-acetylserotonin O-methyltransferase-like protein (PDB ID: 2p5x.2.A) and refined to 2.00 Å resolution. Protein chains are colored according to the rainbow color spectrum, from blue (N-terminus) to red (C-terminus). (**B**,**E**) Model–template alignment of *ClASMT* and *DcASMT*, respectively. AA sequences of each model were aligned with the template (6k80.1.A). Secondary structures are represented by rectangles (*α*-helices) and arrows (*β*-sheets). Matched sequences are indicated in black. (**C**,**F**) Local quality estimate of the predicted models of *ClASMT* and *DcASMT*, respectively. All bioinformatics analyses were carried out based on recent available data on the “*Diaphorina citri* OGS v2.0 proteins” dataset available on Citrus Greening Solutions website (https://citrusgreening.org/organism/Diaphorina_citri/genome, 12 February 2021) and the most recent available data in GenBank, the national center for biotechnology information website (NCBI, http://www.ncbi.nlm.nih.gov/gene/, 12 February 2021). The 3D structure was created using the SWISS-MODEL server (https://swissmodel.expasy.org/, 12 February 2021) and visualized with the UCSF-Chimera package (version 1.15) (https://www.cgl.ucsf.edu/chimera/, 12 February 2021). GMQE: Global model quality estimation and QSQE: Quaternary structure quality estimate.

The crystallographic 3D structures of *ClASMT* and *DcASMT* were predicted using the crystal structure of the Maf domain of human N-acetylserotonin O-methyltransferase-like protein (PDB ID: 2p5x.2.A) and refined to 2.00 Å resolution with good statistics (Figure 12). *ClASMT* was predicted as a monomer that represented approximately 76% coverage (residues Leu 9 to Asp 203) with remarkable statistics (GMQE = 0.58, QMEAN = −0.06, QSQE = 0.00, seq identity = 46.46%, seq similarity = 43%, and confidence = 100%) (Figure 12A). The predicted model of *ClASMT* composed of nine α-helices and 11 β-sheets (Figure 12A,B) with good local quality estimate (Figure 12C). Likewise, about 93% of *DcASMT* sequence (residues Asn 10 to Leu 162) have been modeled with the target protein (GMQE = 0.74, QMEAN = 0.71, QSQE = 0.00, seq identity = 44.37%, seq similarity = 41%, and confidence = 100%) (Figure 12D). The predicted model of *DcASMT* contains four α-helices and seven stranded β-sheets (Figure 12D,E) with substantial predicted local similarity to target (Figure 12F).

**Figure 13:**
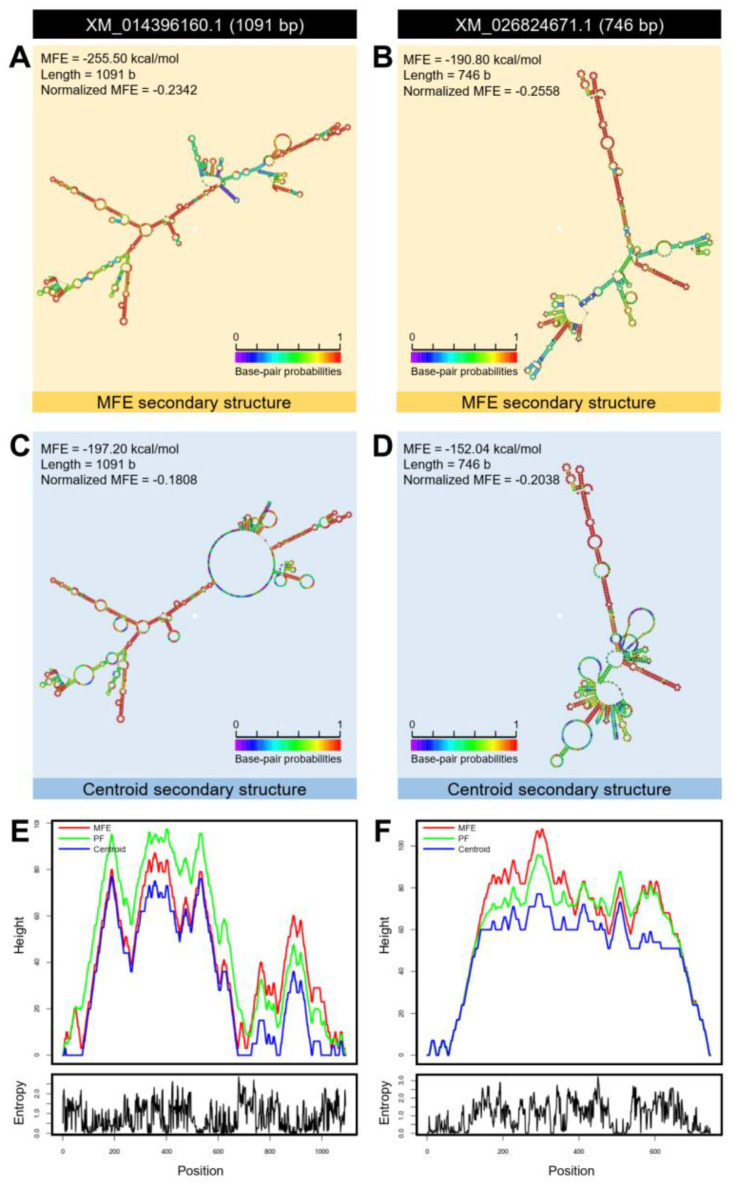
**mRNA hairpins of *N*-acetylserotonin O-methyltransferase (*DcASMT*) of *Diaphorina citri*.** (**A**,**B**) Predicted minimum free energy (MFE) secondary structure of *ClASMT* (XM_014396160.1) and *DcASMT* (XM_026824671.1), respectively. (**C**,**D**) Predicted centroid secondary structure of *ClASMT* and *DcASMT*, respectively. Colors represent strengths with base-pairing probabilities. (**E**,**F**) The mountain plot representations of the MFE structure, the centroid structure, the thermodynamic ensemble of mRNA structures, and the positional entropy of *ClASMT* and *DcASMT*, respectively. mRNA secondary structures were predicted using RNAfold web server (http://rna.tbi.univie.ac.at/cgi-bin/RNAWebSuite/RNAfold.cgi, 12 February 2021) using the nucleotide sequences.

Additionally, the mRNA hairpins of *ClASMT* (1091 bp; XM_014396160.1) and *DcASMT* (746 bp; GenBank Accession no. XM_026824671.1) were predicted using the nucleotide sequence with strengths of base pairing probabilities of minimum free energy (MFE; Figure 13A,B, respectively) and centroid secondary structures (Figure 13C,D, respectively). The RNAfold analysis demonstrated that the mRNA hairpins of *DmASMT* and *DcASMT* could produce stable MFE secondary structures (MFE = −255.50 and −190.80 Kcal/mol, respectively) and also stable centroid secondary structures (MFE = −197.20 and −152.04 Kcal/mol, respectively). Moreover, the mountain plot representation of the MFE structure, the centroid structure, the thermodynamic ensemble of RNA structures, and the positional entropy of *DmASMT* (Figure 13E) and *DcASMT* (Figure 13F) suggested that both predicted secondary RNA structures were thermodynamically stable. 

### 3.2. Ca. L. asiaticus Infection Downregulated the Expression of Melatonin Biosynthesis-Related Genes of D. citri

**Figure 14:**
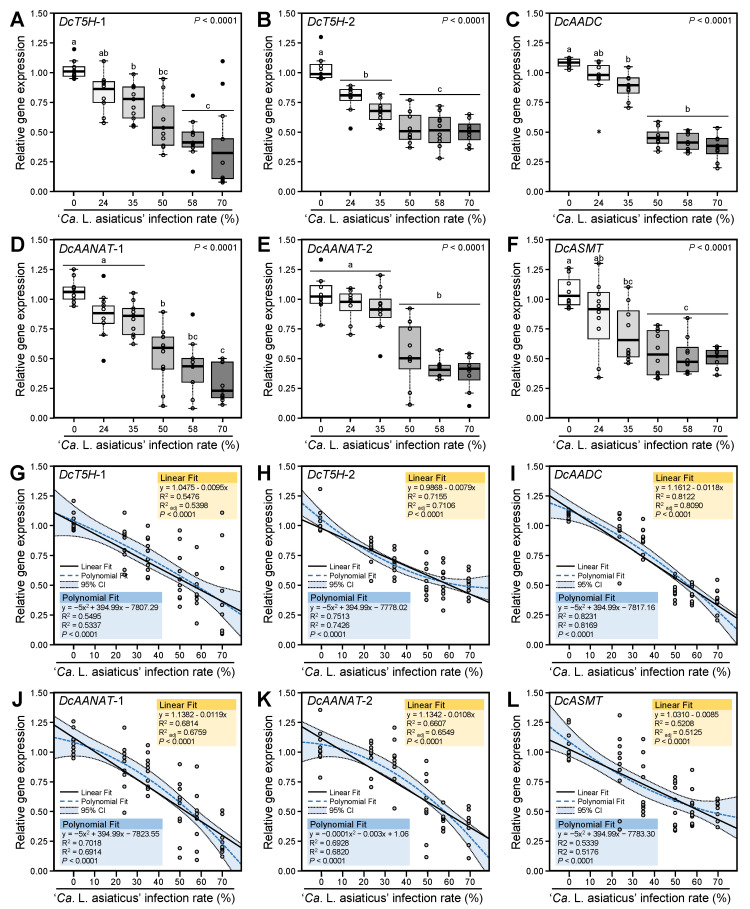
**Effect of different infection rates with *Candidatus* Liberibacter asiaticus on melatonin biosynthesis-related genes of *Diaphorina citri.*** (**A**–**F**) Relative gene expression of tryptophan 5-hydroxylase (*DcT5H*-1 and *DcT5H*-2), aromatic amino acid decarboxylase (*DcAADC*), arylalkylamine *N*-acetyltransferase genes (*DcAANAT*-1 and *DcAANAT*-2), and *N*-acetylserotonin O-methyltransferase (*DcASMT*), respectively. Gene expressions were normalized using two housekeeping genes (*α*-tubulin and actin), and the changes were analyzed using the 2^−ΔΔ^*^C^*_T_ method. Open dots indicate the raw data (*n* = 10), the black ones indicate potential outliers, whereas horizontal thick lines indicate the medians. Whiskers indicate the minimum, and the maximum values of the data and boxes show the interquartile ranges (twenty-fifth to the seventy-fifth percentile of the data). Different letters indicate statistically significant differences among different infection rates, while the same letter signifies no significant differences between them using Tukey–Kramer honestly significant difference test (Tukey HSD; *p* < 0.05). The full list of expressed genes, names, accession numbers, and primers are available in Table 1. (**G**–**L**) Simple linear regression and quadratic polynomial regression analysis between *Ca.* L. asiaticus infection rates and the expression levels of *DcT5H*-1, *DcT5H*-2, *DcAADC*, *DcAANAT*-1, *DcAANAT*-2, and *DcASMT*, respectively. Open dots present the row data (*n* = 10). The fitted regression line is presented as a black solid-line, while the polynomial regression model is presented as a blue dashed line. The 95% confidence intervals (95% CI) for the estimated polynomial regression are blue-shaded and edged by doted-lines. Regression equations, R^2^, R^2^_adj_, and *p*-value based on the F test (*p* < 0.05) were also obtained and presented within the graph.

We investigated the transcript levels of six melatonin biosynthesis-related genes in *D. citri* (Figure 14). These genes included two *DcT5H*s (*DcT5H*-1 and *DcT5H*-2), *DcAADC*, two *DcAANAT*s (*DcAANAT*-1 and *DcAANAT*-2), and *DcASMT* (Figure 14A–F, respectively). 

Our findings showed that the *Ca.* L. asiaticus infection significantly reduced the transcript levels of all studied melatonin biosynthesis-related genes. The downregulation of melatonin biosynthesis-related genes was proportionally over the *Ca.* L. asiaticus infection rates. Although the transcript levels of *DcT5H*-1 were gradually decreased with *Ca.* L. asiaticus infection rates, no significant differences were observed between the high *Ca.* L. asiaticus infection rates (58 and 70% *Ca.* L. asiaticus) (Figure 14A). Likewise, *Ca.* L. asiaticus infection decreased the expression levels of *DcT5H*-2; however, no significant differences were observed between low infection rates (24 and 35%), and also no significant differences were observed between the high infection rates (50, 58 and 70 % *Ca.* L. asiaticus) (Figure 14B). Moreover, *DcAADC* was downregulated in *Ca.* L. asiaticus-infected *D. citri* compared with healthy insects, without significant differences among the high infection rates (50, 58, and 70%) (Figure 14C). On the other hand, low infection rates (less than 50%) did not affect the expression levels of *DcAANAT*-1 and *DcAANAT*-2 (Figure 14D,E, respectively). The transcript levels *DcASMT* were also gradually reduced under different *Ca.* L. asiaticus infection rates without significant differences between the high infection rates (50, 58, and 70%) (Figure 14F).

### 3.3. Expression Levels of Melatonin Biosynthesis-Related Genes of D. citri Are Negatively Correlated with the Ca. L. asiaticus Infection Rates

The relationship between the expression levels of melatonin biosynthesis-related genes in *D. citri* and *Ca.* L. asiaticus infection rates is presented in Figure 14G–L. The simple linear regression showed that the expression levels of all melatonin biosynthesis-related genes were negatively correlated with the *Ca.* L. asiaticus infection rates (%) with moderate to high coefficient of determination (R^2^). The linear regression equations were expressed as follow: *DcT5H*-1 (y = 1.0475–0.0095x, R^2^ = 0.5476, R^2^_adj_ = 0.5398, and *p* < 0.0001; Figure 14G), *DcT5H*-2 (y = 0.9868–0.0079x, R^2^ = 0.7155, R^2^_adj_ = 0.7106, and *p* < 0.0001; Figure 14H), *DcAADC* (y = 1.1612–0.0118x, R^2^ = 0.8122, R^2^_adj_ = 0.8090, and *p* < 0.0001; Figure 14I), DcAANAT-1 (y = 1.1382–0.0119x, R^2^ = 0.6814, R^2^_adj_ = 0.6759, and *p* < 0.0001; Figure 14J), DcAANAT-2 (y = 1.1342–0.0108x, R^2^ = 0.6607, R^2^_adj_ = 0.6549, and *p* < 0.0001; Figure 14K), and *DcASMT* (y = 1.0310–0.0085x, R^2^ = 0.5208, R^2^_adj_ = 0.5125, and *p* < 0.0001; Figure 14L).

Due to the nonlinear phenomena of the obtained data, the expression levels of melatonin biosynthesis-related genes in *D. citri* and *Ca.* L. asiaticus infection rates were fitted with a second-degree polynomial regression model. The polynomial regression models and its associated 95% confident curves for *DcT5H*-1 (Figure 14G), *DcT5H*-2 (Figure 14H), *DcAADC* (Figure 14I), DcAANAT-1 (Figure 14J), DcAANAT-2 (Figure 14K), and *DcASMT* (Figure 14L) suggested that the relationship between the expression levels of melatonin biosynthesis-related genes in *D. citri* and *Ca.* L. asiaticus infection rates followed a negative and quadratic model with moderate to high R^2^ and R^2^_adj_.

### 3.4. Melatonin Supplementation Induced the Expression Levels of Melatonin Biosynthesis-Related Genes of D. citri

**Figure 15:**
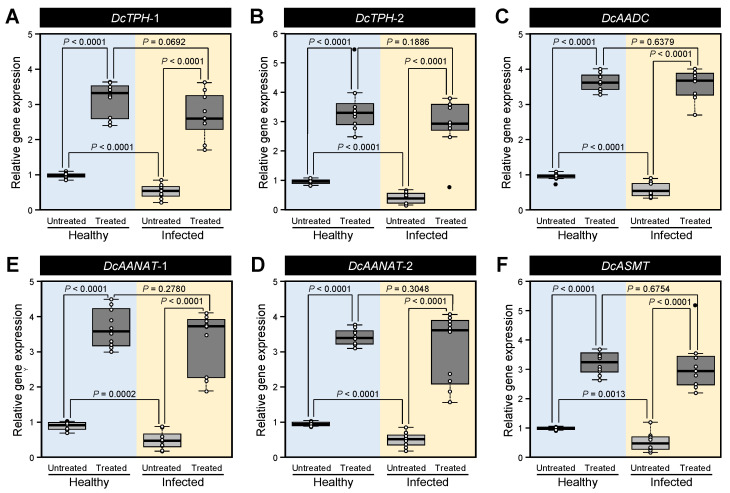
**Effect of supplementation on the expression levels of melatonin biosynthesis-related genes of healthy and *Ca.* L. asiaticus-infected psyllids, *Diaphorina citri*.** (**A**,**B**) Relative gene expression of tryptophan 5-hydroxylase (*DcT5H*-1 and *DcT5H*-2, respectively), (**C**) relative gene expression of aromatic amino acid decarboxylase (*DcAADC*), (**D**,**E**) relative gene expression of arylalkylamine N-acetyltransferase genes (*DcAANAT*-1 and *DcAANAT*-2, respectively), and (**F**) relative gene expression of *N*-acetylserotonin O-methyltransferase (*DcASMT*). Gene expressions were normalized using two housekeeping genes (*α*-tubulin and actin), and the changes were analyzed using the 2^−ΔΔ^*^C^*_T_ method. Open dots indicate the raw data (*n* = 10), the black ones indicate potential outliers, whereas horizontal thick lines indicate the medians. Whiskers indicate the minimum, and the maximum values of the data and boxes show the interquartile ranges (twenty-fifth to the seventy-fifth percentile of the data). Treatments (healthy versus infected and untreated versus treated) were compared using a two-tailed *t*-test, and statistical significance was established as *p* < 0.05. The full list of expressed genes, names, accession numbers, and primers are available in Table 1.

We investigated the transcript levels of the melatonin biosynthesis-related genes in healthy and *Ca.* L. asiaticus-infected *D. citri* adults after treatment with exogenous melatonin (Figure 15). Generally, the pretreatment with 500 μg mL^−1^ melatonin upregulated the gene expression of all studied genes in both healthy and infected insects. Although the expression levels of melatonin biosynthesis-related genes were lower in *Ca.* L. asiaticus-infected psyllids compared with the control (healthy adults), exogenous melatonin supplementation enhanced the transcript levels of all studied genes to reach the control without significant differences between them (treated-control versus treated-infected psyllids). Accordingly, these findings suggest that melatonin supplementation enhanced the gene expression of melatonin biosynthetic genes of *D. citri*.

## 4. Discussion

Previously, we reported that *Ca.* L. asiaticus infection significantly reduced the endogenous melatonin content of *D. citri* [15]. However, it is not clear whether this may happen due to the utilization of insect melatonin directly by *Ca.* L. asiaticus, or if it was a common cause due to the *Ca.* L. asiaticus infection, which might affect the physiological and transcriptional capacities of *D. citri*. The genome sequencing of *Ca.* L. asiaticus revealed that it cannot synthesize the amino acid _L_-tryptophan, the precursor of melatonin [72], from metabolic intermediates [73], and it should acquire it from its host. Further, there is no evidence for melatonin biosynthesis by *Ca.* L. asiaticus which supports the idea that *Ca.* L. asiaticus depends on its host (psyllid vectors or citrus plant) for its melatonin needs. Nevertheless, recently we showed that melatonin might play an antibacterial role against *Ca.* L. asiaticus [18], which suggests that utilization of melatonin by *Ca.* L. asiaticus is not the main reason for melatonin reduction within infected psyllid. 

Furthermore, another potential reason for the reduction of melatonin is that it could be a common cause due to the *Ca.* L. asiaticus infection, which disrupts the physiological and transcriptional capacities of *D. citri* [13,14,15], particularly melatonin biosynthesis-related genes. However, to the best of our knowledge, melatonin biosynthetic genes are not well-annotated yet from the Asian citrus psyllid, *D. citri.* Previously, we roughly identified four genes to be associated with the melatonin biosynthesis in *D. citri*, which were downregulated in *Ca.* L. asiaticus-infected psyllids [15]. However, some of these genes have been updated based on the most recent available data in GenBank, the national center for biotechnology information website (NCBI, http://www.ncbi.nlm.nih.gov/gene/, 12 February 2021) including PREDICTED: *Diaphorina citri* protein henna-like (LOC103524631; GenBank Accession no. XM_017449691.2), PREDICTED: *Diaphorina citri* tyrosine 3-monooxygenase (LOC103505706; GenBank Accession no. XM_017442547.2), and PREDICTED: *Diaphorina citri* aromatic L-amino acid decarboxylase-like (LOC103510318; GenBank Accession no. XM_017444526.2), and, as a result of standard genome annotation processing (see www.ncbi.nlm.nih.gov/genome/annotation_euk/process/, 12 February 2021 for more information), even some sequences were removed including PREDICTED: *Diaphorina citri* dopamine N-acetyltransferase-like (LOC103507708; GenBank Accession no. XM_008472208.2) and PREDICTED: *Diaphorina citri* arf-GAP domain and FG repeat-containing protein 1 (LOC103510708; GenBank Accession no. XM_017444656.1). 

**Figure 16:**
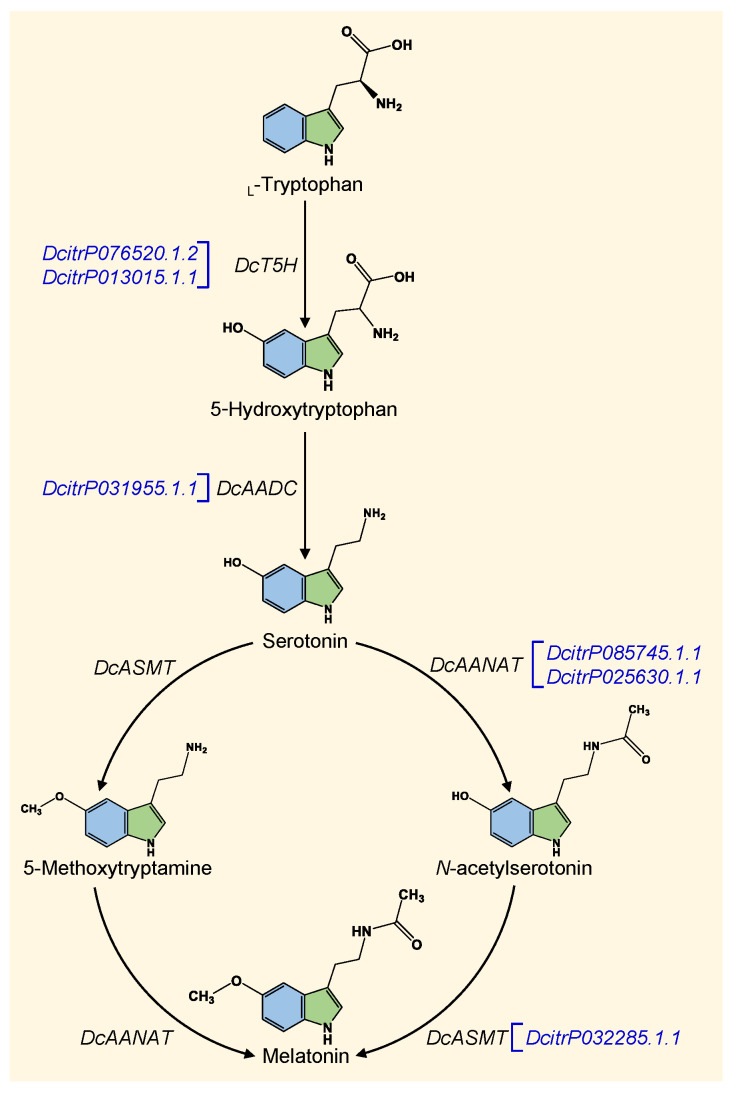
**Proposed melatonin biosynthesis pathway in *Diaphorina citri* and its associated genes.** The listed putative gene candidates were identified based on the top-matches of recent available data on the “*Diaphorina citri* OGS v2.0 proteins” dataset available on the citrus greening solutions website (https://citrusgreening.org/organism/Diaphorina_citri/genome, 12 February 2021). *DcT5H*: Tryptophan 5-hydroxylase, *DcAADC*: Aromatic amino acid decarboxylase, *DcAANAT*: Arylalkylamine N-acetyltransferase, and *DcASMT*: *N*-acetylserotonin O-methyltransferase.

Therefore, herein, we carried out a comprehensive in silico and bioinformatics analysis to deeply identify the melatonin biosynthesis-related genes of *D. citri* using two major databases including the *D. citri*-specific dataset of “*Diaphorina citri* OGS v2.0 proteins” available on Citrus Greening Solutions website (https://citrusgreening.org/organism/Diaphorina_citri/genome, 12 February 2021) [58] and the most popular database of GenBank, the national center for biotechnology information website (NCBI, http://www.ncbi.nlm.nih.gov/gene/, 12 February 2021). A proposed melatonin biosynthesis pathway and its associated genes in *D. citri* is presented in Figure 16. 

Briefly, we suggest that melatonin in *D. citri* is synthesized from the _L_-tryptophan via four enzymatic steps. The first step is the oxidation of _L_-tryptophan to 5-hydroxytryptophan [74] via the activity of *DcT5H*. Our findings showed that the *D. citri* genome possesses at least two sequences that were relatively homologous and were phylogenetically closer to *DmT5H* from *D. melanogaster* [48,49] including putative tryptophan 5-hydroxylase 1-like (*DcT5H*-1; DcitrP076520.1.1) and protein henna (*DcT5H*-2; DcitrP012845.1.1). *T5H* is the rate-limiting enzyme in serotonin biosynthesis [28,29] that works in the presence of molecular oxygen and requires tetrahydrobiopterin as a cofactor. 

Our InterPro-based analysis showed that both *DcT5Hs* have a tetrahydrobiopterin-dependent aromatic amino acid (ArAA hydroxylase) family [75] that catalyzes ring hydroxylation of aromatic amino acids, using tetrahydrobiopterin (BH4) as a substrate. Previous studies showed that all eukaryotic *T5H* are homotetramers and include a regulatory N-terminal domain, a catalytic domain, and a C-terminal oligomerization motif [76,77]. In agreement with these studies, the crystallographic 3D structures of both *DcT5Hs* modeled with the crystal structure of human tryptophan hydroxylase 2 (*TPH2*), catalytic domain (4v06.1.A) [78], encompassed two close conserved histidines that are involved in the binding to iron [79] and one more distant acidic residue, usually glutamic acid in our predicted models. This arrangement of ligands suggests that *DcT5Hs* are metalloproteins. Interestingly, the expression levels of both *DcT5Hs* were decreased in *Ca.* L. asiaticus-infected psyllids and showed an almost identical profile with endogenous melatonin content from our previous study [15]. Collectively, these findings suggest that *DcT5Hs* (*DcT5H*-1 and *DcT5H*-2) could play a key role in melatonin biosynthesis. However, further studies are required to clarify the functional and/or regulatory roles of *DcT5Hs* in *D. citri*.

The second step in the melatonin biosynthesis pathway is the decarboxylation of 5-hydroxytryptophan to the indoleamine serotonin using the pyridoxal 5-phosphate (PLP)-dependent enzyme aromatic L-amino acid decarboxylase (*AADC*) [28,40]. Our findings showed that the *D. citri* genome could encode for a putative Dopa decarboxylase (*DcDDC*; DcitrP031955.1.1) that produced significant similarities to *DmDDC* (also known as *DmAADC*) from *D. melanogaster* [50,51,52]. This protein was described in the current study as *DcAADC*, indicating its aromatic L-amino acid decarboxylase activity. Based on the InterPro analysis, this enzyme is a pyridoxal phosphate-dependent decarboxylase that belongs to the group II decarboxylases [80,81] and shares a region of a conserved lysine residue, which provides the binding site for the PLP group [80,82]. Further, InterPro analysis showed that *DcAADC* seems to share regions of sequence similarity with aromatic-L-amino acid decarboxylase (*DDC*; also known as L-dopa decarboxylase or tryptophan decarboxylase), glutamate decarboxylase (*GAD*), histidine decarboxylase (*HDC*), and tyrosine decarboxylase (*TyrDC*) [80,81,82,83] since it had a DDC/GAD/HDC/TyrDC pyridoxal-phosphate attachment site. These enzymes are collectively known as group II decarboxylases [81]. 

Moreover, our findings suggest that *DcAADC* might act as an amino acid-transporter and could be essential for primary carbon metabolism. Although the homo-oligomerization state of AADC proteins has not been extensively investigated, our findings suggest that *DcAADC* might form a homodimer in the membrane; nevertheless, previous studies suggest that this is dispensable for transport [84]. Our findings showed that *Ca.* L. asiaticus infection diminished the transcript levels of *DcAAAD* of *D. citri* compared to uninfected psyllids. The reduction in the *DcAAAD* expression was consistent with the profile of endogenous melatonin upon different *Ca.* L. asiaticus infection rates as reported in our previous study [15]. Moreover, melatonin supplementation enhanced the *DcAADC* expression and reverses the negative effects of *Ca.* L. asiaticus. Taken together, these findings suggest that *DcAADC* might play a dual role in melatonin biosynthesis and amino acid transportation. However, further studies are required to investigate the functional and/or regulatory roles of *DcAADC* in *D. citri*.

The third step in the melatonin biosynthesis pathway is the N-acetylation of serotonin to form N-acetylserotonin using serotonin *N*-acetyltransferase (SNAT; also known as arylalkylamine *N*-acetyltransferase [AANAT]) [29]. Our findings showed that the *D. citri* genome possesses two putative dopamine *N*-acetyltransferase proteins (DcitrP025630.1.1 and DcitrP085745.1.1) with significant similarity to arylalkylamine *N*-acetyltransferase 1, isoform A (*DmAANAT1*) from *D. melanogaster* [53,54,55]. Both proteins were described in the current study as arylalkylamine *N*-acetyltransferase (*DcAANAT*-1 and *DcAANAT*-2) indicating their N-acetylation activity. These findings are in agreement with studies on *D. melanogaster* where two AANAT variants (AANATA and AANATB) were identified [59,85]. Although both variants were physiologically relevant in *D. melanogaster*, they were differentially expressed with respect to tissue distribution and developmental stages [59,85]. In contrast, the expression patterns of *DcAANAT*-1 and *DcAANAT*-2 in the current study were almost identical with slight differences which suggest that both enzymes serve the same metabolic role in *D. citri*. 

AANAT is an acetyl-CoA-dependent enzyme that belongs to *GCN5 N-acetyltransferases (GNATs)* family [86]. It is the penultimate enzyme in melatonin biosynthesis that catalyzes the transfer of the acetyl group of acetyl-CoA to the primary amine of serotonin to form N-acetylserotonin and CoA [86]. In the current study, InterPro analysis showed that both DcAANAT-1 and DcAANAT-2 have a structural domain of Acyl-CoA N-acyltransferase homologous superfamily. This domain has a triple-layer α/β/α structure that contains mixed β-sheets. The crystallographic 3D structures showed that DcAANAT-1 and DcAANAT-2 have three antiparallel β-strands like DmAANAT. Although coenzyme A binding pockets were observed via the InterPro analysis, no acyl-CoA binding sites were observed in the tertiary structures of DcAANAT-1, and DcAANAT-2. Our findings in the current study showed that low infection rates of *Ca.* L. asiaticus (less than 50%) did not affect the expression levels of DcAANAT-1 and DcAANAT-2; however, our previous study showed that low infection rates significantly reduced the endogenous melatonin content [15]. On the other hand, the higher infection rates reduced the expression levels of both genes in agreement with the endogenous melatonin profile of our previous study [15]. Together, these findings suggest that DcAANAT may not be a rate-determining enzyme in melatonin biosynthesis in *D. citri*. However, further studies are required to investigate the functional and/or regulatory roles of DcAANAT in *D. citri*.

Finally, N-acetylserotonin is subsequently methylated by *N*-acetylserotonin O-methyltransferase (ASMT) as a final step in the melatonin production [28,30,31,32]. Our findings showed that the *D. citri* genome encodes for a putative *N*-acetylserotonin O-methyltransferase-like (*DcASMT*; DcitrP032285.1.1) that is producing significant similarities to *N*-acetylserotonin O-methyltransferase-like protein, isoform X1 (*ClASMT*) from bed bug (*C. lectularius*). Herein, we used *ClASMT* from bed bug as a query sequence because, to the best of our knowledge, no ASMT genes have been identified from *D. melanogaster* yet. Like other melatonin biosynthesis-related genes in this study, *DcASMT* was significantly downregulated in *Ca.* L. asiaticus-infected psyllids and agreed with the melatonin profile in our previous study [15].

Moreover, we proposed an alternative route for melatonin biosynthesis from serotonin (Figure 16). Briefly, we suggest that serotonin could be methylated first, rather than N-acetylated as discussed above, to form 5-methoxytryptamine using ASMT. Subsequently, 5-methoxytryptamine is *N*-acetylated to form melatonin using SNAT. However, a previous study showed that the enzymatic activity of ASMT was about 14-times greater when it reacted with N-acetylserotonin than when it reacted with serotonin [87] which suggests methylation, but not *N*-acetylation, of N-acetylserotonin, as the last step of melatonin. Nevertheless, this alternative route might occur under specific circumstances [31,33]. As summarized above, melatonin biosynthesis is controlled by four successive enzymes; however, SNAT was proposed to be the key rate-limiting enzyme in this pathway in vertebrates [28], but not at night [88]. Instead, ASMT may be a rate-limiting enzyme during the nocturnal production of melatonin [88].

## 5. Conclusions

In conclusion, in the present study, we computationally identified six melatonin biosynthesis-related gene candidates (two *DcT5Hs*, one *DcAADC*, two *DcAANATs*, and one *DcASMT*) in *D. citri.* These genes were definitionally expressed within the adults of *D. citri* after challenging with gradient infection rates of the phytopathogenic bacterium *Ca.* L. asiaticus. Moreover, the expression patterns of these genes demonstrated a piece of indirect evidence for the return of melatonin to its normal levels in *Ca.* L. asiaticus-infected psyllids after melatonin supplementation and confirmed the association of these genes with the melatonin biosynthesis pathway. However, further investigations are required to explore the functional and/or regulatory roles of these genes in melatonin biosynthesis. Our findings of this study are a further step for optimization and cloning of melatonin biosynthesis genes of *D. citri*. They could rapidly be identified via in silico analysis and subsequently subjected to in vitro and in vivo confirmatory studies, since our previous study showed that inhibition of melatonin biosynthesis was associated with reduced longevity of *D. citri* [15]. Therefore, the identified genes in this study could be good candidates that serve as potential targets for RNA interference (RNAi)-based control or other sustainable control strategies of *D. citri.*

## Figures and Tables

**Table 1:** Alignment statistics for the top-matched sequences producing significant alignments of melatonin-biosynthetic genes.

NCBI Database ^a^													*D. citri* Genome Database ^b^							
Gene Description	Gene ID	mRNA		Protein				Protein–Protein Alignment Statistics					Gene Description	mRNA		Protein		Protein–Protein Alignment Statistics		
		Accession	bp	Accession	aa	Theoretical Isoelectric Point (pI)	Molecular Weight (MW)	Max Score	Total Score	Query Cover (%)	E Value	Identity (%)		Accession	bp	Accession	aa	Identities (%)	Positives (%)	E Value
***DcT5H*** ^c^													***DcT5H***							
Tryptophan 5-hydroxylase 1-like	LOC113470334	XM_026828703.1	1343	XP_026684504.1	379	5.64	43,291.92	464	464	51	1 × 10^−161^	75.69	Tryptophan 5-hydroxylase, putative	DcitrC076520.1.1	591	DcitrP076520.1.1	196	100	100	7 × 10^−143^
Protein henna-like	LOC103524631	XM_017449691.2	1298	XP_017305180.1	319	5.67	36,344.09	363	363	58	5 × 10^−123^	56.17	Protein henna	DcitrC012845.1.1	1884	DcitrP012845.1.1	627	87.15	87	0.0
***DcAADC*** ^d^													***DcAADC* (also known as *DcDDC*)**							
Aromatic-L-amino-acid decarboxylase isoform X1	LOC103520978	XM_008486080.3	1939	XP_008484302.1	481	5.70	54,429.72	734	734	90	0.0	75.27	Dopa decarboxylase	DcitrC031955.1.1	1446	DcitrP031955.1.1	481	100	100	0.0
Aromatic-L-amino-acid decarboxylase	LOC103510318	XM_017444526.2	1737	XP_017300015.1	484	6.61	54825.40	692	692	90	0.0	71.61	Dopa decarboxylase	DcitrC031955.1.1	1446	DcitrP031955.1.1	481	93.83	94	0.0
***DcAANAT* (also known as *DcSNAT*)** ^e^													***DcAANAT* (also known as *DcSNAT*)**							
Dopamine N-acetyltransferase-like isoform X1	LOC103507708	XM_026822511.1	1221	XP_026678312.1	217	6.26	24,749.18	67.4	67.4	85	1 × 10^−13^	30.19	Dopamine N-acetyltransferase	DcitrC025630.1.1	654	DcitrP025630.1.1	217	99.08	99	8 × 10^−161^
Dopamine N-acetyltransferase-like	LOC103507696	XM_017443457.2	1911	XP_017298946.1	220	5.39	24,808.39	114	114	83	2 × 10^−31^	34.78	Dopamine N-acetyltransferase	DcitrC085745.1.1	723	DcitrP085745.1.1	240	33.01	51.46	4 × 10^−30^
***DcASMT*** ^f^													***DcASMT***							
Septum formation protein Maf-like	LOC113468045	XM_026824671.1	746	XP_026680472.1	162	5.26	18,064.58	153	153	68	3 × 10^−47^	50.00	N-acetylserotonin O-methyltransferase-like	DcitrC032285.1.1	825	DcitrP032285.1.1	274	98.77	100	5 × 10^−115^

^a^ The listed putative gene candidates were identified based on the top-matches of recent available data in GenBank, the national center for biotechnology information website (NCBI, http://www.ncbi.nlm.nih.gov/gene/, 12 February 2021) with the query sequences. The shortlist of top-matches was generated based on the phylogenetic trees, identity more than 50% (except for *DcAANAT*s), and excluding all the hypothetical and low-quality proteins that have these characteristics. ^b^ The listed genes are the top-matched sequences of the “*Diaphorina citri* OGS v2.0 CDS” and “*Diaphorina citri* OGS v2.0 proteins” BLAST datasets available on the Citrus Greening Solutions website (https://citrusgreening.org/organism/Diaphorina_citri/genome, 12 February 2021) using the Nucleotide–Nucleotide BLAST (BLASTn) and Protein–Protein BLAST (BLASTp) tools [56,57] using the NCBI query sequences. ^c^ Top-matched sequences from *D. citri* with tryptophan hydroxylase (GenBank Accession no. NP_612080.1) from fruit fly (*Drosophila melanogaster*) [49] using the Protein–Protein BLAST (BLASTp), based on recent available data in GenBank, the national center for biotechnology information website (NCBI, http://www.ncbi.nlm.nih.gov/gene/, 12 February 2021), using the compositionally adjusted substitution matrices [57]. ^d^ Top-matched sequences from *D. citri* with dopa decarboxylase, isoform B (GenBank Accession no. NP_724164.1) from fruit fly (*D. melanogaster*) [51,52] using the Protein–Protein BLAST (BLASTp), based on recently available data in GenBank, the national center for biotechnology information website (NCBI, http://www.ncbi.nlm.nih.gov/gene/, 12 February 2021), using the compositionally adjusted substitution matrices [57]. ^e^ Top-matched sequences from *D. citri* with arylalkylamine N-acetyltransferase 1, isoform A (GenBank Accession no. NP_523839.2) from the fruit fly (*D. melanogaster*) [53,54,59] using the Protein–Protein BLAST (BLASTp), based on recently available data in GenBank, the national center for biotechnology information website (NCBI, http://www.ncbi.nlm.nih.gov/gene/, 12 February 2021), using the compositionally adjusted substitution matrices [57]. ^f^ Top-matched sequences from *D. citri* with N-acetylserotonin O-methyltransferase-like protein, isoform X1 (GenBank Accession no. XP_014251646.1) from bed bug (*Cimex lectularius*) using the Protein–Protein BLAST (BLASTp), based on recently available data in GenBank, the national center for biotechnology information website (NCBI, http://www.ncbi.nlm.nih.gov/gene/, 12 February 2021), using the compositionally adjusted substitution matrices [57].

## Data Availability

Data will be shared upon request to the corresponding author.

## Supplementary Materials

The following supplementary materials are available online at https://www.mdpi.com/article/10.3390/insects12040317/s1, Table S1. Primers used for gene expression analysis of melatonin-biosynthetic enzymes of *D. citri* by real-time RT-PCR. Table S2. Sequences from *Diaphorina citri* that produce significant alignments with tryptophan hydroxylase from *Drosophila melanogaster* (*T5H*; GenBank Accession no. NP_612080.1) using the NCBI database. Table S3. Sequences from *Diaphorina citri* that produce significant alignments with tryptophan hydroxylase from *Drosophila melanogaster* (*T5H*; GenBank Accession no. NP_612080.1) using the “*Diaphorina citri* OGS v2.0 proteins” BLAST dataset. Table S4. Sequences from *Diaphorina citri* that produce significant alignments with dopa decarboxylase, isoform B (GenBank Accession no. NP_724164.1) from *Drosophila melanogaster* using the NCBI database. Table S5. Sequences from *Diaphorina citri* that produce significant alignments with dopa decarboxylase, isoform B (GenBank Accession no. NP_724164.1) from *Drosophila melanogaster* using the “*Diaphorina citri* OGS v2.0 proteins” BLAST dataset. Table S6. Sequences from *Diaphorina citri* that produce significant alignments with arylalkylamine N-acetyltransferase 1, isoform A (*AANAT1*; GenBank Accession no. NP_523839.2) from *Drosophila melanogaster* using the NCBI database. Table S7. Sequences from *Diaphorina citri* that produce significant alignments with arylalkylamine N-acetyltransferase 1, isoform A (*AANAT1*; GenBank Accession no. NP_523839.2) from *Drosophila melanogaster* using the “*Diaphorina citri* OGS v2.0 proteins” BLAST dataset. Table S8. Sequences from *Diaphorina citri* that produce significant alignments with N-acetylserotonin O-methyltransferase-like protein (*ASMT*; GenBank Accession no. XP_014251646.1) from bed bug (*Cimex lectularius*) using the NCBI database. Table S9. Sequences from *Diaphorina citri* that produce significant alignments with N-acetylserotonin O-methyltransferase-like protein (*ASMT*; GenBank Accession no. XP_014251646.1) from bed bug (*Cimex lectularius*) using the “*Diaphorina citri* OGS v2.0 proteins” BLAST dataset. Figure S1. Multiple AA sequence alignment of *DcT5H*-1 from *Diaphorina citri*. Figure S2. Multiple AA sequence alignment of *DcT5H*-2 from *Diaphorina citri*. Figure S3. Multiple nucleotide sequence alignment of *DcT5H*-1 from *Diaphorina citri*. Figure S4. Multiple nucleotide sequence alignment of *DcT5H*-2 from *Diaphorina citri*. Figure S5. Multiple AA sequence alignment of *DcAADC* from *Diaphorina citri*. Figure S6. Multiple nucleotide sequence alignment of *DcT5H*-1 from *Diaphorina citri*. Figure S7. Multiple AA sequence alignment of *DcAANAT*-1 from *Diaphorina citri*. Figure S8. Multiple AA sequence alignment of *DcAANAT*-2 from *Diaphorina citri*. Figure S9. Multiple nucleotide sequence alignment of *DcAANAT*-1 from *Diaphorina citri*. Figure S10. Multiple nucleotide sequence alignment of *DcAANAT*-2 from *Diaphorina citri*. Figure S11. Multiple AA sequence alignment of *DcASMT* from *Diaphorina citri*. Figure S12. Multiple nucleotide sequence alignment of *DcASMT* from *Diaphorina citri*.
