# Supplementary material for: Diaphorina citri Genome Possesses a Complete Melatonin Biosynthesis Pathway Differentially Expressed under the Influence of the Phytopathogenic Bacterium, Candidatus Liberibacter asiaticus
Source: Insects. 2021 Apr 1;12(4):317. doi: 10.3390/insects12040317 (PMC8065666; doi:10.3390/insects12040317)
Supplement: Supplementary file 1 [file insects-12-00317-s001.pdf]

SUPPLEMENTARY MATERIALS:

Diaphorina citri genome Possesses a Complete Melatonin Biosynthesis Pathway Differentially Expressed Under the Influence of the Phytopathogenic Bacterium, '*Candidatus* Liberibacter asiaticus'

Yasser Nehela[1,2] and Nabil Killiny[1*]

[1] Department of Plant Pathology, Citrus Research and Education Center, University of Florida, 700 Experiment Station Rd., Lake Alfred, FL 33850

[2] Department of Agricultural Botany, Faculty of Agriculture, Tanta University, Tanta, Egypt

Corresponding author: Nabil Killiny

Department of Plant Pathology,
Citrus Research and Education Center
University of Florida
700 Experiment Station Rd.
Lake Alfred, FL, 33850
Phone: 863-956-8833
Email: nabilkilliny@ufl.edu

Running title: Melatonin biosynthesis pathway in *D. citri*.

Supplementary Tables:

Table S1. Primers used for gene expression analysis of melatonin-biosynthetic enzymes of *D. citri* by real time RT-PCR [a].

Gene	NCBI Reference ID	OGS-v2 Protein name (Protein ID)		Primer (Forward and Reverse)	TM (°C)	Product size (bp)
***DcT5H* -1** [b]	XM_026828703.1	DcitrP076520.1.1	F	TTAACCCATGCTGTCACGAA	60.1	220
			R	TGCTCAGCACTTCCACACTC	60.2	
***DcT5H* -2** [b]	XM_017449691.2	DcitrP012845.1.1	F	CGCTACCCCGTGTTGACTAT	60.0	241
			R	TCACGTGATGAGAGCAGACC	60.0	
DcAADC [c]	XM_008486080.3	DcitrP031955.1.1	F	CCGTAGGTTCAGATCCCTCA	60.1	177
	XM_017444526.2		R	AGTCGGAAACACACCAGTCC	60.0	
***DcAANAT*-1** [d]	XM_017443457.2	-	F	TTCAGCACGAAGGAGATGTG	60.0	205
			R	AACAGTTCATTGGCCAGTCC	60.0	
***DcAANAT*-2** [d]	XM_026822511.1	DcitrP025630.1.1	F	CATGACATCAAGGCCATCAC	59.9	166
			R	ACTCCACATGAGGCTCTGCT	60.0	
DcASMT [e]	XM_026824671.1	DcitrP032285.1.1	F	AAAATTGTCCTGGCAAGCAG	60.2	212
	XM_026832789.1		R	AAATCCGGCTCAACATTGTC	59.9	
α-Tubulin [f]	-	-	F	CAGGTCTTGTGTGGGACGTA	-	-
			R	GGCCACAGTTTGTTTCTTGC	-	
Actin [f]	-	-	F	CCCTGGACTTTGAACAGGAA	-	-
			R	CTCGTGGATACCGCAAGATT	-	

[a] The listed genes were identified using the nucleotide-nucleotide BLAST (BLASTn) and using the protein-protein BLAST (BLASTp), based on recent available data in national center for biotechnology information website (NCBI, http://www.ncbi.nlm.nih.gov/gene/) and the available Official Gene Set (OGS) for *D. citri* "*Diaphorina citri* OGS v2.0 CDS" and "*Diaphorina citri* OGS v2.0 proteins" available on Citrus Greening Solutions website (https://citrusgreening.org/organism/Diaphorina_citri/genome).

[b] *Diaphorina citri* tryptophan 5-hydroxylase 1-like (*DcTPH* -1; DcitrP076520.1.1) and PREDICTED: *Diaphorina citri* protein henna-like (*DcTPH* -2; DcitrP012845.1.1) have been matched using tryptophan hydroxylase (*DmTPH-1*; GenBank Accession no. NP_612080.1) gene from fruit fly (*Drosophila melanogaster*).

[c] PREDICTED: *Diaphorina citri* aromatic-L-amino-acid decarboxylase (*DcAADC*; also known as Dopa decarboxylase; DcitrP031955.1.1) has been matched using dopa decarboxylase - isoform B (*DmDDC*; GenBank Accession no. NP_724164.1) from fruit fly (*Drosophila melanogaster*).

[d] PREDICTED: *Diaphorina citri* dopamine N-acetyltransferase-like (also known as serotonin N-acetyltransferase; *DcAANAT*-1 and *DcAANAT*-2 [DcitrP025630.1.1]), have been matched using arylalkylamine N-acetyltransferase 1, isoform A (*DmAANAT1*; GenBank Accession no. NP_523839.2) from fruit fly (*Drosophila melanogaster*).

[e] *N*-acetylserotonin O-methyltransferase-like (*DcASMT*; DcitrP032285.1.1) from *Diaphorina citri* has been matched using *N*-acetylserotonin O-methyltransferase-like protein - isoform X1 (GenBank Accession no. XP_014251646.1) from bed bug (*Cimex lectularius*).

[f] Genes have been used as a reference genes for data normalization according to Tiwari *et al*. [1] and Killiny *et al*. [2].

Abbreviations

- ***DcTPH***: *Diaphorina citri*-Tryptophan 5-hydroxylase
- ***DcAAAD:*** PREDICTED: *Diaphorina citri* aromatic-L-amino-acid decarboxylase-like (also known as Dopa decarboxylase)
- ***DcAANAT:*** *Diaphorina citri* arylalkylamine N-acetyltransferase (also known as Serotonin N-acetyltransferase [SNAT] or Dopamine N-acetyltransferase)
- ***DcASMT***: N-acetylserotonin O-methyltransferase

Table S2. Sequences from *Diaphorina citri* that producing significant alignments with tryptophan hydroxylase from *Drosophila melanogaster* (GenBank Accession no. NP_612080.1) using NCBI database [a]

Description	NCBI Accession number	Accession Length (aa)	Max Score	Total Score	Query Cover (%)	E value	Identity (%)
Tryptophan 5-hydroxylase 1-like	XP_026684504.1	379	464	464	51	1e-161	75.69
Protein henna-like	XP_008487884.2	289	158	222	32	3e-44	65.18
Protein henna-like	XP_017305180.1	319	363	363	58	5e-123	56.17
Protein henna-like - partial	XP_008482682.2	86	76.3	76.3	14	3e-17	48.75
Tyrosine 3-monooxygenase	XP_017298036.2	457	359	359	77	3e-119	43.91
Tyrosine 3-monooxygenase-like - partial	XP_008485089.3	305	92.0	92.0	28	1e-20	33.96

[a] The listed genes were identified using the protein-protein BLAST (BLASTp) using tryptophan hydroxylase (GenBank Accession no. NP_612080.1) from *Drosophila melanogaster* [3], as a query sequence, against *Diaphorina citri* genome available in GenBank, national center for biotechnology information website (NCBI, http://www.ncbi.nlm.nih.gov/gene/), using the compositionally adjusted substitution matrices [4].

Table S3. Sequences from *Diaphorina citri* that producing significant alignments with tryptophan hydroxylase from *Drosophila melanogaster* (GenBank Accession no. NP_612080.1) using "*Diaphorina citri* OGS v2.0 proteins" BLAST dataset [a]

**Description**	Dcitr_OGSv2.0 **Accession number**	**Length (aa)**	**Identities (%)**	**Positives (%)**	**Gaps (%)**	**E value**	**Score**
Protein henna	DcitrP012845.1.3	627	185/424 (43.63)	246/424 (58.02)	65/424 (15.33)	1e-109	340
Protein henna	DcitrP012845.1.2	627	185/424 (43.63)	246/424 (58.02)	65/424 (15.33)	1e-109	340
Protein henna	DcitrP012845.1.1	627	185/424 (43.63)	246/424 (58.02)	65/424 (15.33)	1e-109	340
Tyrosine hydroxylase	DcitrP088565.1.1	571	178/435 (40.92)	252/435 (57.93)	41/435 (9.43)	3e-102	319
Tryptophan 5-hydroxylase, putative	DcitrP076520.1.2	196	131/185 (70.81)	158/185 (85.41)	4/185 (2.16)	2e-86	266
Tryptophan 5-hydroxylase, putative	DcitrP076520.1.1	196	131/185 (70.81)	158/185 (85.41)	4/185 (2.16)	2e-86	266
Retrovirus-related Pol polyprotein from transposon TNT 1-94	DcitrP020940.1.1	460	78/94 (82.98)	87/94 (92.55)	0/94 (0.00)	3e-52	184
Tryptophan hydroxylase	DcitrP013015.1.1	220	49/66 (74.24)	54/66 (81.82)	0/66 (0.00)	6e-28	111
Tryptophan hydroxylase	DcitrP020945.1.1	112	34/51 (66.67)	44/51 (86.27)	0/51 (0.00)	9e-21	87.4

[a] The listed genes were identified using the protein-protein BLAST (BLASTp) using tryptophan hydroxylase (GenBank Accession no. NP_612080.1) from *Drosophila melanogaster* [3], as a query sequence, against "*Diaphorina citri* OGS v2.0 proteins" BLAST datasets available on Citrus Greening Solutions website (https://citrusgreening.org/organism/Diaphorina_citri/genome).

Table S4. Sequences from *Diaphorina citri* that producing significant alignments with dopa decarboxylase, isoform B (GenBank Accession no. NP_724164.1) from *Drosophila melanogaster* using NCBI database [a]

Description	Accession number	Accession Length (aa)	Max Score	Total Score	Query Cover (%)	E value	Identity (%)
Aromatic-L-amino-acid decarboxylase isoform X1	XP_008484302.1	481	734	734	96	0.0	75.27
Aromatic-L-amino-acid decarboxylase	XP_017300015.1	484	691	691	96	0.0	71.61
Aromatic-L-amino-acid decarboxylase-like	XP_026680193.1	93	91.7	91.7	14	1e-22	60.29
Aromatic-L-amino-acid decarboxylase-like	XP_008486039.2	96	127	127	19	1e-35	59.57
Alpha-methyldopa hypersensitive protein-like	XP_008476868.1	359	422	422	73	2e-146	55.84
Alpha-methyldopa hypersensitive protein-like	XP_017304386.1	269	149	149	27	7e-42	51.16
Alpha-methyldopa hypersensitive protein-like	XP_026682704.1	276	149	149	27	7e-42	51.16
Alpha-methyldopa hypersensitive protein-like	XP_026679289.1	463	328	328	67	5e-108	47.84
Histidine decarboxylase-like	XP_017299796.1	200	225	225	50	6e-72	46.86
Tyrosine decarboxylase-like	XP_026676999.1	812	410	410	85	8e-136	45.29
Histidine decarboxylase	XP_008477680.1	725	426	426	99	5e-143	44.40
Acidic amino acid decarboxylase GADL1-like	XP_008486117.1	96	60.1	60.1	18	1e-11	37.78
Glutamate decarboxylase isoform X1	XP_008481145.1	511	135	135	88	1e-34	27.25
Acidic amino acid decarboxylase GADL1	XP_017299466.1	352	101	101	62	1e-23	25.55
Cysteine sulfinic acid decarboxylase	XP_008473394.1	586	81.6	81.6	88	1e-16	22.70

[a] The listed genes were identified using the protein-protein BLAST (BLASTp) using dopa decarboxylase, isoform B (GenBank Accession no. NP_724164.1) from *Drosophila melanogaster* [5,6], as a query sequence, against *Diaphorina citri* genome available in GenBank, national center for biotechnology information website (NCBI, http://www.ncbi.nlm.nih.gov/gene/), using the compositionally adjusted substitution matrices [4].

Table S5. Sequences from *Diaphorina citri* that producing significant alignments with dopa decarboxylase, isoform B (GenBank Accession no. NP_724164.1) from *Drosophila melanogaster* using "*Diaphorina citri* OGS v2.0 proteins" BLAST dataset [a]

Description	Dcitr_OGSv2.0 Accession number	Length (aa)	Identities (%)	Positives (%)	Gaps (%)	E value	Score
Dopa decarboxylase	DcitrP031955.1.1	481	347/461 (75.27)	400/461 (86.77)	1/461 (0.22)	0.0	734
Aromatic-L-amino-acid decarboxylase-like Protein	DcitrP055540.1.2	588	261/475 (54.95)	351/475 (73.89)	4/475 (0.84)	0.0	566
Aromatic-L-amino-acid decarboxylase-like Protein	DcitrP055540.1.1	588	261/475 (54.95)	351/475 (73.89)	4/475 (0.84)	0.0	566
Aromatic-L-amino acid decarboxylase	DcitrP016620.1.1	759	241/513 (46.98)	334/513 (65.11)	39/513 (7.60)	4e-174	507
Aromatic-L-amino acid decarboxylase	DcitrP086355.1.1	759	241/513 (46.98)	334/513 (65.11)	39/513 (7.60)	4e-174	507
Histidine decarboxylase	DcitrP037255.1.1	726	212/413 (51.33)	289/413 (69.98)	6/413 (1.45)	5e-154	454
Histidine decarboxylase	DcitrP037255.1.1	726	68/115 (59.13)	89/115 (77.39)	3/115 (2.61)	9e-41	155
Myosin-VIIa	DcitrP031940.1.1	1586	243/439 (55.35)	291/439 (66.29)	82/439 (18.68)	5e-144	449
Myosin-VIIa	DcitrP031940.1.1	1586	60/90 (66.67)	70/90 (77.78)	0/90 (0.00)	8e-32	129
Aromatic-L-amino-acid decarboxylase-like Protein	DcitrP055670.1.1	824	181/347 (52.16)	254/347 (73.20)	4/347 (1.15)	3e-131	399
Aromatic-L-amino-acid decarboxylase-like Protein	DcitrP055670.1.1	824	122/241 (50.62)	161/241 (66.80)	4/241 (1.66)	2e-79	263
Aromatic-L-amino-acid decarboxylase-like Protein	DcitrP055670.1.1	824	33/68 (48.53)	50/68 (73.53)	0/68 (0.00)	5e-17	83.6
Glutamate decarboxylase	DcitrP073910.1.1	687	120/448 (26.79)	190/448 (42.41)	26/448 (5.80)	1e-33	134
Glutamate decarboxylase	DcitrP021460.1.2	679	106/444 (23.87)	173/444 (38.96)	80/444 (18.02)	4e-23	102
Glutamate decarboxylase	DcitrP021460.1.1	679	106/444 (23.87)	173/444 (38.96)	80/444 (18.02)	4e-23	102
Cysteine sulfinic acid decarboxylase, putative	DcitrP008780.1.1	508	82/321 (25.55)	143/321 (44.55)	26/321 (8.10)	8e-23	100

[a] The listed genes were identified using the protein-protein BLAST (BLASTp) using dopa decarboxylase, isoform B (GenBank Accession no. NP_724164.1) from *Drosophila melanogaster* [5,6], as a query sequence, against "*Diaphorina citri* OGS v2.0 proteins" BLAST datasets available on Citrus Greening Solutions website (https://citrusgreening.org/organism/Diaphorina_citri/genome).

Table S6. Sequences from *Diaphorina citri* that producing significant alignments with Arylalkylamine N-acetyltransferase 1, isoform A (AANAT1; GenBank Accession no. NP_523839.2) from *Drosophila melanogaster* using NCBI database [a]

Description	Accession number	Accession Length (aa)	Max Score	Total Score	Query Cover (%)	E value	Identity (%)
Dopamine N-acetyltransferase-like	XP_017298946.1	220	114	114	83	2e-31	34.78
Dopamine N-acetyltransferase-like isoform X1	XP_026678312.1	217	67.4	67.4	85	1e-13	30.19
Dopamine N-acetyltransferase-like - Partial	XP_017303985.2	100	67.8	67.8	40	7e-15	42.27

[a] The listed genes were identified using the protein-protein BLAST (BLASTp) using Arylalkylamine N-acetyltransferase 1, isoform A (AANAT1; GenBank Accession no. NP_523839.2) from *Drosophila melanogaster* [7–9], as a query sequence, against *Diaphorina citri* genome available in GenBank, national center for biotechnology information website (NCBI, http://www.ncbi.nlm.nih.gov/gene/), using the compositionally adjusted substitution matrices [4].

Table S7. Sequences from *Diaphorina citri* that producing significant alignments with Arylalkylamine N-acetyltransferase 1, isoform A (AANAT1; GenBank Accession no. NP_523839.2) from *Drosophila melanogaster* using "*Diaphorina citri* OGS v2.0 proteins" BLAST dataset [a]

Description	Dcitr_OGSv2.0 Accession number	Length (aa)	Identities (%)	Positives (%)	Gaps (%)	E value	Score
Dopamine N-acetyltransferase	DcitrP085745.1.1	240	86/215 40.00	119/215 55.35	7/215 3.26	2e-44	149
Dopamine N-acetyltransferase	DcitrP025630.1.1	217	64/212 30.19	94/212 44.34	10/212 4.72	7e-14	68.2

[a] The listed genes were identified using the protein-protein BLAST (BLASTp) using Arylalkylamine N-acetyltransferase 1, isoform A (AANAT1; GenBank Accession no. NP_523839.2) from *Drosophila melanogaster* [7–9], as a query sequence, against "*Diaphorina citri* OGS v2.0 proteins" BLAST datasets available on Citrus Greening Solutions website (https://citrusgreening.org/organism/Diaphorina_citri/genome).

Table S8. Sequences from *Diaphorina citri* that producing significant alignments with N-acetylserotonin O-methyltransferase-like protein (ASMT; GenBank Accession no. XP_014251646.1) from bed bug (*Cimex lectularius*) using NCBI database [a]

Description	Accession number	Accession Length (aa)	Max Score	Total Score	Query Cover (%)	E value	Identity (%)
uncharacterized protein LOC113468045	XP_026680472.1	162	153	153	68%	3e-47	50.00%
uncharacterized protein LOC103522907, partial	XP_026688590.1	103	110	110	37%	2e-31	59.09%

[a] The listed genes were identified using the protein-protein BLAST (BLASTp) using N-acetylserotonin O-methyltransferase-like protein (ASMT; GenBank Accession no. XP_014251646.1) from bed bug (*Cimex lectularius*), as a query sequence, against *Diaphorina citri* genome available in GenBank, national center for biotechnology information website (NCBI, http://www.ncbi.nlm.nih.gov/gene/), using the compositionally adjusted substitution matrices [4].

Table S9. Sequences from *Diaphorina citri* that producing significant alignments with N-acetylserotonin O-methyltransferase-like protein (ASMT; GenBank Accession no. XP_014251646.1) from bed bug (*Cimex lectularius*) using "*Diaphorina citri* OGS v2.0 proteins" BLAST dataset [a]

Description	Dcitr_OGSv2.0 Accession number	Length (aa)	Identities (%)	Positives (%)	Gaps (%)	E value	Score
N-acetylserotonin O-methyltransferase-like protein	DcitrP032285.1.1	274	108/201 (53.73)	140/201 (69.65)	2/201 (1.00)	1e-69	215

[a] The listed genes were identified using the protein-protein BLAST (BLASTp) using N-acetylserotonin O-methyltransferase-like protein (ASMT; GenBank Accession no. XP_014251646.1) from bed bug (*Cimex lectularius*), as a query sequence, against "*Diaphorina citri* OGS v2.0 proteins" BLAST datasets available on Citrus Greening Solutions website (https://citrusgreening.org/organism/Diaphorina_citri/genome).

Supplementary Figures:

```
XP_026684504.1      1 MDQISLCVPDLRLRHRENPDFFYINKNLRSSYLHSTIPSGLMLYNKHSLD
DcitrP076520.1.     1 --------------------------------------------------
consensus           1 ..................................................

XP_026684504.1     51 LDLSTAPRRFKEVCRTVLGGGRFPSGRTEEGVIFNELHKLYIKHACHEYL
DcitrP076520.1.     1 --------------------------------------------------
consensus          51 ..................................................

XP_026684504.1    101 ENWPELVRYCGYREDNIPQLEDVNTYLKRKTGFQLRPVAGYLSPRDFLSG
DcitrP076520.1.     1 --------------------------------------------------
consensus         101 ..................................................

XP_026684504.1    151 LAFRVFHCTQYIRHSSDPFYTPEPDCCHELLGHMPLLANPTFAQFSQELG
DcitrP076520.1.     1 ---------------------------------MPLLANPTFAQFSQELG
consensus         151 .................................*****************

XP_026684504.1    201 LTSLGASDEDVERLGTLYFFTVEFGLCKQDGALKVYGAGLLSSVAELTHA
DcitrP076520.1.    18 LTSLGASDEDVERLGTLYFFTVEFGLCKQDGALKVYGAGLLSSVAELTHA
consensus         201 **************************************************

XP_026684504.1    251 VTNAGDKIRRFDPAVTVSEECIITAYQNAYYYTDSFEEAKEKMREFAGTI
DcitrP076520.1.    68 VTNAGDKIRRFDPAVTVSEECIITAYQNAYYYTDSFEEAKEKMREFAGTI
consensus         251 **************************************************

XP_026684504.1    301 QRPFGVRYNPYTQSVEVLSNSEKIAALVSELRGDLCIVRNALKKIHAREE
DcitrP076520.1.   118 QRPFGVRYNPYTQSVEVLSNSEKIAALVSELRGDLCIVRNALKKIHAREE
consensus         301 **************************************************

XP_026684504.1    351 TVENLTHMLTDTMHINTQDEEEGQDISPN
DcitrP076520.1.   168 TVENLTHMLTDTMHINTQDEEEGQDISPN
consensus         351 *****************************
```

Figure S1. Multiple AA sequence alignment of *DcT5H*-1 from *Diaphorina citri*. Tryptophan 5-hydroxylase 1-like (XP_026684504.1; 379 aa) from NCBI database was aligned with Tryptophan 5-hydroxylase, putative (DcitrP076520.1.1; 196 aa), its top matched sequence from "*Diaphorina citri* OGS v2.0 proteins" BLAST datasets**.** Both genes are the top matched sequences of recent available data in GenBank, national center for biotechnology information website (NCBI, http://www.ncbi.nlm.nih.gov/gene/) and the "*Diaphorina citri* OGS v2.0 proteins" dataset available on Citrus Greening Solutions website (https://citrusgreening.org/organism/Diaphorina_citri/genome) using the Nucleotide-Nucleotide BLAST (BLASTn) and Protein-Protein BLAST (BLASTp) tools [4,10] using the NCBI tryptophan hydroxylase (GenBank Accession no. NP_612080.1) from fruit fly (*Drosophila melanogaster*) [3] as a query sequences. Conserved amino acids are indicated with black shading and those with high similarity score are in gray. The full list of genes, names, and accession numbers are available in Table 1 and supplementary Tables S2 and S3.

```
XP_017305180.1      1 ------------------------------------------------------------
DcitrP012845.1.     1 MVLNDLNPEDSYENYLLIELHLVPRFLILDSQSVVQGFDIMFNRKILVPSELDKVLVTKT
consensus           1 ............................................................

XP_017305180.1      1 ------------------------------------------------------------
DcitrP012845.1.    61 NGENGDHVVDITPTTVSSVDNKIRLRPSRNYSIVHLDSVVIELHLVPRFLILDFQSVVRG
consensus          61 ............................................................

XP_017305180.1      1 ------------------------------------------------------------
DcitrP012845.1.   121 FDIMFNRKILVPSELDKVLVTKTNGENGDHVVDITPPTLMEGGYIKEGVDSNKSTCLIFS
consensus         121 ............................................................

XP_017305180.1      1 ------------------------------------------------------------
DcitrP012845.1.   181 NHQDEVGALAKTLKLFKPTLMEGGYIKEGVDSNKSTCLIFSNHQDEVGALAKTLKLFKKN
consensus         181 ............................................................

XP_017305180.1      1 ----------------------------------------------------------MV
DcitrP012845.1.   241 NVKINDMVLIKEEHVHPLQWAMGRVTQVFPGSDGNKHNVNLLHIESRSSTRIENNYEFMV
consensus         241 ..........................................................**

XP_017305180.1      3 ECAPGGDLSSVIESLRSSCSYINIISRNHNQFHGVPWFPRRIRELDRFANQILSYGSELD
DcitrP012845.1.   301 ECAPGGDLSSVIESLRSSCSYINIISRNHNQFHGVPWFPRRIRELDRFANQILSYGSELD
consensus         301 ************************************************************

XP_017305180.1     63 ADHPGFTDPVYRARRKYFADLAFNYKHGEPLPRVDYTAEETKTWGEVFRNLTKLYPTHAC
DcitrP012845.1.   361 ADHPGFTDPVYRARRKYFADLAFNYKHGEPLPRVDYTAEETKTWGEVFRNLTKLYPTHAC
consensus         361 ************************************************************

XP_017305180.1    123 KEHNHVFPLLIQNCGYAEDNIPQLEDISKFLKDSTGFSLRPVAGLLSSRDFLAGLAFRVF
DcitrP012845.1.   421 KEHNHVFPLLIQNCGYAEDNIPQLEDISKFLKDSTGFSLRPVAGLLSSRDFLAGLAFRVF
consensus         421 ************************************************************

XP_017305180.1    183 HSTQYIRHPSKPLYTPEPDVCHELLGHVPLFADPSFAQFSQEIGLASLGAPDEYVERLAT
DcitrP012845.1.   481 HSTQYIRHPSKPLYTPEP-----------------------------------------D
consensus         481 ******************.........................................

XP_017305180.1    243 CYWFTVEFGLCRQEGQLKAFGAGLLSSFGELQYCLSDKPQRKPFEPSVTALQTYPITEYQ
DcitrP012845.1.   500 CYWFTVEFGLCRQEGQLKAFGAGLLSSFGELQYCLSDKPQRKPFEPSVTALQTYPITEYQ
consensus         541 ************************************************************

XP_017305180.1    303 PLYFVAESFEDAKDKMI-------------------------------------------
DcitrP012845.1.   560 PLYFVAESFEDAKDKMIKYAQTIPRRFGVRYNPYTQSVDVIDSKVQLSELVQNINGEMQI
consensus         601 *****************...........................................

XP_017305180.1        --------
DcitrP012845.1.   620 LLTSLKKI
consensus         661 ........
```

Figure S2. Multiple AA sequence alignment of *DcT5H*-2 from *Diaphorina citri*. Protein henna-like (XP_017305180.1; 319 aa) from NCBI database was aligned with Protein henna (DcitrP012845.1.1; 627 aa), its top matched sequence from "*Diaphorina citri* OGS v2.0 proteins" BLAST datasets. Both genes are the top matched sequences of recent available data in GenBank, national center for biotechnology information website (NCBI, http://www.ncbi.nlm.nih.gov/gene/) and the "*Diaphorina citri* OGS v2.0 proteins" dataset available on Citrus Greening Solutions website (https://citrusgreening.org/organism/Diaphorina_citri/genome) using the Nucleotide-Nucleotide BLAST (BLASTn) and Protein-Protein BLAST (BLASTp) tools [4,10] using the NCBI tryptophan hydroxylase (GenBank Accession no. NP_612080.1) from fruit fly (*Drosophila melanogaster*) [3] as a query sequences. Conserved amino acids are indicated with black shading and those with high similarity score are in gray. The full list of genes, names, and accession numbers are available in Table 1 and supplementary Tables S2 and S3.

```
XM_026828703.1      1 TCTTGCCCTCGGGTCATGGATCAGATATCTCTATGTGTTCCTGACTTGAGACTAAGACAC
DcitrC076520.1.     1 ------------------------------------------------------------
consensus           1 ............................................................

XM_026828703.1     61 AGAGAAAATCCCGACTTTTTCTATATTAACAAAAATCTGAGAAGCTCGTACCTTCACTCA
DcitrC076520.1.     1 ------------------------------------------------------------
consensus          61 ............................................................

XM_026828703.1    121 ACCATTCCATCCGGTTTAATGTTATACAACAAACATTCTCTTGATCTCGATCTTTCGACG
DcitrC076520.1.     1 ------------------------------------------------------------
consensus         121 ............................................................

XM_026828703.1    181 GCGCCGAGGAGGTTCAAGGAGGTGTGTAGGACAGTCTTGGGCGGGGGTCGTTTCCCCTCG
DcitrC076520.1.     1 ------------------------------------------------------------
consensus         181 ............................................................

XM_026828703.1    241 GGGAGAACAGAAGAGGGTGTTATATTCAACGAACTTCACAAGTTATACATCAAGCATGCA
DcitrC076520.1.     1 ------------------------------------------------------------
consensus         241 ............................................................

XM_026828703.1    301 TGTCATGAATATCTCGAGAATTGGCCGGAGTTGGTTCGTTACTGTGGCTACCGAGAAGAC
DcitrC076520.1.     1 ------------------------------------------------------------
consensus         301 ............................................................

XM_026828703.1    361 AATATTCCTCAACTAGAAGATGTGAACACCTATCTTAAGAGGAAAACTGGATTTCAGTTA
DcitrC076520.1.     1 ------------------------------------------------------------
consensus         361 ............................................................

XM_026828703.1    421 AGACCAGTAGCAGGGTATTTGTCACCCCGGGATTTTCTATCTGGTTTGGCATTTCGAGTT
DcitrC076520.1.     1 ------------------------------------------------------------
consensus         421 ............................................................

XM_026828703.1    481 TTTCATTGCACCCAGTATATTCGTCATTCATCTGACCCCTTCTATACTCCTGAACCTGAC
DcitrC076520.1.     1 ------------------------------------------------------------
consensus         481 ............................................................

XM_026828703.1    541 TGCTGTCATGAGCTACTGGGTCACATGCCTCTCCTGGCAAATCCAACATTCGCTCAGTTC
DcitrC076520.1.     1 ------------------------ATGCCTCTCCTGGCAAATCCAACATTCGCTCAGTTC
consensus         541 ........................************************************

XM_026828703.1    601 TCTCAGGAGTTGGGCCTTACTTCACTGGGAGCTTCAGATGAAGATGTGGAGAGATTGGGA
DcitrC076520.1.    37 TCTCAGGAGTTGGGCCTTACTTCACTGGGAGCTTCAGATGAAGATGTGGAGAGATTGGGA
consensus         601 ************************************************************

XM_026828703.1    661 ACGCTATACTTCTTCACAGTAGAGTTTGGTCTGTGTAAGCAAGATGGAGCACTCAAAGTG
DcitrC076520.1.    97 ACGCTATACTTCTTCACAGTAGAGTTTGGTCTGTGTAAGCAAGATGGAGCACTCAAAGTG
consensus         661 ************************************************************

XM_026828703.1    721 TATGGAGCCGGCCTTCTGTCTTCTGTCGCAGAATTAACCCATGCTGTCACGAACGCCGGT
DcitrC076520.1.   157 TATGGAGCCGGCCTTCTGTCTTCTGTCGCAGAATTAACCCATGCTGTCACGAACGCCGGT
consensus         721 ************************************************************
                                                       Forward Primer
XM_026828703.1    781 GATAAGATAAGAAGATTTGATCCAGCAGTTACTGTTAGCGAAGAATGTATCATAACTGCT
DcitrC076520.1.   217 GATAAGATAAGAAGATTTGATCCAGCAGTTACTGTTAGCGAAGAATGTATCATAACTGCT
consensus         781 ************************************************************

XM_026828703.1    841 TACCAGAATGCTTACTACTATACCGATTCGTTTGAAGAGGCTAAGGAGAAAATGAGAGAA
DcitrC076520.1.   277 TACCAGAATGCTTACTACTATACCGATTCGTTTGAAGAGGCTAAGGAGAAAATGAGAGAA
consensus         841 ************************************************************

XM_026828703.1    901 TTTGCCGGTACTATTCAACGTCCATTCGGAGTTCGTTATAACCCATACACACAGAGTGTG
DcitrC076520.1.   337 TTTGCCGGTACTATTCAACGTCCATTCGGAGTTCGTTATAACCCATACACACAGAGTGTG
consensus         901 ************************************************************

XM_026828703.1    961 GAAGTGCTGAGCAATTCGGAAAAAATTGCAGCCCTGGTGTCCGAATTGAGAGGTGACCTT
DcitrC076520.1.   397 GAAGTGCTGAGCAATTCGGAAAAAATTGCAGCCCTGGTGTCCGAATTGAGAGGTGACCTT
consensus         961 ************************************************************
                        Reverse Primer
XM_026828703.1   1021 TGCATTGTGAGGAATGCACTCAAGAAAATACATGCCCGCGAGGAAACTGTGGAGAATTTG
DcitrC076520.1.   457 TGCATTGTGAGGAATGCACTCAAGAAAATACATGCCCGCGAGGAAACTGTGGAGAATTTG
consensus        1021 ************************************************************
```

```
XM_026828703.1   1081 ACACACATGTTGACGGATACCATGCATATTAATACGCAAGACGAGGAGGAGGGTCAGGAT
DcitrC076520.1.   517 ACACACATGTTGACGGATACCATGCATATTAATACGCAAGACGAGGAGGAGGGTCAGGAT
consensus        1081 ************************************************************

XM_026828703.1   1141 ATTAGTCCGAACTGAAATAGAAAATAGAATATATTACTTTCAGCTCATATTCAGCCCATG
DcitrC076520.1.   577 ATTAGTCCGAACTGA---------------------------------------------
consensus        1141 ***************.............................................

XM_026828703.1   1201 TAGTTGGATTACAAATCATGAAATCTATCTACATACTTATATGCTATCAAATTATTACTA
DcitrC076520.1.       ------------------------------------------------------------
consensus        1201 ............................................................

XM_026828703.1   1261 AATAAATAAATAAATGCCGTTTCTTCGTGTTATCATATCGCAACGTTGGTGCTCACCATC
DcitrC076520.1.       ------------------------------------------------------------
consensus        1261 ............................................................

XM_026828703.1   1321 GTAGGATTTTTCAAATATCTCAA
DcitrC076520.1.       -----------------------
consensus        1321 .......................
```

Figure S3. Multiple nucleotide sequence alignment of *DcT5H*-1 from *Diaphorina citri*. Tryptophan 5-hydroxylase 1-like (XM_026828703.1; 1343 bp) from NCBI database was aligned with Tryptophan 5-hydroxylase, putative (DcitrC076520.1.1; 591 bp), its top matched sequence from "*Diaphorina citri* OGS v2.0 proteins" BLAST datasets**.** Both genes are the top matched sequences of recent available data in GenBank, national center for biotechnology information website (NCBI, http://www.ncbi.nlm.nih.gov/gene/) and the "*Diaphorina citri* OGS v2.0 proteins" dataset available on Citrus Greening Solutions website (https://citrusgreening.org/organism/Diaphorina_citri/genome) using the Nucleotide-Nucleotide BLAST (BLASTn) and Protein-Protein BLAST (BLASTp) tools [4,10] using the NCBI tryptophan hydroxylase (GenBank Accession no. NP_612080.1) from fruit fly (*Drosophila melanogaster*) [3] as a query sequences. Conserved amino acids are indicated with black shading and those with high similarity score are in gray. Forward and reverse primers are highlighted in yellow and blue colors, respectively. The full list of genes, names, and accession numbers are available in Table 1 and supplementary Tables S2 and S3.

```
XM_017449691.2      1 ------------------------------------------------------------
DcitrC012845.1.     1 ATTGAGTTGCACTTAGTGCCGCGGTTCCTAATACTAGACTTCCAATCCGTAGTGCGAGGG
consensus           1 ............................................................

XM_017449691.2      1 ------------------------------------------------------------
DcitrC012845.1.    61 TTCGATATAATGTTCAATAGAAAAATTCTAGTACCATCCGAACTTGATAAGGTTTTGGTT
consensus          61 ............................................................

XM_017449691.2      1 ------------------------------------------------------------
DcitrC012845.1.   121 ACGAAGACCAATGGAGAAAATGGAGATCATGTTGTGGATATTACCCCACCTACTCTCATG
consensus         121 ............................................................

XM_017449691.2      1 ------------------------------------------------------------
DcitrC012845.1.   181 GAAGGGGGCTATATCAAGGAGGGCGTGGATTCTAACAAGAGTACATGTCTGATATTCTCC
consensus         181 ............................................................

XM_017449691.2      1 ------------------------------------------------------------
DcitrC012845.1.   241 AACCACCAGGATGAAGTGGGGGCACTCGCTAAAACCCTCAAACTATTCAAGCCTACTCTC
consensus         241 ............................................................

XM_017449691.2      1 ------------------------------------------------------------
DcitrC012845.1.   301 ATGGAAGGGGGCTATATCAAGGAGGGCGTGGATTCTAACAAGAGTACATGTCTGATATTC
consensus         301 ............................................................

XM_017449691.2      1 ------------------------------------------------------------
DcitrC012845.1.   361 TCCAACCACCAGGATGAAGTGGGGGCACTCGCTAAAACCCTCAAACTATTCAAGAAGAAC
consensus         361 ............................................................

XM_017449691.2      1 ---------------------GATTTCGTGATAT-------TCTCCAACCACCAGGATGA
DcitrC012845.1.   421 AATGTCAAGATTAATGATATGGTTCTCATAAAGGAGGAGCATGTACACCCATTACAATGG
consensus         421 .....................* * **.*.* . .......* * ** ***  * .***.

XM_017449691.2     33 AGTGGGGGC------ACTCGCTAAAACCCTCAAACTATT--CAAGAAACACAACGTGAAT
DcitrC012845.1.   481 GCAATGGGCCGTGTAACTCAAGTGTTTCCTGGAAGTGACGGCAACAAACACAACGTGAAT
consensus         481 .  . ****......****.   .   *** .** *.  ..*** ***************

XM_017449691.2     85 CTGCTTCACATTGAGTCTCGCTCTTCCACACGAATTGAGAATAACTATGAGTTCATGGTA
DcitrC012845.1.   541 CTGCTTCACATCGAGTCTCGCTCTTCCACACGAATTGAGAATAACTATGAGTTCATGGTA
consensus         541 *********** ************************************************

XM_017449691.2    145 GAGTGCGCCCCTGGTGGTGACTTGTCCAGTGTCATAGAGAGTCTGAGAAGTTCCTGCTCA
DcitrC012845.1.   601 GAGTGCGCCCCTGGTGGTGACTTGTCCAGTGTCATAGAGAGTCTGAGAAGTTCCTGCTCA
consensus         601 ************************************************************

XM_017449691.2    205 TACATCAACATCATCAGCAGAAACCACAACCAATTCCATGGTGTACCCTGGTTTCCTCGT
DcitrC012845.1.   661 TACATCAACATCATCAGCAGAAACCACAACCAATTCCATGGTGTACCCTGGTTTCCACGT
consensus         661 ******************************************************** ***

XM_017449691.2    265 AGAATTCGCGAGCTGGACAGATTCGCCAATCAGATTCTATCATATGGATCCGAGCTAGAT
DcitrC012845.1.   721 AGAATTCGCGAGCTGGACAGATTCGCCAATCAGATTCTATCATATGGATCCGAGCTAGAT
consensus         721 ************************************************************

XM_017449691.2    325 GCAGATCATCCTGGATTCACAGACCCTGTGTACAGGGCGAGGAGGAAGTACTTTGCTGAC
DcitrC012845.1.   781 GCAGATCATCCTGGATTCACAGACCCTGTGTACAGGGCGAGGAGGAAGTACTTTGCTGAC
consensus         781 ************************************************************

XM_017449691.2    385 CTGGCGTTCAACTATAAACACGGAGAGCCGCTACCCCGTGTTGACTATACAGCGGAGGAA
DcitrC012845.1.   841 CTGGCGTTCAACTATAAACACGGAGAGCCGCTACCCCGTGTTGACTATACAGCGGAGGAA
consensus         841 ************************************************************
                                                  Forward Primer

XM_017449691.2    445 ACAAAAACCTGGGGTGAAGTGTTCCGGAACCTCACAAAGCTCTATCCTACTCATGCCTGT
DcitrC012845.1.   901 ACAAAAACCTGGGGTGAAGTATTCCGGAACCTCACAAAGCTCTATCCTACTCATGCCTGT
consensus         901 ********************.***************************************

XM_017449691.2    505 AAGGAACACAACCACGTGTTTCCGCTTCTAATACAGAACTGTGGCTATGCAGAGGACAAT
DcitrC012845.1.   961 AAGGAACACAACCACGTGTTTCCGCTTCTAATACAGAACTGTGGCTATGCAGAGGACAAT
consensus         961 ************************************************************

XM_017449691.2    565 ATTCCTCAGCTGGAGGACATCTCCAAATTCTTGAAAGATAGTACAGGGTTCTCCTTGCGC
DcitrC012845.1.  1021 ATTCCTCAGCTGGAGGACATCTCCAAATTCTTGAAAGATAGTACAGGGTTCTCCTTGCGC
consensus        1021 ************************************************************
```

```
XM_017449691.2    625 CCAGTAGCAGGTCTGCTCTCATCACGTGACTTCCTAGCAGGTCTTGCGTTCCGTGTCTTT
DcitrC012845.1.  1081 CCAGTAGCAGGTCTGCTCTCATCACGTGACTTCCTAGCAGGTCTTGCGTTCCGTGTCTTT
consensus        1081 ************************************************************
                               Reverse Primer

XM_017449691.2    685 CACTCGACCCAGTATATTCGACATCCCAGCAAGCCCCTGTATACTCCGGAGCCTGACGTT
DcitrC012845.1.  1141 CACTCGACCCAGTATATTCGACATCCCAGCAAGCCCCTGTATACTCCGGAGCCTGAC---
consensus        1141 *********************************************************...

XM_017449691.2    745 TGTCATGAACTATTAGGTCATGTGCCACTGTTTGCTGATCCTAGTTTCGCTCAGTTCTCT
DcitrC012845.1.  1198 ------------------------------------------------------------
consensus        1201 ............................................................

XM_017449691.2    805 CAAGAGATCGGTCTCGCTTCGCTAGGAGCTCCGGATGAATATGTGGAGCGACTCGCTACG
DcitrC012845.1.  1198 ------------------------------------------------------------
consensus        1261 ............................................................

XM_017449691.2    865 TGCTATTGGTTCACGGTAGAGTTCGGGCTATGCCGTCAGGAAGGTCAACTCAAAGCATTC
DcitrC012845.1.  1198 TGCTATTGGTTCACGGTAGAGTTCGGGCTATGCCGTCAGGAAGGTCAACTCAAAGCATTC
consensus        1321 ************************************************************

XM_017449691.2    925 GGTGCCGGACTGCTCTCCTCATTTGGTGAACTGCAATACTGTCTCTCCGACAAACCACAG
DcitrC012845.1.  1258 GGTGCCGGACTGCTCTCCTCATTTGGTGAACTGCAATACTGTCTCTCCGACAAACCACAG
consensus        1381 ************************************************************

XM_017449691.2    985 CGCAAACCCTTCGAACCCTCGGTCACTGCATTGCAAACCTACCCCATCACCGAGTACCAG
DcitrC012845.1.  1318 CGCAAACCCTTCGAACCCTCGGTCACTGCATTGCAAACCTACCCCATCACCGAGTACCAG
consensus        1441 ************************************************************

XM_017449691.2   1045 CCCTTGTACTTTGTGGCTGAGAGCTTTGAGGATGCCAAGGACAAAATGATCTAATACGCG
DcitrC012845.1.  1378 CCCTTGTACTTTGTGGCTGAGAGCTTTGAGGATGCCAAGGACAAAATGATCAAATACGCG
consensus        1501 *************************************************** ********

XM_017449691.2   1105 CAGACCATCCCCCGTAGGTTTGGAGTGCGCTATAATCCATACACTCAGAGTGTTGATGTG
DcitrC012845.1.  1438 CAGACCATCCCCCGTAGGTTTGGAGTGCGCTATAATCCATACACTCAGAGTGTTGATGTG
consensus        1561 ************************************************************

XM_017449691.2   1165 ATTGATTCCAAGGTTCAACTTAGTGAGCTGGTGCAGAACATCAACGGGGAAATGCAGATC
DcitrC012845.1.  1498 ATTGATTCCAAGGTTCAACTTAGTGAGCTGGTGCAAAATATTAACGGGGAAATGCAGATC
consensus        1621 ***********************************.** ** ******************

XM_017449691.2   1225 CTGCTGACCTCACTCAAGAAAATCTAAGCAACGCCGAGCCTGGCTTGCAACACAACGGGT
DcitrC012845.1.  1558 CTGCTGACCTCACTCAAGAAAATCTAA---------------------------------
consensus        1681 ***************************.................................

XM_017449691.2   1285 GACCGTCTGGACAC
DcitrC012845.1.       --------------
consensus        1741 ..............
```

Figure S4. Multiple nucleotide sequence alignment of *DcT5H*-2 from *Diaphorina citri*. Protein henna-like (XM_017449691.2; 1298 bp) from NCBI database was aligned with Protein henna (DcitrC012845.1.1; 1884 bp), its top matched sequence from "*Diaphorina citri* OGS v2.0 proteins" BLAST datasets. Both genes are the top matched sequences of recent available data in GenBank, national center for biotechnology information website (NCBI, http://www.ncbi.nlm.nih.gov/gene/) and the "*Diaphorina citri* OGS v2.0 proteins" dataset available on Citrus Greening Solutions website (https://citrusgreening.org/organism/Diaphorina_citri/genome) using the Nucleotide-Nucleotide BLAST (BLASTn) and Protein-Protein BLAST (BLASTp) tools [4,10] using the NCBI tryptophan hydroxylase (GenBank Accession no. NP_612080.1) from fruit fly (*Drosophila melanogaster*) [3] as a query sequences. Conserved amino acids are indicated with black shading and those with high similarity score are in gray. Forward and reverse primers are highlighted in yellow and blue colors, respectively. The full list of genes, names, and accession numbers are available in Table 1 and supplementary Tables S2 and S3.

```
XP_008484302.1      1 MGDVNEFKDFAKAMVDYVGNYLENIRDRRVLPTVEPGYLRPLIPETAPDTPDTWQEVMSD
DcitrP031955.1.     1 MGDVNEFKDFAKAMVDYVGNYLENIRDRRVLPTVEPGYLRPLIPETAPDTPDTWQEVMSD
XP_017300015.1      1 MGDVNEFKDFAKAMVDYVGNYLENIRDRRVLPTVEPGYLRPLIPETAPDTPDTWQEVMSD
consensus           1 ************************************************************

XP_008484302.1     61 IERVIMPGVTHWHSPKFHAYFPTANSYPAIVADILSDSIACIGFTWIASPACTELEVVML
DcitrP031955.1.    61 IERVIMPGVTHWHSPKFHAYFPTANSYPAIVADILSDSIACIGFTWIASPACTELEVVML
XP_017300015.1     61 IERVIMPGVTHWHSPKFHAYFPTANSYPAIVADILSDSIACIGFTWIASPACTELEVVML
consensus          61 ************************************************************

XP_008484302.1    121 DWLGKMLDLPKEFLACSGGKGGGVIQGTASEATLVALLGAKAKTMQRVKEAHPDWKDSDI
DcitrP031955.1.   121 DWLGKMLDLPKEFLACSGGKGGGVIQGTASEATLVALLGAKAKTMQRVKEAHPDWKDSDI
XP_017300015.1    121 DWLGKMLDLPKEFLACSGGKGGGVIQGTASEATLVALLGAKAKTMQRVKEAHPDWKDSDI
consensus         121 ************************************************************

XP_008484302.1    181 IANLVGYCSDQAHSSVERAGLLGGVTIRGLPADDS---YKLRGDALEAAIEEDLKKGKIP
DcitrP031955.1.   181 IANLVGYCSDQAHSSVERAGLLGGVTIRGLPADDS---YKLRGDALEAAIEEDLKKGKIP
XP_017300015.1    181 IANLVGYCSGKHSTTGHAGLAGGVVPLRRFKPNTPQSKKLCSAFICPENIEEDLKKGKIP
consensus         181 *********.............*.*..*.......   ...........***********

XP_008484302.1    238 FYVVATLGTTNCCAFDCLEEIGQVCRQKDVWLHVDAAYAGSAFICPENRYLMKGVELADS
DcitrP031955.1.   238 FYVVATLGTTNCCAFDCLEEIGQVCRQKDVWLHVDAAYAGSAFICPENRYLMKGVELADS
XP_017300015.1    241 FYVVATLGTTNCCAFDCLEEIGQVCRQKDVWLHVDAAYAGSAFICPENRYLMKGVELADS
consensus         241 ************************************************************

XP_008484302.1    298 FNFNPHKWMLVTFDCSAMWLKDPSWVVNAFNVDPLYLKHDQQGSAPDYRHWQIPLGRRFR
DcitrP031955.1.   298 FNFNPHKWMLVTFDCSAMWLKDPSWVVNAFNVDPLYLKHDQQGSAPDYRHWQIPLGRRFR
XP_017300015.1    301 FNFNPHKWMLVTFDCSAMWLKDPSWVVNAFNVDPLYLKHDQQGSAPDYRHWQIPLGRRFR
consensus         301 ************************************************************

XP_008484302.1    358 SLKLWFVLRLLGVKYLQEYIRKQISLAKEFEQLVRSDDRFEVIYEVLMGLVCFRLKGPNE
DcitrP031955.1.   358 SLKLWFVLRLLGVKYLQEYIRKQISLAKEFEQLVRSDDRFEVIYEVLMGLVCFRLKGPNE
XP_017300015.1    361 SLKLWFVLRLLGVKYLQEYIRKQISLAKEFEQLVRSDDRFEVIYEVLMGLVCFRLKGPNE
consensus         361 ************************************************************

XP_008484302.1    418 INEKLLKRINGNGKIHLVPSKIRDVYFLRMAVCSRYSESSDMIYSWNEIKTLTEELLKEE
DcitrP031955.1.   418 INEKLLKRINGNGKIHLVPSKIRDVYFLRMAVCSRYSESSDMIYSWNEIKTLTEELLKEE
XP_017300015.1    421 INEKLLKRINGNGKIHLVPSKIRDVYFLRMAVCSRYSESSDMIYSWNEIKTLTEELLKEE
consensus         421 ************************************************************

XP_008484302.1    478 KEKA
DcitrP031955.1.   478 KEKA
XP_017300015.1    481 KEKA
consensus         481 ****
```

Figure S5. Multiple AA sequence alignment of *DcAADC* from *Diaphorina citri*. Aromatic-L-amino-acid decarboxylase (XP_008484302.1; 481 aa and XP_017300015.1; 484 aa) from NCBI database were aligned with Dopa decarboxylase (DcitrP031955.1.1; 481 aa), its top matched sequence from "*Diaphorina citri* OGS v2.0 proteins" BLAST datasets. Both genes are the top matched sequences of recent available data in GenBank, national center for biotechnology information website (NCBI, http://www.ncbi.nlm.nih.gov/gene/) and the "*Diaphorina citri* OGS v2.0 proteins" dataset available on Citrus Greening Solutions website (https://citrusgreening.org/organism/Diaphorina_citri/genome) using the Nucleotide-Nucleotide BLAST (BLASTn) and Protein-Protein BLAST (BLASTp) tools [4,10] using the NCBI dopa decarboxylase - isoform B (GenBank Accession no. NP_724164.1) from fruit fly (*D. melanogaster*) [5,6] as a query sequences. Conserved amino acids are indicated with black shading and those with high similarity score are in gray. The full list of genes, names, and accession numbers are available in Table 1 and supplementary Tables S4 and S5.

```
XM_008486080.3      1 ------------------------------------------------------------
DcitrC031955.1.     1 ------------------------------------------------------------
XM_017444526.2      1 CAAAATTTCAAATTACTACCAGTTAACTCTTCAAGAAGTCAACTCTTGAGGAATTTTTGT
consensus           1 

XM_008486080.3      1 ------------------------------------------------------------
DcitrC031955.1.     1 ------------------------------------------------------------
XM_017444526.2     61 TCGAGTCACCCGCATTTTAGTTTGAGTCATTTCGGGGAAGGAACAAAAACACCGTATTTA
consensus          61 

XM_008486080.3      1 ------------------------------------------------------------
DcitrC031955.1.     1 ------------------------------------------------------------
XM_017444526.2    121 TTTATTTAAATCATTTATTTATTTTTTATTAATTGACATTGCCGATTTTTTTTTTCGTGT
consensus         121 

XM_008486080.3      1 ------------------------------------------------------------
DcitrC031955.1.     1 ------------------------------------------------------------
XM_017444526.2    181 TTACTTAATTTATTCACGAACTCGTTTTGTTTGTTGTTAATTAATTTGGATATTACTTAA
consensus         181 

XM_008486080.3      1 CCAGGGCTCCAACATCATGGGAGACGTGAACGAATTCAAAGACTTTGCCAAAGCCATGGT
DcitrC031955.1.     1 ----------------ATGGGAGACGTGAACGAATTCAAAGACTTTGCCAAAGCCATGGT
XM_017444526.2    241 TCAGGGCTCCATCATCATGGGAGACGTGAATGAATTCAAAGACTTTGCCAAAGCCATGGT
consensus         241  .......... ....**************.*****************************

XM_008486080.3     61 GGACTATGTCGGAAACTACTTGGAAAATATTCGAGATAGACGCGTTTTGCCAACCGTCGA
DcitrC031955.1.    45 GGACTATGTCGGAAACTACTTGGAAAATATTCGAGATAGACGCGTTTTACCGACCGTCGA
XM_017444526.2    301 GGACTATGTCGGAAACTACTTGGAAAATATTCGAGATAGACGCGTTTTGCCAACCGTCGA
consensus         301 ************************************************.**.********

XM_008486080.3    121 GCCCGGCTATCTGCGACCCCTCATCCCTGAGACCGCCCCTGACACTCCGGACACCTGGCA
DcitrC031955.1.   105 GCCCGGCTATCTGCGACCCCTCATCCCTGAGACCGCCCCTGACACTCCGGACACCTGGCA
XM_017444526.2    361 GCCCGGCTATCTGCGACCCCTCATCCCTGAGACCGCCCCTGACACTCCGGACACCTGGCA
consensus         361 ************************************************************

XM_008486080.3    181 AGAAGTCATGAGTGACATTGAACGGGTCATCATGCCCGGAGTCACCCATTGGCACTCACC
DcitrC031955.1.   165 AGAAGTCATGAGTGACATTGAACGGGTCATCATGCCCGGAGTCACCCACTGGCACTCACC
XM_017444526.2    421 AGAAGTCATGAGTGACATTGAACGGGTCATCATGCCCGGAGTCACCCATTGGCACTCACC
consensus         421 ************************************************.***********

XM_008486080.3    241 CAAATTCCATGCCTACTTCCCCCACAGCCAACTCCTATCCCGCCATCGTAGCCGATATCCT
DcitrC031955.1.   225 CAAGTTCCATGCCTACTTCCCCCACAGCCAACTCCTATCCCGCCATCGTAGCCGATATCCT
XM_017444526.2    481 CAAATTCCATGCCTACTTCCCCCACAGCCAACTCCTATCCCGCCATCGTAGCCGATATCCT
consensus         481 ***.********************************************************

XM_008486080.3    301 GAGTGACTCTATCGCCTGCATAGGATTCACATGGATTGCCAGTCCTGCTTGCACAGAGTT
DcitrC031955.1.   285 GAGTGACTCTATCGCCTGCATAGGATTCACATGGATTGCCAGTCCTGCTTGCACAGAGTT
XM_017444526.2    541 GAGTGACTCTATCGCCTGCATAGGATTCACATGGATTGCCAGTCCTGCTTGCACAGAGTT
consensus         541 ************************************************************

XM_008486080.3    361 GGAAGTAGTCATGCTGGATTGGCTGGGCAAGATGTTGGATCTGCCCAAGGAATTCTTAGC
DcitrC031955.1.   345 GGAAGTAGTCATGCTGGATTGGCTGGGCAAGATGTTGGATCTGCCCAAGGAATTCTTAGC
XM_017444526.2    601 GGAAGTAGTCATGCTGGATTGGCTGGGCAAGATGTTGGATCTGCCCAAGGAATTCTTAGC
consensus         601 ************************************************************

XM_008486080.3    421 CTGCTCTGGAGGAAAGGGAGGTGGCGTTATTCAGGGTACAGCTAGTGAAGCTACGCTAGT
DcitrC031955.1.   405 CTGCTCTGGAGGAAAGGGAGGTGGCGTTATTCAGGGTACAGCTAGTGAAGCTACGCTAGT
XM_017444526.2    661 CTGCTCTGGAGGAAAGGGAGGTGGCGTTATTCAGGGTACAGCTAGTGAAGCTACGCTAGT
consensus         661 ************************************************************

XM_008486080.3    481 AGCTCTGCTCGGAGCTAAGGCCAAAACCATGCAGAGAGTCAAAGAGGCTCATCCAGACTG
DcitrC031955.1.   465 AGCTCTGCTCGGAGCTAAGGCCAAAACCATGCAGAGAGTCAAAGAGGCTCATCCAGACTG
XM_017444526.2    721 AGCTCTGCTCGGAGCTAAGGCCAAAACCATGCAGAGAGTCAAAGAGGCTCATCCAGACTG
consensus         721 ************************************************************

XM_008486080.3    541 GAAAGACTCAGACATTATTGCCAACTTGGTCGGATACTGCTCAGATCAAGCTCATTCGTC
DcitrC031955.1.   525 GAAAGATTCCGACATCATTGCCAACCTGGTCGGATACTGCTCAGATCAAGCTCATTCGTC
XM_017444526.2    781 GAAAGATTCCGACATCATTGCCAACCTGGTCGGATACTGCTCAGGTAAG---CACTCAAC
consensus         781 ******.**.*****.*********.******************.*.*....**.**..*
```

```
XM_008486080.3    601 CGTGGAGCGTGCTGGCCTCCTAGGAGGAGTT---ACTATTAGGGGACTCCCAGCTGACG-
DcitrC031955.1.   585 CGTAGAGCGTGCTGGTCTCCTAGGAGGAGTT---ACTATTAGGGGACTCCCAGCTGACG-
XM_017444526.2    838 TACAGGTCATGCTGGATTGGCTGGCGGAGTTGTGCCTCTCCGGAGATTCAAACCGAACAC
consensus         841 ....*..*.******  .*....**.******    .**.*..**.**.**..*.*..**.

XM_008486080.3    657 -----ACAGCTACAAACTGAGAGGTGATGCTTTGG---AAGCGGCTATCGAGGAAGACCT
DcitrC031955.1.   641 -----ACAGCTACAAACTGAGAGGTGACGCTTTGG---AAGCGGCCATCGAGGAAGACTT
XM_017444526.2    898 GCCTCAGAGCAAAAAACTCTGTTCTGCGTTTATCTGCCCAGAAAACATCGAGGAAGACTT
consensus         901      *.***.*.*****..*...**.  ..*.*..    .**.....************.*

XM_008486080.3    709 GAAGAAGGGAAAGATCCCATTTTACGTGGTGGCAACTCTGGGAACAACCAACTGTTGTGC
DcitrC031955.1.   693 GAAGAAGGGAAAGATCCCATTTTACGTGGTGGCAACTCTGGGAACAACCAACTGTTGTGC
XM_017444526.2    958 GAAGAAGGGAAAGATCCCATTTTACGTGGTGGCAACTCTGGGAACAACCAACTGTTGTGC
consensus         961 ************************************************************

XM_008486080.3    769 TTTCGATTGTTTAGAGGAAATCGGACAAGTGTGTAGACAGAAAGATGTGTGGCTTCATGT
DcitrC031955.1.   753 TTTTGATTGTTTAGAGGAAATCGGCCAAGTGTGTAGACAGAAAGATGTGTGGCTTCATGT
XM_017444526.2   1018 TTTTGATTGTTTAGAGGAAATCGGCCAAGTGTGTAGACAGAAAGATGTGTGGCTTCATGT
consensus        1021 ***.********************.***********************************

XM_008486080.3    829 GGACGCGGCCTATGCTGGTTCTGCGTTTATCTGCCCAGAAAATCGTTATCTCATGAAAGG
DcitrC031955.1.   813 GGACGCGGCCTATGCTGGTTCTGCGTTTATCTGCCCAGAAAATCGTTATCTCATGAAAGG
XM_017444526.2   1078 GGACGCGGCCTATGCTGGTTCTGCGTTTATCTGCCCAGAAAATCGTTATCTCATGAAAGG
consensus        1081 ************************************************************

XM_008486080.3    889 TGTGGAGCTGGCAGATTCCTTCAATTTCAATCCACACAAATGGATGCTGGTTACATTCGA
DcitrC031955.1.   873 TGTTGAGCTGGCAGATTCCTTCAATTTCAATCCACACAAATGGATGCTGGTTACATTCGA
XM_017444526.2   1138 TGTGGAGCTGGCAGATTCCTTCAATTTCAATCCACACAAATGGATGCTGGTTACATTCGA
consensus        1141 ***.********************************************************

XM_008486080.3    949 CTGCTCTGCTATGTGGTTGAAGGATCCTTCTTGGGTTGTCAATGCGTTCAATGTGGATCC
DcitrC031955.1.   933 CTGCTCTGCTATGTGGTTGAAGGATCCTTCTTGGGTTGTCAATGCGTTCAATGTGGATCC
XM_017444526.2   1198 CTGCTCTGCTATGTGGTTGAAGGATCCTTCTTGGGTTGTCAATGCGTTCAATGTGGATCC
consensus        1201 ************************************************************

XM_008486080.3   1009 CCTTTATCTGAAGCACGACCAGCAAGGATCTGCTCCCGATTACAGGCATTGGCAAATACC
DcitrC031955.1.   993 CCTTTATCTGAAGCACGACCAGCAAGGATCTGCTCCCGATTACAGGCATTGGCAAATACC
XM_017444526.2   1258 CCTTTATCTGAAGCACGACCAGCAAGGATCTGCTCCCGATTACAGGCATTGGCAAATACC
consensus        1261 ************************************************************

XM_008486080.3   1069 ACTAGGCCGTAGGTTCAGATCCCTCAAGCTGTGGTTCGTACTTCGTCTCCTGGGAGTGAA
DcitrC031955.1.  1053 ACTAGGCCGTAGGTTCAGATCCCTCAAGCTGTGGTTCGTACTTCGTCTCCTGGGAGTGAA
XM_017444526.2   1318 ACTAGGCCGTAGGTTCAGATCCCTCAAGCTGTGGTTCGTACTTCGTCTCCTGGGAGTGAA
consensus        1321 ************************************************************
                            Forward Primer

XM_008486080.3   1129 ATATCTTCAGGAGTACATCCGGAAACAGATCAGCCTGGCTAAGGAATTTGAACAGCTGGT
DcitrC031955.1.  1113 ATATCTTCAGGAGTACATCCGGAAACAGATCAGCCTGGCTAAGGAATTTGAACAGCTGGT
XM_017444526.2   1378 ATATCTTCAGGAGTACATCCGGAAACAGATCAGCCTGGCTAAGGAATTTGAACAGCTGGT
consensus        1381 ************************************************************

XM_008486080.3   1189 CAGATCGGATGACAGGTTTGAGGTCATCTATGAGGTGCTCATGGGACTGGTGTGTTTCCG
DcitrC031955.1.  1173 CAGATCGGATGACAGGTTTGAGGTCATCTATGAGGTGCTCATGGGACTGGTGTGTTTCCG
XM_017444526.2   1438 CAGATCGGATGACAGGTTTGAGGTCATCTATGAGGTGCTCATGGGACTGGTGTGTTTCCG
consensus        1441 ************************************************************
                                                                 Reverse Primer

XM_008486080.3   1249 ACTCAAGGGACCAAACGAGATCAACGAGAAACTTCTCAAACGTATCAACGGGAACGGCAA
DcitrC031955.1.  1233 ACTCAAGGGACCAAACGAGATTAACGAGAAACTTCTCAAACGTATCAACGGGAACGGCAA
XM_017444526.2   1498 ACTCAAGGGACCAAACGAGATCAACGAGAAACTTCTCAAACGTATCAACGGGAACGGCAA
consensus        1501 *********************.**************************************

XM_008486080.3   1309 GATCCATCTAGTGCCGTCGAAAATCCGTGATGTCTATTTCCTGCGTATGGCCGTATGCAG
DcitrC031955.1.  1293 GATCCATCTAGTGCCGTCGAAAATCCGTGATGTCTATTTCCTGCGTATGGCCGTATGCAG
XM_017444526.2   1558 GATCCATCTAGTGCCGTCGAAAATCCGTGATGTCTATTTCCTGCGTATGGCCGTATGCAG
consensus        1561 ************************************************************

XM_008486080.3   1369 TCGTTACAGCGAATCTTCAGATATGATTTACTCGTGGAACGAAATCAAAACTCTCACCGA
DcitrC031955.1.  1353 TCGTTACAGCGAATCTTCAGATATGATTTACTCGTGGAACGAAATCAAAACTCTCACCGA
XM_017444526.2   1618 TCGTTACAGCGAATCTTCAGATATGATTTACTCGTGGAACGAAATCAAAACTCTCACCGA
consensus        1621 ************************************************************
```

```
XM_008486080.3  1429 AGAACTACTCAAGGAGGAAAAGGAAAAAGCTTGAACTTTCAACTGGTCGAGGAAAGCGCT
DcitrC031955.1. 1413 AGAACTACTCAAGGAGGAAAAGGAAAAAGCTTGA--------------------------
XM_017444526.2  1678 AGAACTACTCAAGGAGGAAAAGGAAAAAGCTTGAACTTTCAACTGGTCGAGGAAAGCGCT
consensus       1681 **********************************..........................

XM_008486080.3  1489 GAGCTTCCTGTTAGAGAAATTAAATTGACTCTCATCGTTGGTATTTATAAGGATTTTCCC
DcitrC031955.1.      ------------------------------------------------------------
XM_017444526.2  1738 ------------------------------------------------------------
consensus       1741

XM_008486080.3  1549 CCGCTTTACTCTATGTTAAATTTTATGGTCCACCGTGCTAACCACTACACCTCTGAGGTT
DcitrC031955.1.      ------------------------------------------------------------
XM_017444526.2       ------------------------------------------------------------
consensus       1801

XM_008486080.3  1609 GGTCATTTTTGGTGTTTATTTCATCACTGCGCGGAAAAAGAACTCAAATAACTTAATCAA
DcitrC031955.1.      ------------------------------------------------------------
XM_017444526.2       ------------------------------------------------------------
consensus       1861

XM_008486080.3  1669 ATGGGTTCTCGTACTTCTCTTATAGACACTTGATCCAAAATGACACTATTCATGTTATAT
DcitrC031955.1.      ------------------------------------------------------------
XM_017444526.2       ------------------------------------------------------------
consensus       1921

XM_008486080.3  1729 TTCTGTCAAGAACTCTGATTGAAAGAGAAAATTTCAGTTTCGAGATGGACCTTTATAGGT
DcitrC031955.1.      ------------------------------------------------------------
XM_017444526.2       ------------------------------------------------------------
consensus       1981

XM_008486080.3  1789 AGTTAGGTTGCTATACAAGGAAATTTTGCAACAAAAATTCAGAGTGAAATTTCCATTCAT
DcitrC031955.1.      ------------------------------------------------------------
XM_017444526.2       ------------------------------------------------------------
consensus       2041

XM_008486080.3  1849 AAGATTAGTTTTTCTGTTGTTTTGTTATTCTCTTCGTGTAAATCATGATTATGTGTAATG
DcitrC031955.1.      ------------------------------------------------------------
XM_017444526.2       ------------------------------------------------------------
consensus       2101

XM_008486080.3  1909 TTGTCATTAAAAACAATCCGTAATAATTTAT
DcitrC031955.1.      -------------------------------
XM_017444526.2       -------------------------------
consensus       2161
```

Figure S6. Multiple nucleotide sequence alignment of *DcT5H*-1 from *Diaphorina citri*. Aromatic-L-amino-acid decarboxylase (XM_008486080.3; 1939 bp and XM_017444526.2; 1737 bp) from NCBI database were aligned with Dopa decarboxylase (DcitrC031955.1.1; 1446 bp), its top matched sequence from "*Diaphorina citri* OGS v2.0 proteins" BLAST datasets. Both genes are the top matched sequences of recent available data in GenBank, national center for biotechnology information website (NCBI, http://www.ncbi.nlm.nih.gov/gene/) and the "*Diaphorina citri* OGS v2.0 proteins" dataset available on Citrus Greening Solutions website (https://citrusgreening.org/organism/Diaphorina_citri/genome) using the Nucleotide-Nucleotide BLAST (BLASTn) and Protein-Protein BLAST (BLASTp) tools [4,10] using the NCBI-dopa decarboxylase - isoform B (GenBank Accession no. NP_724164.1) from fruit fly (*D. melanogaster*) [5,6] as a query sequences. Conserved amino acids are indicated with black shading and those with high similarity score are in gray. Forward and reverse primers are highlighted in yellow and blue colors, respectively. The full list of genes, names, and accession numbers are available in Table 1 and supplementary Tables S2 and S3.

```
XP_026678312.1      1 MDYKIEKITKNDEPLVNQFLRDNFYTSEPLNVAFRAKFNATESLAANSNFENIANGLSLK
DcitrP025630.1.     1 MDYKIEKITKNDEPLVNQFLRDNFYTSEPLNVAFRAKFNATESLAANSNFENIANGLSLK
consensus           1 ************************************************************

XP_026678312.1     61 AVDGDGNILGVSLNTCPLEGRHIIVRKEIEGRREIMEEIIKFLGLIDELSGVEKLIEKHH
DcitrP025630.1.    61 AVDGDGNILGVSLNTCPLEGRHIIVRKEIEGRREIMEEIIKFLGLIDELSGVEKLIEKHH
consensus          61 ************************************************************

XP_026678312.1    121 QSVLDVKILSVDAKHRQKGIGARLIEETIKTAEAHDIKAITVGCSSYYTSVIVQHKFPNQ
DcitrP025630.1.   121 QSVLDVKILSVDAKHRQKGIGARLIEETIKTAEAHDIKAITVGCSSYYTSMIVQHKFPNQ
consensus         121 **************************************************.*********

XP_026678312.1    181 FRRVLTYPYSKYVDSNGEVVFKSAEPHVEYHVYFYER
DcitrP025630.1.   181 FRRVLTYPYSKYVDSKGEVVFKSAEPHVEYHVYFYER
consensus         181 *************** *********************
```

Figure S7. Multiple AA sequence alignment of *DcAANAT*-1 from *Diaphorina citri*. Dopamine N-acetyltransferase-like isoform X1 (XP_026678312.1; 217 aa) from NCBI database was aligned with Dopamine N-acetyltransferase (DcitrP025630.1.1; 217 aa), its top matched sequence from "*Diaphorina citri* OGS v2.0 proteins" BLAST datasets**.** Both genes are the top matched sequences of recent available data in GenBank, national center for biotechnology information website (NCBI, http://www.ncbi.nlm.nih.gov/gene/) and the "*Diaphorina citri* OGS v2.0 proteins" dataset available on Citrus Greening Solutions website (https://citrusgreening.org/organism/Diaphorina_citri/genome) using the Nucleotide-Nucleotide BLAST (BLASTn) and Protein-Protein BLAST (BLASTp) tools [4,10] using the NCBI arylalkylamine N-acetyltransferase 1, isoform A (GenBank Accession no. NP_523839.2) from fruit fly (*D. melanogaster*) [7–9] as a query sequences. Conserved amino acids are indicated with black shading and those with high similarity score are in gray. The full list of genes, names, and accession numbers are available in Table 1 and supplementary Tables S6 and S7.

```
XP_017298946.1      1 --------MSKEEIDYVYPIPEDKYNDVIEHLRYNFFADEPLNKCVGLCETGRGHSELEL
DcitrP085745.1.     1 METTAPPTATMLNYSIVRLSPSDKD-RVIAFLVKFFFHDEPLNIAVGLMQDGQRCIELED
consensus           1 ........ .      *   * ** . **  *   ** *****  ***   *    ***

XP_017298946.1     53 HSILTLQDNLSVMAVNGNGQVIGVALNGIQH--EGDVDEAIKKLETLNDKKFKQIFSMLY
DcitrP085745.1.    60 YCTTPLAQGLSLAAVNERQEIVGVCINTINYRRESSTGPPESGEDECAHPKFKIILKFLK
consensus          61      *   **. ***    ..** .* *. ..*          .     *** *   *

XP_017298946.1    111 DLNQSLNLFSRYDVTSIFECRILSVDNNYRGRGLANELFKLSIDIASKAGFKVFKVDATG
DcitrP085745.1.   120 WLDKKNDIFSKFNINKYLDISILSTDSAYRGQGIAKKLVYESIDLAADNDIHLVKADCSS
consensus         121  *     .**.. .    .  *** * *** *.*  *   ***.*     .. * * .

XP_017298946.1    171 VFSQKISTKLGLETLIELEYRNHLDSATGLPMFTPPSPHTSLKVMVKILQ----------
DcitrP085745.1.   180 HYSAKAMARLGFSCIYSMSYEDYTGSDN-KPVFSPPAPHHGAETYVLDVKRFLEQRPNIL
consensus         181  .* *   .**   .  . *     *  . *.*.** **      *  . ..........

XP_017298946.1        --
DcitrP085745.1.   239 SA
consensus         241 ..
```

Figure S8. Multiple AA sequence alignment of *DcAANAT*-2 from *Diaphorina citri*. Dopamine N-acetyltransferase-like (XP_017298946.1; 220 aa) from NCBI database was aligned with Dopamine N-acetyltransferase (DcitrP085745.1.1; 240 aa), its top matched sequence from "*Diaphorina citri* OGS v2.0 proteins" BLAST datasets. Both genes are the top matched sequences of recent available data in GenBank, national center for biotechnology information website (NCBI, http://www.ncbi.nlm.nih.gov/gene/) and the "*Diaphorina citri* OGS v2.0 proteins" dataset available on Citrus Greening Solutions website (https://citrusgreening.org/organism/Diaphorina_citri/genome) using the Nucleotide-Nucleotide BLAST (BLASTn) and Protein-Protein BLAST (BLASTp) tools [4,10] using the NCBI arylalkylamine N-acetyltransferase 1, isoform A (GenBank Accession no. NP_523839.2) from fruit fly (*D. melanogaster*) [7–9] as a query sequences. Conserved amino acids are indicated with black shading and those with high similarity score are in gray. The full list of genes, names, and accession numbers are available in Table 1 and supplementary Tables S6 and S7.

```
XM_026822511.1      1 GGGATATTATCCCGCATTGAGTCTCACTTTAGTTACATCCAGTGGTGAAACAAATTCCTT
DcitrC025630.1.     1 ------------------------------------------------------------
consensus           1 ............................................................

XM_026822511.1     61 TGTCCGGTGACCAGTCAGGAAATTACTTCAGGAGAGGCGAAACGTCATACCAGAATCAGT
DcitrC025630.1.     1 ------------------------------------------------------------
consensus          61 ............................................................

XM_026822511.1    121 CAACATCTACTTCAGGAAAGGTGGGACCTAGCGGCAGACCAGATCGGTCACTCACAGAAT
DcitrC025630.1.     1 ----------------------------------------------------------AT
consensus         121 ..........................................................**

XM_026822511.1    181 GGACTACAAAATCGAAAAAATCACCAAAAACGACGAACCACTCGTGAACCAATTCCTCCG
DcitrC025630.1.     3 GGACTACAAAATCGAAAAAATCACCAAAAACGACGAACCACTCGTGAACCAATTCCTCCG
consensus         181 ************************************************************

XM_026822511.1    241 CGACAATTTCTACACCTCAGAACCGCTCAATGTCGCTTTCCGAGCGAAATTCAATGCCAC
DcitrC025630.1.    63 CGACAATTTCTACACCTCAGAACCGCTCAATGTCGCTTTCCGAGCGAAATTCAATGCCAC
consensus         241 ************************************************************

XM_026822511.1    301 CGAATCACTCGCTGCCAATTCCAACTTTGAAAACATCGCCAACGGCCTCAGTCTGAAAGC
DcitrC025630.1.   123 CGAATCACTCGCTGCCAATTCCAACTTTGAAAACATCGCCAACGGCCTCAGTCTGAAAGC
consensus         301 ************************************************************

XM_026822511.1    361 GGTGGATGGAGATGGCAATATTCTTGGCGTTTCGCTCAACACTTGTCCCCTGGAGGGACG
DcitrC025630.1.   183 GGTGGATGGAGATGGCAATATTCTTGGCGTTTCGCTCAACACTTGTCCCCTGGAGGGACG
consensus         361 ************************************************************

XM_026822511.1    421 ACATATTATTGTCCGCAAAGAGATCGAAGGACGGCGGGAGATCATGGAGGAAATTATCAA
DcitrC025630.1.   243 ACATATTATTGTCCGCAAAGAGATCGAAGGACGGCGGGAGATCATGGAGGAAATTATCAA
consensus         421 ************************************************************

XM_026822511.1    481 ATTCCTCGGCCTCATCGACGAACTGTCTGGGGTTGAAAAACTCATCGAAAAGCATCACCA
DcitrC025630.1.   303 ATTCCTCGGCCTCATCGACGAACTGTCTGGGGTTGAAAAACTCATCGAAAAGCATCACCA
consensus         481 ************************************************************

XM_026822511.1    541 GAGTGTACTGGACGTCAAAATCCTTTCCGTCGACGCCAAACACCGGCAGAAAGGCATCGG
DcitrC025630.1.   363 GAGTGTACTGGACGTCAAAATCCTTTCCGTCGACGCCAAACACCGACAGAAAGGCATCGG
consensus         541 *********************************************.**************

XM_026822511.1    601 GGCTCGGCTGATCGAAGAGACTATCAAAACTGCTGAAGCACATGACATCAAGGCCATCAC
DcitrC025630.1.   423 GGCTCGGCTGATCGAAGAGACTATCAAAACTGCTGAAGCACATGACATCAAGGCCATCAC
consensus         601 ************************************************************
                                                              Forward Primer

XM_026822511.1    661 TGTGGGCTGCTCCAGCTACTACACTAGTGTGATCGTGCAGCATAAGTTCCCCAATCAGTT
DcitrC025630.1.   483 TGTGGGCTGCTCCAGCTACTACACAAGTATGATCGTGCAGCATAAGTTCCCCAATCAGTT
consensus         661 ************************ ***.*******************************

XM_026822511.1    721 TCGACGCGTGCTGACTTATCCATACTCCAAGTATGTTGACTCAAACGGAGAGGTGGTGTT
DcitrC025630.1.   543 TCGACGCGTGCTGACTTATCCGTACTCCAAGTATGTCGACTCAAAGGGAGAGGTGGTGTT
consensus         721 *********************.************** ******** **************

XM_026822511.1    781 CAAGTCAGCAGAGCCTCATGTGGAGTATCATGTGTATTTCTATGAGAGATAATTTCAAGA
DcitrC025630.1.   603 CAAGTCAGCAGAGCCTCATGTGGAGTATCATGTGTATTTCTATGAGAGATAA--------
consensus         781 ****************************************************........
                            Reverse Primer

XM_026822511.1    841 TGGTATCAGGAATGTGCGGAAGGAGATTATTTTCTATCGAAGTTACCTGTAAAAGCTATA
DcitrC025630.1.       ------------------------------------------------------------
consensus         841 ............................................................

XM_026822511.1    901 AAAGTAGGAATAGGGGTAGTATTTTTCGCTGGACGAAAAGAGAACTGCCCATTTGTAAAT
DcitrC025630.1.       ------------------------------------------------------------
consensus         901 ............................................................

XM_026822511.1    961 TATTTTTAGTTTTTAGGGTTCGAAGAGATAAAGTTACTTATTAGTACGTTGTTGGATTCA
DcitrC025630.1.       ------------------------------------------------------------
consensus         961 ............................................................
```

```
XM_026822511.1  1021 ACGAGCTTCAACTTCAATGTTAACGTCTAAAACTATAAGGTACAGGATACCAACGTTTGG
DcitrC025630.1.      ------------------------------------------------------------
consensus       1021 ............................................................

XM_026822511.1  1081 AAGATGATGTTTTCAAAAGTCTTGAAACGATGCTACTCGTAATGATACTGATGTTGCGTC
DcitrC025630.1.      ------------------------------------------------------------
consensus       1081 ............................................................

XM_026822511.1  1141 TGAGGATAAAACTTCTCGTGATATTTCTATGACAAGCTTGAAAATTAAAAAGTTTTGTAT
DcitrC025630.1.      ------------------------------------------------------------
consensus       1141 ............................................................

XM_026822511.1  1201 GGCTGAGGTTTTTAAAAAGTG
DcitrC025630.1.      ---------------------
consensus       1201 .....................
```

Figure S9. Multiple nucleotide sequence alignment of *DcAANAT*-1 from *Diaphorina citri*. Dopamine N-acetyltransferase-like isoform X1 (XM_026822511.1; 1221 bp) from NCBI database was aligned with Dopamine N-acetyltransferase (DcitrC025630.1.1; 654 bp), its top matched sequence from "*Diaphorina citri* OGS v2.0 proteins" BLAST datasets**.** Both genes are the top matched sequences of recent available data in GenBank, national center for biotechnology information website (NCBI, http://www.ncbi.nlm.nih.gov/gene/) and the "*Diaphorina citri* OGS v2.0 proteins" dataset available on Citrus Greening Solutions website (https://citrusgreening.org/organism/Diaphorina_citri/genome) using the Nucleotide-Nucleotide BLAST (BLASTn) and Protein-Protein BLAST (BLASTp) tools [4,10] using the NCBI arylalkylamine N-acetyltransferase 1, isoform A (GenBank Accession no. NP_523839.2) from fruit fly (*D. melanogaster*) [7–9] as a query sequences. Conserved amino acids are indicated with black shading and those with high similarity score are in gray. Forward and reverse primers are highlighted in yellow and blue colors, respectively. The full list of genes, names, and accession numbers are available in Table 1 and supplementary Tables S6 and S7.

```
XM_017443457.2      1 TGTACTCAATTCATTTTTATCAGCACAGAAGGAGTGGTCTTGTCTTTTTTAAGTTCGAAA
DcitrC085745.1.     1 ------------------------------------------------------------
consensus           1 ............................................................

XM_017443457.2     61 ATTCCTTTGAGAAAAGAAAAAGGCTTTTGGCTTGATTTCATTTTTAACTATCAATACAAA
DcitrC085745.1.     1 ----------------------------ATGGAGACAACAGCGCCCCCTACCGCCACCAT
consensus          61 .............................    **   **       *** *.  ** *

XM_017443457.2    121 GATGAGTAAAGAAGAAATAGACTACGTCTATCCCATTCCTGAAGACAAATACAACGACGT
DcitrC085745.1.    33 GTTGAACTAC---------TCCATCGTCCGTCTCTCTCCCTCCGACAAGGACCGCGTCAT
consensus         121 * ***.  * .........  *  **** .** *  ***    *****. ** .** *.*

XM_017443457.2    181 TATTGAACACTTACGCTACAACTTCTTCGCCGACGAACCTTTGAATAAATGTGTTGGTCT
DcitrC085745.1.    84 TGCT---TTCCTGGTCAAGTTCTTCTTTCATGACGAACCCCTGAATATTGCCGTGGGGCT
consensus         181 *. *...  * *.  * *   ******    ********  ******     ** ** **

XM_017443457.2    241 -TTGCGAAACAGGCAGAGGGCATTCGGAATTGGAACTACACAGTATTCTAACCCTACAGG
DcitrC085745.1.   141 GATGCAAGACGGACAGCGCTGTATCG-AGCTCGAGGACTACTGCACCACCCCCCTCGCAC
consensus         241 . ***.*.**.*.*** *     ***.*. * **.    ** * *      ****   .

XM_017443457.2    300 ATAATTTATCTGTCATGGCGGTCAATGGAAATGGACAGGTAATAGGCGTTG--CCCTGAA
DcitrC085745.1.   200 AAGGTCTGTCCTTGGCGGCTGTCAACGAGCGACAGGAGATTGTTGGCGTGTGTATCAATA
consensus         301 * ..* *.**  * . *** ***** *.. .  .. **.* .* *****  ..  * . *

XM_017443457.2    358 CGGCATTCAGCACGAAGGAGATGTGGATGAGGCCATCAAAAAGTTGG----AAACCCTCA
DcitrC085745.1.   260 CCATCAACTATCGGCGGGAATCGAGCACCGGACCTCCTGAATCTGGGGAAGATGAGTGTG
consensus         361 * .    * .   * .***.  * * *  .*.**  * .**  * **....* .     .

XM_017443457.2    414 ACGATAAGAAATTCAAACAGATTTTCTCCATGCTCTACGATCTCAACCAATCTCTCAACT
DcitrC085745.1.   320 CCCACCCAAAGTTCAAGATCATCCTGAAGTTCCTCAAATGGCTGGACAAGAAGAACGACA
consensus         421  * *   .**.*****.   **  *     * *** *  . ** .** *.     *.**

XM_017443457.2    474 TGTTCTCACGCTATGACGTCACATCTATTTTCGAATGTCGAATTCTGTCGGTGGATAACA
DcitrC085745.1.   380 TCTTCTCCAAGTTTAACATCAACAAGTATTTGGACATCAGCATTTTGTCCACAGATAGCG
consensus         481 * *****  . * *.**.***       *** **     * *** **** . .****.*.

XM_017443457.2    534 ACTATAGGGGGCGCGGACTGGCCAATGAACTGTTCAAATTGAGTATCGATATTGCGTCCA
DcitrC085745.1.   440 CTTATCGGGGCCAGGGCATTGCTAAGAAGCTGGTCTACGAGTCTATTGACTTGGCCGCGG
consensus         541   *** **** *. **  * ** ** .*.*** ** *   *  *** **  * **  * .

XM_017443457.2    594 AAGCTGGATTCAAGGTATTCAAGGTAGACGCGACTGGTGTATTCTCACAGAAAATCTCTA
DcitrC085745.1.   500 ACAACG-------------------ACATTCACCTGGTGAAGGCTGACTGTTCCTCGCAC
consensus         601 * .  *...................* *  *. ****** *  ** ** *    ** *

XM_017443457.2    654 CAAAGTTGGGACTAGAAACTCTGATCGAGTTGGAATATCGAAACCACCTAGATTCCGCCA
DcitrC085745.1.   541 TACT--------------------------------------------------CCGCCA
consensus         661  *  ..................................................******

XM_017443457.2    714 CTGGTCTACCAATGTTCACCCCACCCTCCCCTCATACCTCGCTGAAAGTCATGGTGAAAA
DcitrC085745.1.   551 AGG-----CGATGGCTAGGCTAGGCTTCTCCTGCATCTACAGCATGAGCTATGAGGACTA
consensus         721   *.....* *  * * . *  .  * ** ***    *  *.  . .**  ***. **  *

XM_017443457.2    774 TCCTGCAGTGAAGAGGTTGCAGGTTTGAATCTCATTGACCGTACCTTCCAAGAAGGAACA
DcitrC085745.1.   606 CACAGGATCAGACAACAAGCCGGTGTTCAGCCCGCCTGCG--CCACACCATGGAG-----
consensus         781   * * *  ..* *.   ** *** *  * * *.   .* .. *   *** *.**.....

XM_017443457.2    834 TTTAAATATGCGTTGGCTGTAACATAAAGCTTTCGTGAATATCTCTTGGTTTCATATGAA
DcitrC085745.1.   659 ----------------CTGAGACTTACG--------------------------------
consensus         841 ................*** .** ** .................................

XM_017443457.2    894 ACATTTTGGCTTTTAAATTGCTCTCTGAACCTGAGTTTAATATGAGAAGAATGGTTATTT
DcitrC085745.1.   671 ------------------------------------------------------------
consensus         901 ............................................................

XM_017443457.2    954 AAGTTATACTTAGAGTGTTTGTTGAGTTGATGTTATTATTCTAATAAACTTTAGTCTCTC
DcitrC085745.1.   671 --------------------------------------TTCTGGATGTCAAACGGTTCTT
consensus         961 ......................................****..  . *    *  ***

XM_017443457.2   1014 GGAGTTTAAAGTTAAAGTACTTAACACATTATCAGATTTAGATTTGTGCAAGAAGTTGAA
DcitrC085745.1.   693 GGAGCAGAGACCGAACATTCTGTCCGCTTGA-----------------------------
consensus        1021 ****   *.*   ** .* **   *.* * *.............................
```

```
XM_017443457.2  1074 TTATCAAGTACCTACTCAGATTACGTACCTAATTACCTATCTTTATTTCTAGTGAAAAAT
DcitrC085745.1.      ------------------------------------------------------------
consensus       1081 ............................................................

XM_017443457.2  1134 CTTTATGGTGGAGCTTTACACCGTAAATAACTCGAATTAATTTTGTAAATGCGCTATGAG
DcitrC085745.1.      ------------------------------------------------------------
consensus       1141 ............................................................

XM_017443457.2  1194 ATCTACCTCTGATGTATTTTCAATAGCTAGATGTTACCTAGACTATGTATTTTCATTCTA
DcitrC085745.1.      ------------------------------------------------------------
consensus       1201 ............................................................

XM_017443457.2  1254 AAATTAAAAACTTACGACTTATAATTTTATTCTTAATGGCCTGTGAAATTATGCTATTTT
DcitrC085745.1.      ------------------------------------------------------------
consensus       1261 ............................................................

XM_017443457.2  1314 ATGGTTCAGAATAGCACAATGAATTGCATGAGGAAATACATTATGAGATTTGTGAGGTGT
DcitrC085745.1.      ------------------------------------------------------------
consensus       1321 ............................................................

XM_017443457.2  1374 AATTTTTCAGGAGGAAAATCCTGGAGAGACTTGAAGGAGAATCAAAATTTAGGTGTATGT
DcitrC085745.1.      ------------------------------------------------------------
consensus       1381 ............................................................

XM_017443457.2  1434 TCTTGTTTTACACATAGACAGAGAACTTGAAAGCTGGGATTGACTTCCTGATTCTTTCCA
DcitrC085745.1.      ------------------------------------------------------------
consensus       1441 ............................................................

XM_017443457.2  1494 AATCTATTTTTGACTCAAAGTACAGCGGGATTAGTGAAGGAATTAGTACTGGAGCTAGCT
DcitrC085745.1.      ------------------------------------------------------------
consensus       1501 ............................................................

XM_017443457.2  1554 TAAGATGTATTTTTTTATGGAAGTTAATATGATCCATTATTCTGATCTCAGTTTAGGTGT
DcitrC085745.1.      ------------------------------------------------------------
consensus       1561 ............................................................

XM_017443457.2  1614 AAATTATTTACCAAGATGAAATAACGAATAGATTTCTGCTTTCCTCTAATTTGACTTTTT
DcitrC085745.1.      ------------------------------------------------------------
consensus       1621 ............................................................

XM_017443457.2  1674 CTAACTCTTTTGCTATCTGTGATAACCAATATTCTTGTTGACAATAAAAATAGAATTGAG
DcitrC085745.1.      ------------------------------------------------------------
consensus       1681 ............................................................

XM_017443457.2  1734 TAGAAGCAGTCATTGCACAGCACAAAAAAAAGTAAGCACAAAGAATGATGTTGTTATTAG
DcitrC085745.1.      ------------------------------------------------------------
consensus       1741 ............................................................

XM_017443457.2  1794 AGGTGGAGTTGAATTTAAGATTTCACTCAAGTAGTACAGGAAAGAAGGGATTGTCTAGAG
DcitrC085745.1.      ------------------------------------------------------------
consensus       1801 ............................................................

XM_017443457.2  1854 TTAATATTTTCTTCAAGTTGAGTGTGGATTAAGACATCAGTATTTTTTTTTTTTTTTT
DcitrC085745.1.      ----------------------------------------------------------
consensus       1861 ..........................................................
```

Figure S10. Multiple nucleotide sequence alignment of *DcAANAT*-2 from *Diaphorina citri*. Dopamine N-acetyltransferase-like (XM_017443457.2; 1911 bp) from NCBI database was aligned with Dopamine N-acetyltransferase (DcitrC085745.1.1; 723 bp), its top matched sequence from "*Diaphorina citri* OGS v2.0 proteins" BLAST datasets. Both genes are the top matched sequences of recent available data in GenBank, national center for biotechnology information website (NCBI, http://www.ncbi.nlm.nih.gov/gene/) and the "*Diaphorina citri* OGS v2.0 proteins" dataset available on Citrus Greening Solutions website (https://citrusgreening.org/organism/Diaphorina_citri/genome) using the Nucleotide-Nucleotide BLAST (BLASTn) and Protein-Protein BLAST (BLASTp) tools [4,10]

using the NCBI arylalkylamine N-acetyltransferase 1, isoform A (GenBank Accession no. NP_523839.2) from fruit fly (*D. melanogaster*) [7–9] as a query sequences. Conserved amino acids are indicated with black shading and those with high similarity score are in gray. The full list of genes, names, and accession numbers are available in Table 1 and supplementary Tables S6 and S7.

```
XP_026680472.1      1 ----------------------------------------------------------ML
DcitrP032285.1.     1 MKDRCSDIVSVDWLESMKHCPLDTLLRLYIYVHIYEICSNIVVSTHPDLKALTNQSATML
consensus           1 ..........................................................**

XP_026680472.1      3 EPYMGQLNNLKIVLASSSPRRSQILKSIGLKFEVIPSNFDESSIPVSKFKSNYGEYVSEL
DcitrP032285.1.    61 EPYMGQLNNLKIVLASSSPRRSQILKSIGLKFEVIPSNFDESSIPVSKFKNNYGEYVSEL
consensus          61 ************************************************** *********

XP_026680472.1     63 AYKKALEVSQHLKEDNVEPDLIIGADTVVSINDMMLGKPEDEEEAKEFLSKLSGNTHSVF
DcitrP032285.1.   121 AYKKALEVSQHLKEDNVEPDLIIGADTVVSINDMMLGKPKDEEEAKEFLSKLSGNTHSVF
consensus         121 *************************************** ********************

XP_026680472.1    123 TGVAILTKDKDSRFYNQTQVTFANLTPAVISAYVKTREPL--------------------
DcitrP032285.1.   181 TGVAILTKDKDSRFYNQTQVTFANLTPAVISAYVKTREPLDKAGAYGIQGIGGSLVEKVT
consensus         181 ****************************************....................

XP_026680472.1        ----------------------------------
DcitrP032285.1.   241 GDPFNVVGFPLSAFCSHLVEKVLPQDSLKHILEH
consensus         241 ..................................
```

Figure S11. Multiple AA sequence alignment of *DcASMT* from *Diaphorina citri*. Septum formation protein Maf-like (XP_026680472.1; 162 aa) from NCBI database were aligned with N-acetylserotonin O-methyltransferase-like (DcitrP032285.1.1; 274 aa), its top matched sequence from "*Diaphorina citri* OGS v2.0 proteins" BLAST datasets. Both genes are the top matched sequences of recent available data in GenBank, national center for biotechnology information website (NCBI, http://www.ncbi.nlm.nih.gov/gene/) and the "*Diaphorina citri* OGS v2.0 proteins" dataset available on Citrus Greening Solutions website (https://citrusgreening.org/organism/Diaphorina_citri/genome) using the Nucleotide-Nucleotide BLAST (BLASTn) and Protein-Protein BLAST (BLASTp) tools [4,10] using the NCBI *N*-acetylserotonin O-methyltransferase-like protein - isoform X1 (GenBank Accession no. XP_014251646.1) from bed bug (*Cimex lectularius*) as a query sequences. Conserved amino acids are indicated with black shading and those with high similarity score are in gray. The full list of genes, names, and accession numbers are available in Table 1 and supplementary Tables S8 and S9.

```
XM_026824671.1        1 TCAAATTTTTGTGAAACAACAGAAACACCAAAGGACGGACTTGATTCCAGACTTTCCTCT
DcitrC032285.1.       1 ---------------------------ATGAAAGACAG----------------------
consensus             1 ...........................  .**.***.*......................

XM_026824671.1       61 TTCATCCTGATTCCTTGCTCTAGCTTTTGAGGTGTTCAGATATTGCATCAGTGGATTGGT
DcitrC032285.1.      12 -------------------------------GTGTTCAGATATTGTATCAGTGGATTGGT
consensus            61 ...............................************** **************

XM_026824671.1      121 TGGAGTCAATGAAACATTGTCCATTGGATACCCTATTGAGG----------------TTA
DcitrC032285.1.      41 TGGAGTCAATGAAACATTGTCCATTGGATACCCTATTGAGGTTATATATATATGTTCATA
consensus           121 *****************************************................ **

XM_026824671.1      165 TATAGGAAATTTATTCCAACATATAGTTGTATCAACTCATCCTGATTTGAAAGCACTGAC
DcitrC032285.1.     101 TATATGAAATTTGTTCCAACAT--AGTTGTATCAACTCATCCTGATTTGAAAGCACTGAC
consensus           181 **** *******.*********..************************************

XM_026824671.1      225 TAACCAGTCAGCAACCATGCTTGAACCATATATGGGCCAATTGAACAATTTGAAAATTGT
DcitrC032285.1.     159 CAACCAGTCAGCAACCATGCTTGAACCATATATGGGCCAATTGAACAATTTGAAAATTGT
consensus           241  ***********************************************************

XM_026824671.1      285 CCTGGCAAGCAGTTCTCCTAGGAGATCACAAATTTTGAAATCAATTGGCCTTAAATTTGA
DcitrC032285.1.     219 CCTGGCAAGCAGTTCTCCTAGGAGATCACAAATTTTGAAATCAATTGGCCTTAAATTTGA
consensus           301 ************************************************************
                        Forward Primer

XM_026824671.1      345 AGTCATTCCATCAAACTTTGATGAAAGCTCCATTCCAGTGTCCAAATTCAAGAGCAACTA
DcitrC032285.1.     279 AGTCATTCCATCAAACTTTGATGAAAGCTCCATTCCAGTGTCCAAATTCAAGAACAACTA
consensus           361 *****************************************************.******

XM_026824671.1      405 TGGTGAGTATGTATCTGAGTTAGCCTACAAGAAAGCTCTGGAAGTTTCTCAGCACCTGAA
DcitrC032285.1.     339 TGGTGAGTATGTATCTGAGTTAGCCTACAAGAAAGCTCTGGAAGTTTCTCAGCACCTGAA
consensus           421 ************************************************************

XM_026824671.1      465 AGAAGACAATGTTGAGCCGGATTTAATCATTGGTGCTGATACTGTTGTGTCTATCAATGA
DcitrC032285.1.     399 AGAAGACAATGTTGAGCCGGATTTAATCATTGGTGCTGATACTGTTGTGTCTATCAATGA
consensus           481 ************************************************************
                            Reverse Primer

XM_026824671.1      525 TATGATGCTGGGAAAACCTGAGGATGAAGAAGAAGCCAAGGAGTTTCTCTCCAAATTGAG
DcitrC032285.1.     459 TATGATGCTGGGAAAACCTAAGGATGAAGAAGAAGCCAAGGAGTTTCTCTCCAAATTGAG
consensus           541 *******************.****************************************

XM_026824671.1      585 TGGGAACACTCACTCTGTGTTCACTGGAGTGGCTATTCTAACCAAAGACAAAGACTCAAG
DcitrC032285.1.     519 TGGGAACACTCACTCTGTGTTCACTGGAGTGGCTATTCTAACCAAAGACAAAGACTCAAG
consensus           601 ************************************************************

XM_026824671.1      645 GTTTTACAATCAAACTCAAGTGACGTTCGCCAATTTGACACCTGCTGTGATATCTGCCTA
DcitrC032285.1.     579 GTTTTACAATCAAACTCAAGTGACGTTCGCCAATTTGACACCTGCTGTGATATCTGCCTA
consensus           661 ************************************************************

XM_026824671.1      705 TGTGAAAACTCGAGAACCATTG---------TAAGCTTTAGG------------------
DcitrC032285.1.     639 TGTGAAAACTCGAGAACCATTGGATAAAGCAGGCGCCTATGGGATACAAGGAATAGGAGG
consensus           721 **********************......... . ** *  **..................

XM_026824671.1      738 ------------------------------------------------------------
DcitrC032285.1.     699 TTCACTGGTGGAGAAGGTAACTGGAGATCCTTTCAATGTTGTAGGATTCCCTCTGAGCGC
consensus           781 ............................................................

XM_026824671.1      738 ---------------------GAAAGAGCT------------------------------
DcitrC032285.1.     759 ATTCTGTTCTCACTTGGTGGAGAAAGTACTCCCACAAGACTCTTTGAAACATATCTTGGA
consensus           841 .....................***** .**..............................

XM_026824671.1          -------
DcitrC032285.1.     819 ACATTGA
consensus           901 .......
```

Figure S12. Multiple nucleotide sequence alignment of *DcASMT* from *Diaphorina citri*. Septum formation protein Maf-like (XM_026824671.1; 746 bp) from NCBI database were aligned with N-acetylserotonin O-methyltransferase-like (DcitrC032285.1.1; 825 bp), its top matched sequence from "*Diaphorina citri* OGS v2.0 proteins"

BLAST datasets. Both genes are the top matched sequences of recent available data in GenBank, national center for biotechnology information website (NCBI, http://www.ncbi.nlm.nih.gov/gene/) and the "*Diaphorina citri* OGS v2.0 proteins" dataset available on Citrus Greening Solutions website (https://citrusgreening.org/organism/Diaphorina_citri/genome) using the Nucleotide-Nucleotide BLAST (BLASTn) and Protein-Protein BLAST (BLASTp) tools [4,10] using the NCBI- *N*-acetylserotonin O-methyltransferase-like protein - isoform X1 (GenBank Accession no. XP_014251646.1) from bed bug (*Cimex lectularius*) as a query sequences. Conserved amino acids are indicated with black shading and those with high similarity score are in gray. Forward and reverse primers are highlighted in yellow and blue colors, respectively. The full list of genes, names, and accession numbers are available in Table 1 and supplementary Tables S8 and S9.

References

1. Tiwari, S.; Gondhalekar, A.D.; Mann, R.S.; Scharf, M.E.; Stelinski, L.L. Characterization of five CYP4 genes from Asian citrus psyllid and their expression levels in *Candidatus* Liberibacter asiaticus-infected and uninfected psyllids. *Insect Mol. Biol.* **2011**, *20*, 733–744.

2. Killiny, N.; Nehela, Y.; Hijaz, F.; Vincent, C.I. A plant pathogenic bacterium exploits the tricarboxylic acid cycle metabolic pathway of its insect vector. *Virulence* **2018**, *9*, 99–109.

3. Coleman, C.M.; Neckameyer, W.S. Serotonin synthesis by two distinct enzymes in *Drosophila melanogaster*. *Arch. Insect Biochem. Physiol.* **2005**, *59*, 12–31.

4. Altschul, S.F.; Wootton, J.C.; Gertz, E.M.; Agarwala, R.; Morgulis, A.; Schaffer, A.A.; Yu, Y.-K. Protein database searches using compositionally adjusted substitution matrices. *FEBS J.* **2005**, *272*, 5101–5109.

5. Hirsh, J.; Davidson, N. Isolation and characterization of the dopa decarboxylase gene of *Drosophila melanogaster*. *Mol. Cell. Biol.* **1981**, *1*, 475–485.

6. Tatarenkov, A.; Ayala, F.J. Nucleotide variation at the dopa decarboxylase (Ddc) gene in natural populations of *Drosophila melanogaster*. *J. Genet.* **2007**, *86*, 125–137.

7. Hintermann, E.; Jenö, P.; Meyer, U.A. Isolation and characterization of an arylalkylamine *N*-acetyltransferase from *Drosophila melanogaster*. *FEBS Lett.* **1995**, *375*, 148–150.

8. Hintermann, E.; Grieder, N.C.; Amherd, R.; Brodbeck, D.; Meyer, U.A. Cloning of an arylalkylamine N-acetyltransferase (aaNAT1) from *Drosophila melanogaster* expressed in the nervous system and the gut. *Proc. Natl. Acad. Sci. U. S. A.* **1996**, *93*, 12315–12320.

9. Dempsey, D.R.; Jeffries, K.A.; Bond, J.D.; Carpenter, A.M.; Rodriguez-Ospina, S.; Breydo, L.; Caswell, K.K.; Merkler, D.J. Mechanistic and structural analysis of *Drosophila melanogaster* arylalkylamine N-acetyltransferases. *Biochemistry* **2014**, *53*, 7777–7793.

10. Altschul, S.F.; Gish, W.; Miller, W.; Myers, E.W.; Lipman, D.J. Basic local alignment search tool. *J. Mol. Biol.* **1990**, *215*, 403–410.
